# Third generation vs first generation EGFR-TKIs in the first line treatment for EGFR-mutated locally advanced or metastatic non-small cell lung cancer: a meta-analysis based on randomized controlled trials

**DOI:** 10.7150/jca.99319

**Published:** 2025-01-01

**Authors:** Wenjie Hu, Yi Qin, Taoming Dong, Xueying Lin, Yuan Chen, Wenxiong Zhang, Tanggui Feng

**Affiliations:** 1 Department of Oncology, The Second People's Hospital of Jingdezhen, Jingdezhen, China.; 2 Department of Respiratory and Critical Care Medicine, The Second People's Hospital of Jingdezhen, Jingdezhen, China.; 3 Department of thoracic surgery, The Second Affiliated Hospital, Jiangxi Medical College, Nanchang University, Nanchang, China.

**Keywords:** Tyrosine kinase inhibitors, Third-generation, First-generation, Non-small cell lung cancer, Meta-analysis

## Abstract

**Background:** The prevailing belief is that third-generation tyrosine kinase inhibitors (TKIs) targeting the epidermal growth factor receptor (EGFR) (TGET) outperform first-generation EGFR-TKIs (FGET) in managing advanced-stage EGFR-mutated non-small cell lung cancer (NSCLC). However, this standpoint lacks substantiation in evidence-based medicine. Therefore, this meta-analysis was conducted to compare the efficacy and adverse effects (AEs) of these two categories.

**Methods:** We searched seven databases for relevant randomized controlled trials (RCTs), focusing on primary endpoints such as progression-free survival (PFS), overall survival (OS), and central nervous system PFS (CNS-PFS). Additional factors considered included treatment responses and AEs.

**Results:** We analyzed 15 studies from 6 RCTs on six third-generation TKIs: Osimertinib, Lazertinib, Furmonertinib, Aumolertinib, Naquotinib, and Befotertinib. TGET showed better efficacy in PFS (hazard ratio [HR]: 0.55 [0.41, 0.75]), CNS-PFS (HR: 0.48 [0.35, 0.66]), CNS-objective response rate (CNS-ORR, risk ratio [RR]: 1.40 [1.19, 1.65]), and duration of response (DOR, HR: 0.52 [0.38, 0.72]). Most subgroups confirmed the PFS advantage. With longer survival time, the superiority in PFS, OS, and CNS-PFS of TGETs became more evident. Both groups had similar OS (HR: 0.86), ORR, CNS-DOR, total AEs, and grade 3-5 AEs. However, TGETs had more severe AEs (RR: 1.17 [1.02, 1.35]). Additionally, there were more grade 3-4 cases of diarrhea, decreased platelet count, pulmonary embolism, fatigue, decreased neutrophil count, and rash, and fewer grade 3-4 increases in alanine transaminase (ALT) and aspartate transaminase (AST) in the TGET group. The top 5 AEs in the TGET group were diarrhea (36.32%), rash (30.24%), decreased platelet count (29.15%), elevated serum creatinine (23.63%), and decreased white blood cell count (22.02%).

**Conclusions:** Except for Naquotinib, TGETs demonstrate superiority over FGETs in treating EGFR-mutated locally advanced or metastatic NSCLC, showing improved survival and responses. However, the increased incidence of AEs necessitates careful consideration.

## Introduction

During the past decade, 80% of diagnosed cases of non-small cell lung cancer (NSCLC) were either locally advanced or metastatic, significantly contributing to cancer-related mortality 1,2. Approximately 51.4% of all NSCLC cases are attributed to mutations in the epidermal growth factor receptor (EGFR) 3. The standard treatment approach for EGFR-mutated NSCLC is EGFR-tyrosine kinase inhibitors (TKIs) 4. Over a decade ago, first-generation TKIs such as Erlotinib and Gefitinib were employed in the treatment of EGFR-mutated NSCLC, confirming their superior efficacy and safety compared to chemotherapy 5. However, their ability to prolong patient survival remains unsatisfactory. With subsequent drug iterations, third-generation TKIs have increasingly been utilized as first-line treatments for EGFR-mutated advanced NSCLC in recent years 6-11. Many perspectives suggest that the first-line use of TGETs may lead to better clinical outcomes 12.

In the latest versions of the NCCN Clinical Practice Guidelines and ESMO Clinical Practice Guidelines, both TGET and FGET are recommended for the first-line treatment of EGFR-mutated advanced NSCLC 4,13. Studies such as FLAURA (Osimertinib) and LASER301 (Lazertinib) reported better survival outcomes, including progression-free survival (PFS), in the TGET group 6,7. Similar results were confirmed by trials such as FURLONG (Furmonertinib) and AENEAS (Aumolertinib) 8,9. Lu *et al.* reported that although the third-generation TKI (Befotertinib) may exhibit superior survival efficacy, it is associated with a higher incidence of total/grade 3-5 adverse effects (AEs) 11. However, the SOLAR trial (Naquotinib) reported worse survival outcomes and a higher incidence of grade 3-5 AEs in the TGET group 10.

To address this clinical controversy, this meta-analysis was conducted to compare the two groups in terms of survival, responses, and safety.

## Materials and Methods

In compliance with PRISMA guidelines, this study was registered in PROSPERO (ID: CRD42024533158) and conducted accordingly (**Table S1**).

### Search strategy

The search strategy utilized the keywords 'lung cancer,' 'randomized,' and TGETs (including Osimertinib, Nazartinib, Rociletinib, Mavelertinib, Lazertinib, Olmutinib, Naquotinib, Almonertinib, Furmonertinib, Abivertinib, Rezivertinib, Limertinib, Befotertinib, Olafertinib, Keynatinib, Oritinib, and TAS-121). Thorough searches were conducted across seven databases (PubMed, ScienceDirect, Ovid MEDLINE, the Cochrane Library, Scopus, EMBASE, and Web of Science) for eligible RCTs from the inception of each database until January 20, 2024 (**Table S2**). Additionally, the reference lists of the included RCTs were reviewed to identify additional eligible studies.

### Selection criteria

English-published studies were chosen based on PICOS criteria:

(1) Participants (P): patients with EGFR-mutated advanced NSCLC.

(2) Intervention (I): first line treatment with TGET.

(3) Control (C): first line treatment with FGET.

(4) Outcomes (O): survival (PFS, overall survival [OS], central nervous system PFS [CNS-PFS]), responses, and AEs.

(5) Study design (S): RCTs.

Articles lacking primary data, as well as meta-analyses and case reports, were excluded. Multiple studies reporting on the same trial with diverse outcomes were included, but only the most recent data were used for identical outcomes in the analysis.

### Data extraction

Two investigators independently collected data, including study characteristics (publication date, first author, etc.), participant details (sex, age, etc.), cancer specifics (histopathology, stage, etc.), antitumor effectiveness (PFS, OS, CNS-PFS, responses, etc.), and adverse event counts (total AEs, serious AEs, etc.). Any discrepancies were resolved through re-evaluation and discussion.

### Outcome assessments

The primary endpoints analyzed were PFS, OS, and CNS-PFS. Additionally, we examined PFS within specific subgroups: Age (<65 or >65 years), Sex (Female or Male), Smoking status (Active/Former smoker or Non-smoker), Eastern Cooperative Oncology Group (ECOG) PS (0 or 1), CNS metastases at baseline (Yes or No), EGFR mutation (Ex19del or L858R), Race category (Asian or Non-Asian), and TGETs (Osimertinib, Lazertinib, Furmonertinib, Aumolertinib, Befotertinib, or Naquotinib). Meanwhile, comparisons were conducted between the two groups for PFS rate (PFSR), OS rate (OSR), and CNS-PFS rate (CNS-PFSR) at 6-36 months. The PFSR was also analyzed in subgroups according to CNS metastases at baseline (Yes or No) and EGFR mutation (Ex19del or L858R).

### Quality assessment

We evaluated RCT quality using both the Jadad scale, a 5-point system reflecting randomization, blinding, and patient inclusion, with ≥3 points considered indicative of high quality 14, and the Cochrane Risk Assessment Tool, categorizing risk as low, unclear, or high for bias related to selection, performance, detection, attrition, and reporting 15. The bias graph illustrates the findings.

The quality of the results was assessed using the Grading of Recommendations, Assessment, Development, and Evaluation (GRADE) method, primarily considering bias, indirectness, inaccuracy, and publication bias. Outcomes are categorized into four levels: very low, low, medium, and high 16.

### Statistical analysis

Pooled data were evaluated using Review Manager 5.3. For the analysis of survival data (PFS, OS, CNS-PFS, etc.), hazard ratio (HR) was employed. Favorable outcomes were indicated by an HR < 1 in the TGET group. Dichotomous variables (PFSR, OSR, ORR, AEs, etc.) were analyzed using risk ratio (RR). Mean difference (MD) was used for analyzing continuous variables (depth of response, etc.). Heterogeneity was assessed using the *I*^2^ statistic and *χ*2 test. When *I*^2^ was less than 50% or P was greater than 0.1, indicating no significant heterogeneity, a fixed-effects model was applied; otherwise, a random-effects model was used. Publication bias was assessed by visually examining funnel plots. A p-value < 0.05 denoted statistical significance.

## Results

### Search results

**Figure 1:**
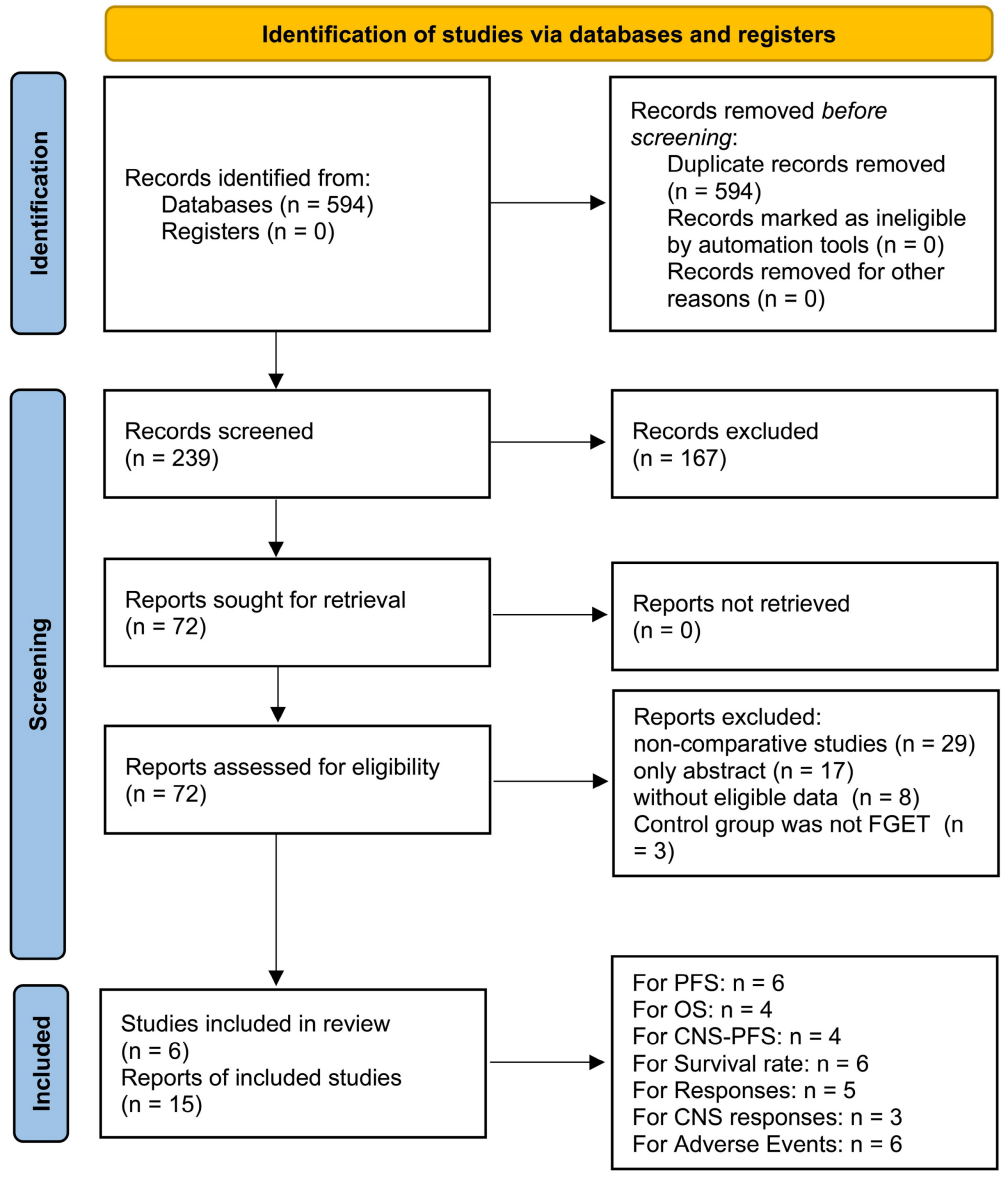
Study selection flow.

In the final analysis, fifteen studies based on six RCTs investigating 6 TGETs (Osimertinib, Lazertinib, Furmonertinib, Aumolertinib, Naquotinib, and Befotertinib) were included (**Figure 1**) 6-11,17-25. The TGET group comprised 1316 patients, and the FGET group included 1311 patients. All six RCTs were of high quality according to the Jadad scale and Cochrane Risk Assessment Tool (**Figure S1, Table S3**). The quality of all results fell within the medium-high range as per the GRADE method (**Table S4**). **Table 1** summarizes the baseline information for the included studies. At the time of data cutoff, 397 patients (30.17%) continued treatment in the TGET group, and 145 patients (11.06%) continued treatment in the FGET group (**Figure S2**).

### Survival

**Figure 2:**
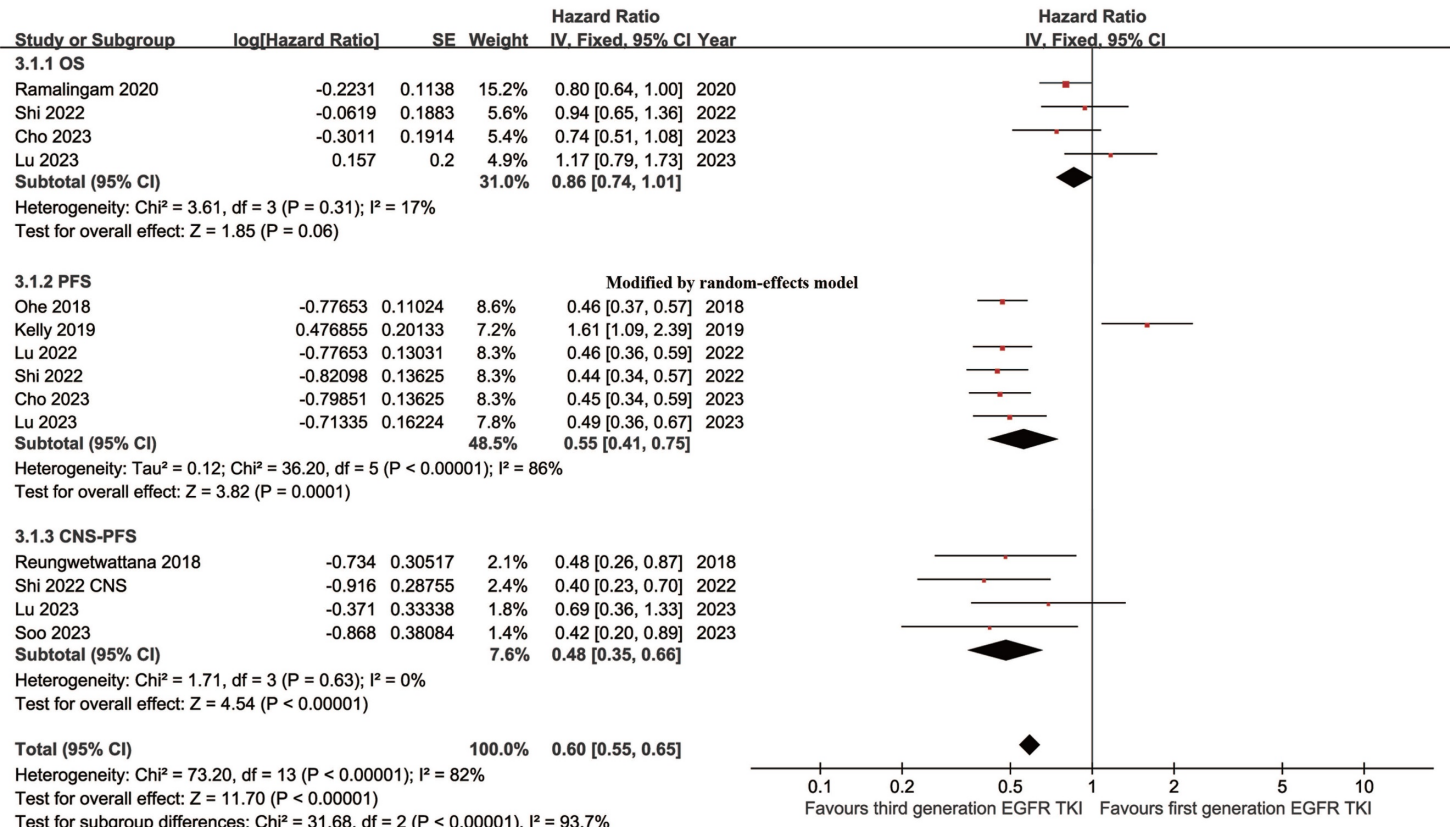
Forest plots of OS, PFS, and CNS-PFS associated with TGET versus FGET.

**Figure 3:**
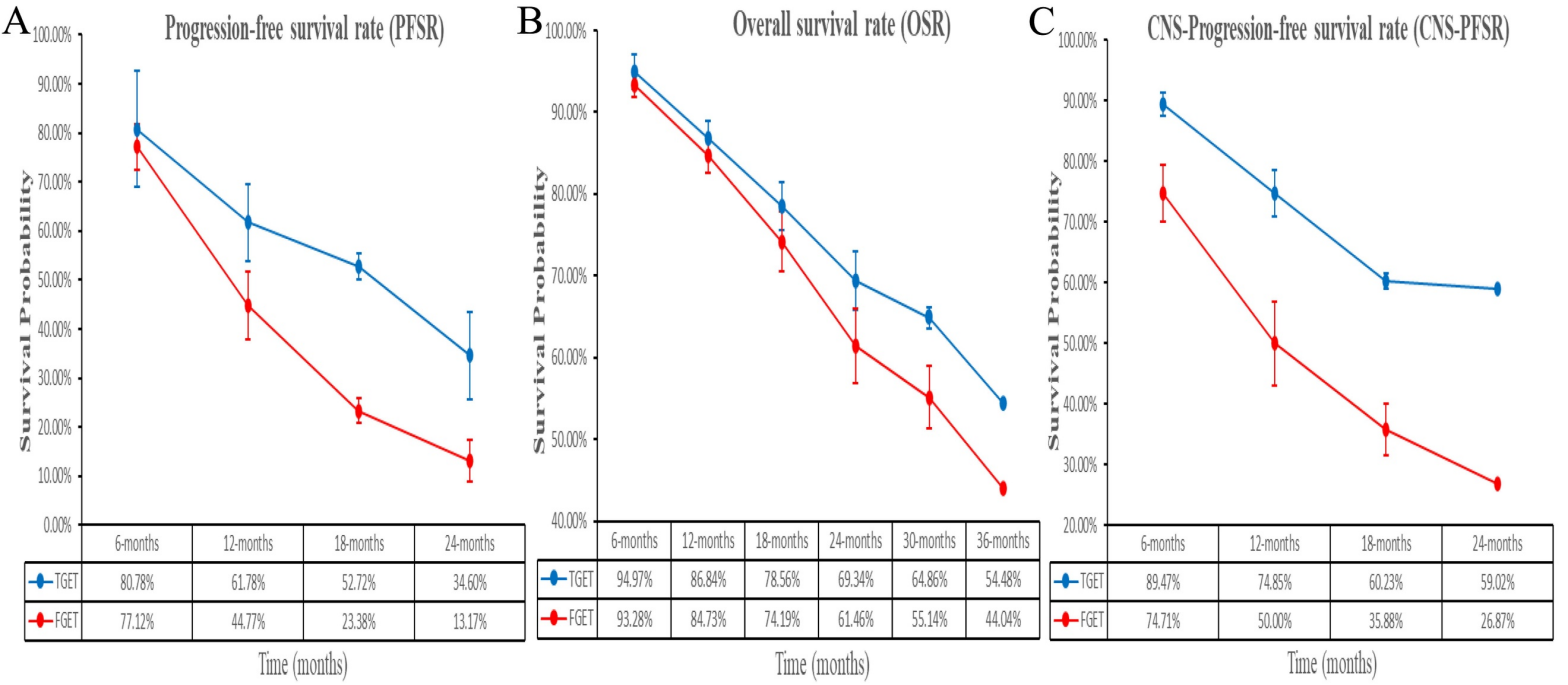
Comparisons of PFSR (6-24 months), OSR (3-36 months), and CNS-PFSR (6-24 months) associated with TGET versus FGET.

**Figure 4:**
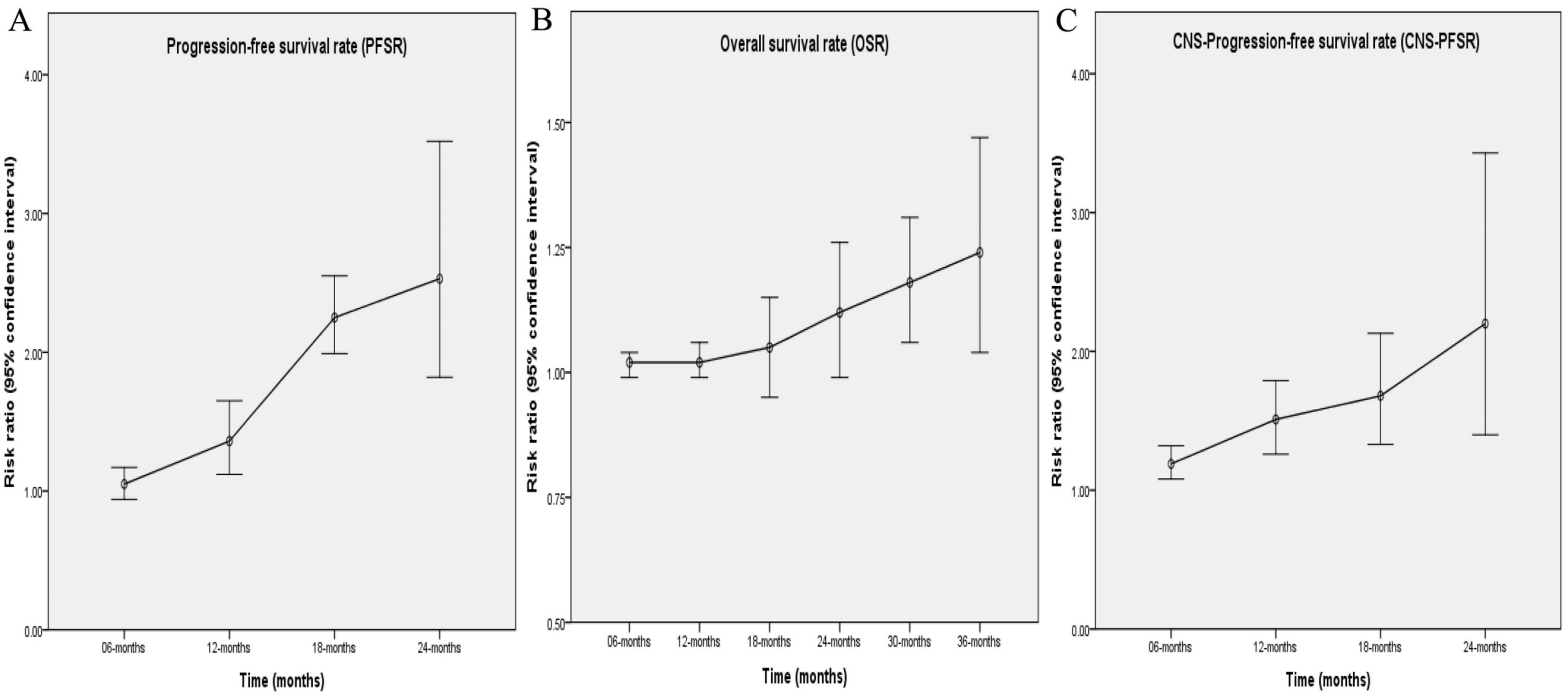
Trend of risk ratios in the comparisons of PFSR (6-24 months), OSR (3-36 months), and CNS-PFSR (6-24 months) associated with TGET versus FGET.

Better PFS was found in the TGET group (HR: 0.55 [0.41, 0.75], p = 0.0001; **Figure 2**). At 12-24 months, PFSR favored the TGET group (**Figure S3**). In terms of extended survival, TGET displayed a growing advantage in PFSR compared to FGET (**Figure 3A, 4A**).

OS tended to favor the TGET group without statistical significance (HR: 0.86 [0.74, 1.01], p = 0.06; **Figure 2**). At 30-36 months, OSR favored the TGET group (**Figure S4**). In terms of extended survival, TGET displayed a growing advantage in OSR compared to FGET (**Figure 3B, 4B**).

Better CNS-PFS was found in the TGET group (HR: 0.48 [0.35, 0.66], p < 0.00001; **Figure 2**). At 6-24 months, CNS-PFSR favored the TGET group (**Figure S5**). In terms of extended survival, TGET displayed a growing advantage in CNS-PFSR compared to FGET (**Figure 3C, 4C**).

### Subgroup analysis

**Figure 5:**
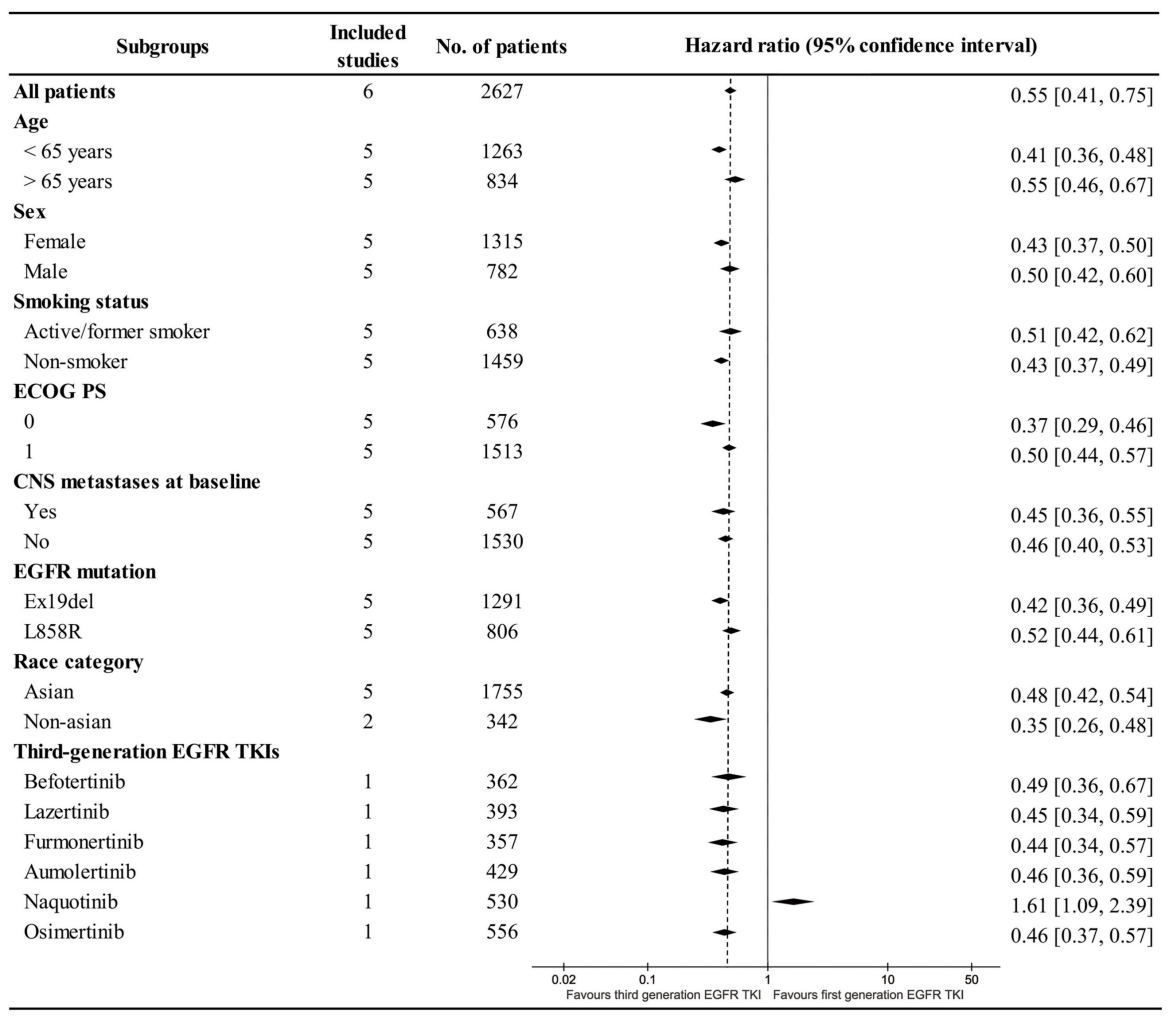
Subgroup analysis of PFS.

Subgroup analysis showed that PFS was better with TGETs in most groups. Age (< 65 years), sex (Female), smoking status (Non-smoker), ECOG PS (0), EGFR mutation (Ex19del), and race category (Non-Asian) might benefit more from TGET treatment. However, in the subgroup of TGET-Naquotinib, PFS tended to favor the FGET group (**Figure 5**).

PFSR tends to favor the TGET group in all subgroups (with or without CNS metastases, Ex19del or L858R mutations) at 6-24 months (**Figure S6-S10**). Meanwhile, in terms of extended survival, TGET shows an increasing advantage in PFSR compared to FGET in all subgroups (**Figure S11**).

### Responses

In the response analysis, the ORR (RR: 0.98 [0.90, 1.07]), DCR (RR: 0.99 [0.96, 1.03]), CR (RR: 1.66 [0.61, 4.54]), PR (RR: 0.98 [0.90, 1.06]), SD (RR: 1.01 [0.79, 1.29]), and PD (RR: 0.86 [0.42, 1.77]) showed similarity between the two groups (**Figure S12**). The TGET group had favorable outcomes in terms of duration of response (DOR, HR: 0.52 [0.38, 0.72]) and depth of response (MD: -0.05 [-0.06, -0.04] %) (**Figure S13, S14**). The TGET group also exhibited a higher estimated percentage remaining in response (EPR) at 6-24 months (**Figure S15**).

**Figure 6:**
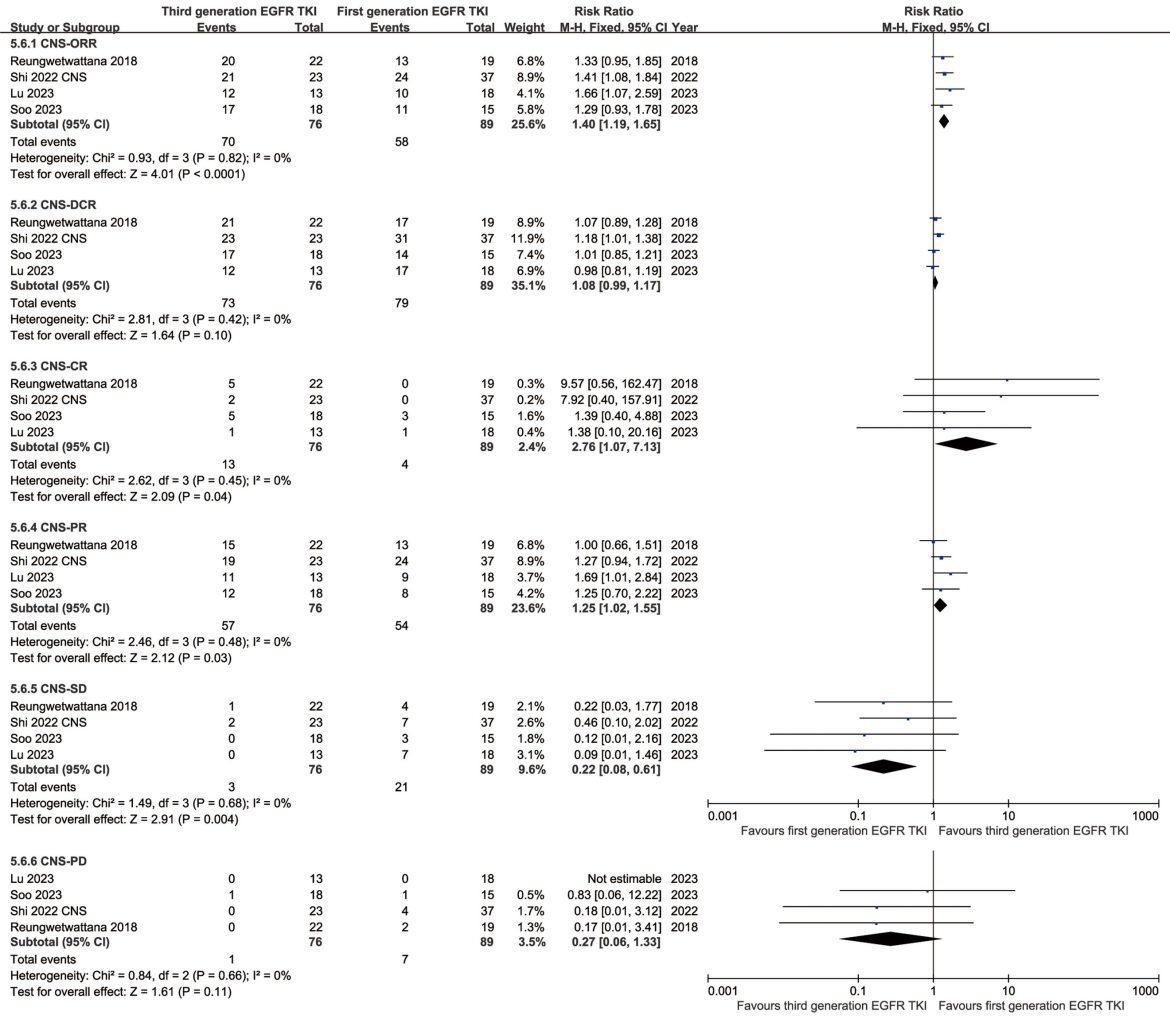
Forest plots of CNS responses (ORR, DCR, CR, PR, SD, and PD) associated with TGET versus FGET.

In the analysis of CNS responses, the TGET group tended to surpass the FGET group in CNS-ORR (RR: 1.40 [1.19, 1.65]), CNS-DCR (RR: 1.08 [0.99, 1.17]), CNS-CR (RR: 2.76 [1.07, 7.13]), and CNS-PR (RR: 1.25 [1.02, 1.55]). Conversely, the TGET group showed lower CNS-SD (RR: 0.22 [0.08, 0.61]) and CNS-PD (RR: 0.27 [0.06, 1.33]) (**Figure 6**). While the CNS-DOR (HR: 0.73 [0.26, 2.08]) and depth of response (MD: -0.12 [-0.28, 0.04] %) tended to favor the TGET group, statistical significance was not observed (**Figure S13, S14**). Similarly, the estimated percentage remaining in CNS response (CNS-EPR) favored the TGET group at 6-24 months without statistical significance (**Figure S16**).

### Toxicity

In summary, total AEs (RR: 0.99 [0.98, 1.01]), grade 3-5 AEs (RR: 1.10 [0.93, 1.29]), fatal AEs (RR: 1.22 [0.81, 1.83]), treatment-related AEs (RR: 1.01 [0.96, 1.06]), grade 3-5 treatment-related AEs (RR: 1.24 [0.73, 2.12]), serious treatment-related AEs (RR: 1.15 [0.62, 2.12]), fatal treatment-related AEs (RR: 2.10 [0.47, 9.32]), discontinuation due to AEs (RR: 1.20 [0.79, 1.84]), dose reduction due to AEs (RR: 1.58 [0.81, 3.09]), dose interruption due to AEs (RR: 1.04 [0.84, 1.29]) were similar between the two groups. More serious AEs (RR: 1.17 [1.02, 1.35]) were found in the TGET group (**Table 2**).

In analyzing AEs of any grade, more occurrences of platelet count decrease, elevated serum creatinine, white blood cell count decrease, peripheral sensory neuropathy, deep vein thrombosis, hyponatremia, lipid metabolism diseases, renal symptoms, hyperuricemia, anemia, upper respiratory tract infection, headache, fatigue, constipation, elevated fibrin D-dimer, neutrophil count decrease, muscle spasms, blood lactate dehydrogenase increase, vomiting, nasopharyngitis, dyspnea, ECG QTc prolongation, lymphocyte count decrease, edema, gastrointestinal diseases, and pulmonary embolism were found in the TGET group. More rash, ALT increase, AST increase, hypokalemia, gamma-glutamyl transferase increase, and blood bilirubin increase were found in the FGET group (**Table S5**). AEs of any grade, exceeding a 10% occurrence rate, were listed in **Table 3**.

In analyzing grade 3-5 AEs, more diarrhea, platelet count decrease, pulmonary embolism, fatigue, and neutrophil count decrease were found in the TGET group. More ALT increase, AST increase, and rash were found in the FGET group (**Table S6**). Grade 3-5 AEs, exceeding a 1% occurrence rate, were listed in **Table 4**.

### Sensitivity analysis

The analysis of PFS, ORR, DCR, DOR, total AEs, and grade 3-5 AEs revealed notable heterogeneity. Omitting any study did not alter the stability or reliability of the results according to the sensitivity analysis (**Figure S17**).

### Publication bias

**Figure 7:**
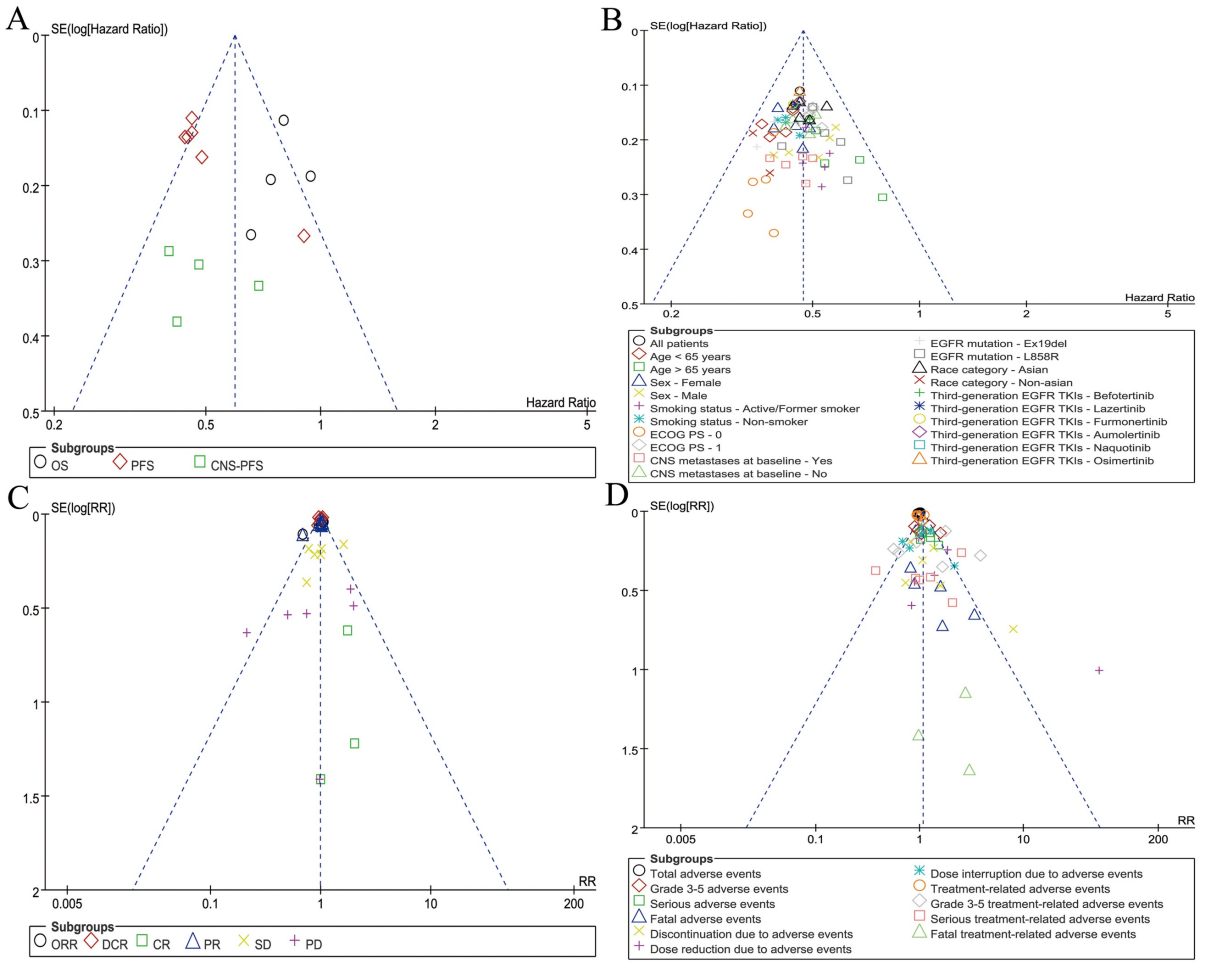
Funnel plots of survival summary (A), subgroup analysis of PFS (B), responses (C), and summary of AEs (D) associated with TGET versus FGET.

Symmetrical funnel plots were observed for survival summary (**Figure 7A**), subgroup analysis of PFS (**Figure 7B**), responses (**Figure 7C**), and summary of AEs (**Figure 7D**), indicating acceptable publication bias.

## Discussion

With the introduction of various TGETs, an increasing number of patients with EGFR-mutated locally advanced or metastatic NSCLC are receiving first-line treatment with these agents. The superiority of TGETs over FGETs for patients in this stage is widely recognized in clinical practice 26,27. However, whether this perspective is accurate, whether it applies to all TGETs, where the superiority of TGETs over FGETs lies, and what limitations exist remain under-explored in evidence-based medicine 28,29. This study represents the first meta-analysis comparing TGET with FGET for advanced NSCLC. The results suggest that TGET achieves superior efficacy in PFS, CNS-PFS, CNS-ORR, and DOR. The survival advantage of PFS was confirmed in almost all subgroups. Similar OS, ORR, CNS-DOR, total AEs, grade 3-5 AEs, and fatal AEs were found between the two groups. However, more serious AEs were found in the TGET group.

Prolongation of survival time is widely recognized as the most important reason for the acceptance of third-generation drugs. In recent years, some drugs have been shown to significantly improve PFS but fail to improve OS, a phenomenon that has been perplexing 11,30. In this study, we found that the TGET group exhibited higher PFS and CNS-PFS compared to the FGET group and tended to have higher OS without statistical significance. Osimertinib was the only TGET to demonstrate a positive OS outcome 18. The advantage in PFS was observed in almost all TGETs, except for Naquotinib. The SOLAR study was prematurely terminated due to the inferior PFS of Naquotinib compared to FGET 10.

Additionally, we found that the DOR and depth of response were significantly superior in the TGET group compared with the FGET group. Three reasons may explain this survival advantage: 1. TGETs have better blood-brain barrier permeability, leading to significantly improved control of intracranial metastases due to higher drug solubility in the brain 31,32; 2. TGETs also have a certain therapeutic effect on the T790M mutation after conventional Ex19del and L858R mutations, which may prolong the duration of drug response 33,34; 3. TGETs not only inhibit the tyrosine kinase activity of EGFR but also have inhibitory effects on multiple other targets, allowing for a more comprehensive blockade of tumor cell signaling pathways, effectively inhibiting tumor cell growth and spread 35. In subgroup analysis of PFS, PFS tended to favor the TGET group across most subgroups. Age (< 65 years), sex (Female), smoking status (Non-smoker), ECOG PS (0), EGFR mutation (Ex19del), and race category (Non-Asian) might benefit more from TGET treatment.

Brain metastases occur at a significantly high rate in patients with advanced NSCLC (up to 40%) 36. Additionally, the prognosis for patients with brain metastases is often poor. Therefore, controlling brain metastases is of utmost importance and greatly influences the OS of patients at this stage of cancer 37. Our study found that the notable survival advantage of TGET over FGET lies in CNS-PFS. This finding was corroborated in the brain metastasis subgroups of FLAURA, LASER301, and FURLONG 21,23,25. Furthermore, we observed that TGET demonstrated superior DOR and depth of response for measurable intracranial lesions. The enhanced control of CNS lesions with third-generation drugs is primarily attributed to their improved blood-brain barrier permeability, allowing TGETs to achieve higher CNS concentration and brain-to-plasma concentration ratios 31,32.

Safety is another crucial consideration in drug selection, as drugs with good efficacy but significant side effects are common in clinical practice. Our study revealed that the top 5 AEs in the TGET group were diarrhea (36.32%), rash (30.24%), decreased platelet count (29.15%), elevated serum creatinine (23.63%), and decreased white blood cell count (22.02%). The incidence of 26 AEs, including decreased platelet count, was higher in the TGET group. Among them, the decline in blood cell counts (red blood cells, white blood cells, and platelets) was the most pronounced difference compared to FGET. Additionally, more serious AEs and more discontinuations due to AEs were also observed in the TGET group. Among these six TGETs, the incidence rates of grade 3-5 AEs ranked from highest to lowest were Naquotinib (54.31%), Befotertinib (47.25%), Osimertinib (41.94%), Lazertinib (40.82%), Aumolertinib (36.45%), and Furmonertinib (34.83%) 6-11. The ability of third-generation EGFR-TKIs to control intracranial lesions in EGFR-mutated advanced NSCLC patients is accompanied by significant CNS toxicities such as headaches, dizziness, and cognitive impairments due to enhanced drug penetration across the blood-brain barrier 38. Additionally, patients may experience radiation necrosis or leukoencephalopathy, particularly if they have undergone prior radiotherapy, necessitating careful monitoring and management 6,39. Resistance mechanisms, such as C797S mutations, can lead to CNS relapse, requiring a combination of systemic therapy and localized treatments 40. Therefore, although TGETs can substantially improve survival, close monitoring and management of AEs still require high attention.

This meta-analysis has several limitations. Firstly, it initially included only English articles, which may introduce language bias. Secondly, the TGET group comprised only six types of TGETs, potentially excluding other varieties. Thirdly, the data for analysis were solely derived from previous publications, leading to data heterogeneity. Fourthly, the absence of individual patient data hindered an individual patient data meta-analysis, possibly reducing the clinical value. Fifthly, the differences in median follow-up times among studies could contribute to data heterogeneity. Lastly, the majority of study populations were Asian (84% in each group), raising uncertainties about the generalizability to other populations.

## Conclusion

TGETs appear to outperform FGETs in EGFR-mutated locally advanced or metastatic NSCLC, demonstrating superior survival and responses. This superiority, particularly evident in CNS-PFS, is consistent across most subgroups, except for the TGET-Naquotinib subgroup. However, the TGET group exhibits a high incidence of AEs, particularly hematologic AEs, which necessitates careful consideration. Validation of these results in large-scale RCTs is necessary due to the aforementioned limitations.

## Supplementary Material

Supplementary figures and tables.

## Figures and Tables

**Table 1:** Characteristics of the included studies.

Study	Country	Groups	Patients	Sex (M/F)	Age (Mean, year)	Smoking (Yes/No)	ECOG PS (0/1/2)	Histologic type (Adeno/Others)	Stage (IIIB/IV)	CNS metastases (Yes/No)	EGFR mutation (Ex19del/L858R/T790M)	Follow up (months)	

**NCT02296125(FLAURA, 2014.12-2016.03)**													
Cheng 2021 17	Global multicenter-China Subset	Osimertinib	71	28/43	60	18/53	7/64/0	70/1	2/69	17/54	36/35/0	35.8	
		Gef	65	19/46	61	15/50	13/52/0	64/1	0/65	21/44	33/32/0	27	
Ramalingam 2020 18	Global multicenter	Osimertinib	279	101/178	64	97/182	112/167/0	275/4	14/265	53/226	175/104/0	35.8	
		Gef/Erl	277	195/172	64	102/175	116/161/0	272/0	15/262	63/214	174/103/0	27	
Ohe 2019 19	Global multicenter-Japen Subset	Osimertinib	65	22/43	67	30/65	38/27/0	65/0	5/60	14/51	33/32/0	15	
		Gef	55	27/28	67	26/29	34/21/0	55/0	2/53	13/42	30/25/0	9.7	
Cho 2019 20	Global multicenter-Asian Subset	Osimertinib	162	61/101	64	58/104	65/97/0	162/0	8/154	39/123	-	15	
		Gef/Erl	160	69/91	64	65/95	62/98/0	160/0	4/156	33/127	-	9.7	
Ohe 2018 6	Global multicenter	Osimertinib	279	101/178	64	97/182	112/167/0	275/4	14/265	53/226	175/104/0	15	
		Gef/Erl	277	195/172	64	102/175	116/161/0	272/0	15/262	63/214	174/103/0	9.7	
Reungwetwattana 2018 21	Global multicenter-CNS Subset	Osimertinib	61	23/38	63	-	16/45/0	61/0	0/61	61/0	40/21/0	15	
		Gef/Erl	67	26/41	63	-	27/39/0	67/0	0/67	67/0	45/22/0	9.7	
**NCT04248829(LASER301, 2020.02-2021.09)**													
Lee 2024 22	Global multicenter-Korean Subset	Lazertinib	87	36/51	67	32/55	18/69/0	87/0	2/85	31/56	50/37/0	23.3	
		Gef	85	42/43	66	26/59	20/65/0	85/0	1/84	25/60	48/37/0	26.1	
Soo 2023 23	Global multicenter-CNS Subset	Lazertinib	45	14/31	66	-	8/37/0	45/0	0/45	45/0	25/20/0	17.8	
		Gef	41	17/24	59	-	12/29/0	41/0	0/41	41/0	23/18/0	12.2	
Reungwetwattana 2023 24	Global multicenter-Asian Subset	Lazertinib	129	49/80	66	43/86	30/99/0	129/0	3/126	39/90	77/52/0	21	
		Gef	129	57/72	64	34/95	31/98/0	129/0	3/126	31/98	77/52/0	22.1	
Cho 2023 7	Global multicenter	Lazertinib	196	64/132	67	61/135	49/147/0	196/0	5/191	51/145	121/75/0	20.5	
		Gef	197	78/119	64	48/149	53/144/0	197/0	5/192	48/149	122/75/0	20.6	
**NCT03787992(FURLONG, 2019.05-2019.12)**													
Shi 2022 8	China multicenter	Furmonertinib	178	62/116	59	41/137	39/138/1	178/0	10/168	62/115	91/87/0	21	
		Gefitinib	179	68/111	60	44/135	28/151/0	179/0	7/172	58/121	92/87/0	21	
Shi 2022 CNS 25	China multicenter-CNS Subset	Furmonertinib	65	25/40	58	14/51	14/51/0	65/0	0/65	65/0	35/30/0	21	
		Gefitinib	62	23/39	60	18/44	9/53/0	62/0	0/62	62/0	32/30/0	21	
**NCT03849768(AENEAS, 2018.11-2019.09)**													
Lu 2022 9	China multicenter	Aumolertinib	214	80/134	59	58/156	51/160/0	210/4	12/202	56/158	140/74/0	20.5	
		Gefitinib	215	80/135	62	71/144	54/159/0	211/4	17/198	59/156	141/74/0	20.7	
**NCT02588261(SOLAR, 2016.02-2017.12)**													
Kelly 2019 10	Global multicenter	Naquotinib	267	96/171	68	96/171	103/155/9	267/0	14/253	-	134/111/4	3.6	
		Gef/Erl	263	110/153	67	92/171	103/152/8	263/0	16/247	-	129/108/6	3.6	
**NCT04206072(2019.12-2020.12)**													
Lu 2023 11	China multicenter	Befotertinib	182	72/110	60	61/121	35/146/0	182/0	17/165	47/135	117/65/0	20.7	
		Icotinib	180	72/108	58	55/125	39/141/0	180/0	6/174	45/135	117/63/0	19.4	

**Abbreviations:** CNS: Central nervous system; ECOG: Eastern Cooperative Oncology Group; EGFR: Epidermal growth factor receptor; Erl: Erlotinib; Gef: Gefitinib; M/F: Male/Female.

**Table 2:** Summary of adverse events.

Adverse events	Studies involved	TGET		FGET		Risk ratio [95% CI]	P
		Event/total	%	Event/total	%		
Total adverse events	6	1282/1316	97.42%	1289/1311	98.32%	0.99 [0.98, 1.01]	0.48
Grade 3-5 adverse events	6	568/1316	43.16%	519/1311	39.59%	1.10 [0.93, 1.29]	0.28
Serious adverse events	5	310/1134	27.34%	264/1131	23.34%	1.17 [1.02, 1.35]	0.03
Fatal adverse events	5	49/1134	4.32%	40/1131	3.54%	1.22 [0.81, 1.83]	0.34
Discontinuation due to adverse events	6	134/1316	10.18%	116/1311	8.85%	1.20 [0.79, 1.84]	0.39
Dose reduction due to adverse events	6	174/1316	13.22%	100/1311	7.63%	1.58 [0.81, 3.09]	0.18
Dose interruption due to adverse events	6	368/1316	27.96%	348/1311	26.54%	1.04 [0.84, 1.29]	0.7
Treatment-related adverse events	7	1073/1173	91.47%	1057/1154	91.59%	1.01 [0.96, 1.06]	0.78
Grade 3-5 treatment-related adverse events	6	272/959	28.36%	192/939	20.45%	1.24 [0.73, 2.12]	0.43
Serious treatment-related adverse events	6	96/991	9.69%	75/974	7.70%	1.15 [0.62, 2.12]	0.67
Fatal treatment-related adverse events	5	5/777	0.64%	2/759	0.26%	2.10 [0.47, 9.32]	0.33

**Abbreviations:** CI: confidence interval; FGET: First generation EGFR tyrosine kinase inhibitor; P: Probability; TGET: Third generation EGFR tyrosine kinase inhibitor.

**Table 3:** Total adverse events with an incidence of greater than 10% according to the TGET groups.

Adverse events	Studies involved	TGET		FGET		Risk ratio [95% CI]	P
		Event/total	%	Event/total	%		
Diarrhea	6	478/1316	36.32%	537/1311	40.96%	0.79 [0.60, 1.04]	0.09
Rash	6	398/1316	30.24%	699/1311	53.32%	0.54 [0.34, 0.86]	0.01
Platelet count decreased	4	188/645	29.15%	35/639	5.48%	5.08 [1.72, 14.96]	0.003
Elevated serum creatinine	1	43/182	23.63%	8/180	4.44%	5.32 [2.57, 10.99]	<0.00001
White blood cell count decreased	4	142/645	22.02%	81/639	12.68%	1.77 [1.19, 2.64]	0.005
Peripheral sensory neuropathy	3	141/645	21.86%	22/640	3.44%	5.61 [1.38, 22.81]	0.02
Dry skin	3	161/742	21.70%	190/737	25.78%	0.80 [0.42, 1.54]	0.51
Deep vein thrombosis	1	39/182	21.43%	1/180	0.56%	38.57 [5.36, 277.75]	0.0003
ALT increased	6	272/1316	20.67%	479/1311	36.54%	0.56 [0.41, 0.78]	0.0005
Hyponatremia	2	69/338	20.41%	4/328	1.22%	15.08 [5.88, 38.64]	<0.00001
Lipid metabolism diseases	1	36/182	19.78%	17/180	9.44%	2.09 [1.22, 3.59]	0.007
Paronychia	3	146/742	19.68%	195/737	26.46%	0.45 [0.14, 1.47]	0.18
Urinary tract infection	4	124/645	19.22%	106/639	16.59%	1.16 [0.92, 1.47]	0.21
Cough	3	119/639	18.62%	100/636	15.72%	1.18 [0.93, 1.51]	0.17
AST increased	6	244/1316	18.54%	437/1311	33.33%	0.56 [0.45, 0.70]	<0.00001
Renal symptoms	1	50/279	17.92%	32/277	11.55%	1.55 [1.03, 2.34]	0.04
Hyperuricaemia	1	32/182	17.58%	14/180	7.78%	2.26 [1.25, 4.09]	0.007
Anemia	5	184/1049	17.54%	95/1048	9.06%	1.90 [1.26, 2.87]	0.002
Upper respiratory tract infection	4	149/853	17.47%	106/851	12.46%	1.40 [1.12, 1.77]	0.004
Weight increased	2	62/360	17.22%	66/359	18.38%	0.94 [0.68, 1.28]	0.68
Increased blood creatine phosphokinase	3	94/574	16.38%	33/574	5.75%	2.12 [0.79, 5.69]	0.14
Headache	4	137/853	16.06%	51/851	5.99%	2.58 [1.14, 5.80]	0.02
Fatigue	3	113/728	15.52%	65/720	9.03%	1.72 [1.29, 2.29]	0.0002
Stomatitis	4	139/920	15.11%	143/916	15.61%	0.85 [0.39, 1.88]	0.69
Decreased appetite	5	170/1138	14.94%	147/1132	12.99%	1.16 [0.80, 1.68]	0.45
Proteinuria	2	37/253	14.62%	22/245	8.98%	1.63 [0.99, 2.68]	0.05
Constipation	4	120/835	14.37%	84/833	10.08%	1.42 [1.10, 1.85]	0.008
Elevated fibrin D-dimer	1	26/182	14.29%	8/180	4.44%	3.21 [1.50, 6.91]	0.003
Neutrophil count decreased	3	65/463	14.04%	35/459	7.63%	1.84 [1.24, 2.72]	0.002
Nausea	6	183/1316	13.91%	134/1311	10.22%	1.41 [0.91, 2.18]	0.12
Pruritus	5	140/1049	13.35%	125/1048	11.93%	0.98 [0.62, 1.55]	0.94
Musculoskeletal pain	3	85/639	13.30%	22/636	3.46%	2.62 [0.49, 14.06]	0.26
Muscle spasms	1	26/196	13.27%	7/197	3.55%	3.73 [1.66, 8.40]	0.001
Blood lactate dehydrogenase increase	1	26/214	12.15%	14/215	6.51%	1.87 [1.00, 3.47]	0.05
Vomiting	4	97/853	11.37%	62/851	7.29%	1.56 [1.15, 2.11]	0.004
Insomnia	1	31/279	11.11%	21/277	7.58%	1.47 [0.86, 2.49]	0.16
Nasopharyngitis	1	31/279	11.11%	16/277	5.78%	1.92 [1.08, 3.44]	0.03
Dermatitis acneiform	1	21/196	10.71%	27/197	13.71%	0.78 [0.46, 1.33]	0.37
Back pain	2	48/457	10.50%	48/456	10.53%	1.00 [0.68, 1.46]	0.99
Pyrexia	2	48/461	10.41%	31/457	6.78%	1.48 [0.48, 4.62]	0.5
Hematuria	2	26/253	10.28%	37/245	15.10%	0.81 [0.32, 2.03]	0.65
Dyspnea	3	65/639	10.17%	41/636	6.45%	1.58 [1.08, 2.29]	0.02

**Abbreviations:** ALT: Alanine aminotransferase; AST: Aspartate aminotransferase; CI: confidence interval; ECG: Electrocardiogram; FGET: First generation EGFR tyrosine kinase inhibitor; P: Probability; TGET: Third generation EGFR tyrosine kinase inhibitor.

**Table 4:** Grade 3-5 adverse events with an incidence of greater than 1% according to the TGET groups.

Adverse events	Studies involved	TGET		FGET		Risk ratio [95% CI]	P
		Event/total	%	Event/total	%		
Hyponatremia	2	63/546	11.54%	6/540	1.11%	7.50 [0.54, 105.21]	0.13
Diarrhea	6	34/1249	2.72%	17/1243	1.37%	1.79 [0.89, 3.60]	0.02
Platelet count decreased	4	23/853	2.70%	3/851	0.35%	3.77 [0.85, 16.76]	0.0009
Increased blood creatine phosphokinase	3	15/574	2.61%	2/574	0.35%	2.76 [0.07, 115.54]	0.59
Pulmonary embolism	3	16/639	2.50%	2/636	0.31%	5.89 [1.53, 22.71]	0.005
ALT increased	6	31/1249	2.48%	99/1243	7.96%	0.25 [0.09, 0.68]	0.007
Lipid metabolism diseases	1	4/182	2.20%	1/180	0.56%	3.96 [0.45, 35.05]	0.22
Hypertension	4	18/853	2.11%	12/851	1.41%	1.59 [0.43, 5.87]	0.49
Anemia	5	20/982	2.04%	11/980	1.12%	1.63 [0.72, 3.66]	0.12
Pneumonia	4	17/853	1.99%	18/851	2.12%	0.98 [0.45, 2.14]	0.86
Renal symptoms	1	5/279	1.79%	1/277	0.36%	4.96 [0.58, 42.22]	0.14
Ejection fraction decrease	1	5/279	1.79%	1/277	0.36%	4.96 [0.58, 42.22]	0.14
Fatigue	3	12/728	1.65%	3/720	0.42%	3.38 [0.56, 20.26]	0.03
Decreased appetite	5	16/1071	1.49%	9/1064	0.85%	1.66 [0.72, 3.83]	0.17
Neutrophil count decreased	3	10/671	1.49%	1/671	0.15%	5.22 [1.14, 23.82]	0.03
Hypokalemia	3	10/671	1.49%	12/671	1.79%	0.84 [0.36, 1.95]	0.67
Pathological fracture	1	4/279	1.43%	2/277	0.72%	0.20 [0.01, 4.12]	0.3
White blood cell count decreased	4	12/853	1.41%	2/851	0.24%	3.77 [1.04, 13.60]	0.02
ECG QTc prolongation	4	12/853	1.41%	10/851	1.18%	1.22 [0.51, 2.88]	0.66
Gamma-glutamyl transferase increase	3	8/671	1.19%	6/671	0.89%	1.42 [0.24, 8.56]	0.6
Lymphocyte count decreased	2	5/457	1.09%	0/456	0.00%	5.45 [0.63, 47.00]	0.1
Deep vein thrombosis	2	5/461	1.08%	1/457	0.22%	2.84 [0.31, 25.75]	0.16
Cataract disorder	1	3/279	1.08%	1/277	0.36%	2.98 [0.31, 28.46]	0.34
Sepsis	1	3/279	1.08%	3/277	1.08%	1.99 [0.37, 10.75]	0.43
Peripheral sensory neuropathy	3	6/578	1.04%	1/572	0.17%	2.86 [0.52, 15.60]	0.12

**Abbreviations:** ALT: Alanine aminotransferase; CI: confidence interval; ECG: Electrocardiogram; FGET: First generation EGFR tyrosine kinase inhibitor; P: Probability; TGET: Third generation EGFR tyrosine kinase inhibitor.

## Abbreviations

AEs: Adverse effects
ALT: Alanine aminotransferase
ALTD: AEs leading to treatment discontinuation
AST: Aspartate aminotransferase
CI: confidence interval
CNS: Central nervous system
CNS-DOR: Duration of central nervous system response
CNS-PFS: Central nervous system-Progression-free survival
CNS-PFSR: CNS-PFS: Central nervous system-Progression-free survival rate
CR: Complete response
DCR: Disease control rate
DOR: Duration of response
ECG: Electrocardiogram
ECOG: Eastern Cooperative Oncology Group
EGFR: Epidermal growth factor receptor
EPR: Estimated percentage remaining in response
Erl: Erlotinib
FGET: First generation EGFR tyrosine kinase inhibitor
Gef: Gefitinib
GRADE: Grading of Recommendations, Assessment, Development, and Evaluation
HR: Hazard ratio
MD: Mean difference
M/F: Male/Female
NSCLC: Non-small cell lung cancer
ORR: Objective response rate
OS: Overall survival
OSR: Overall survival rate
P: Probability
PD: Progressive disease
PFS: Progression-free survival
PFSR: Progression-free survival rate
PICOS: Participants, Intervention, Control, Outcome and Study design
PR: Partial response
PRISMA: Preferred Reporting Items for Systematic Reviews and Meta-Analysis
RCT: Randomized controlled trial
RR: Risk ratio
SD: Stable disease
TGET: Third generation EGFR tyrosine kinase inhibitor
TKI: Tyrosinkinase inhibitor
