# Supplementary material for: Third generation vs first generation EGFR-TKIs in the first line treatment for EGFR-mutated locally advanced or metastatic non-small cell lung cancer: a meta-analysis based on randomized controlled trials
Source: J Cancer. 2025 Jan 1;16(3):735–47. doi: 10.7150/jca.99319 (PMC11705063; doi:10.7150/jca.99319)
Supplement: Supplementary file 1 — Supplementary figures and tables. [file jcav16p0735s1.pdf]

Supplementary data

Figure S1 Cochrane risk assessment.

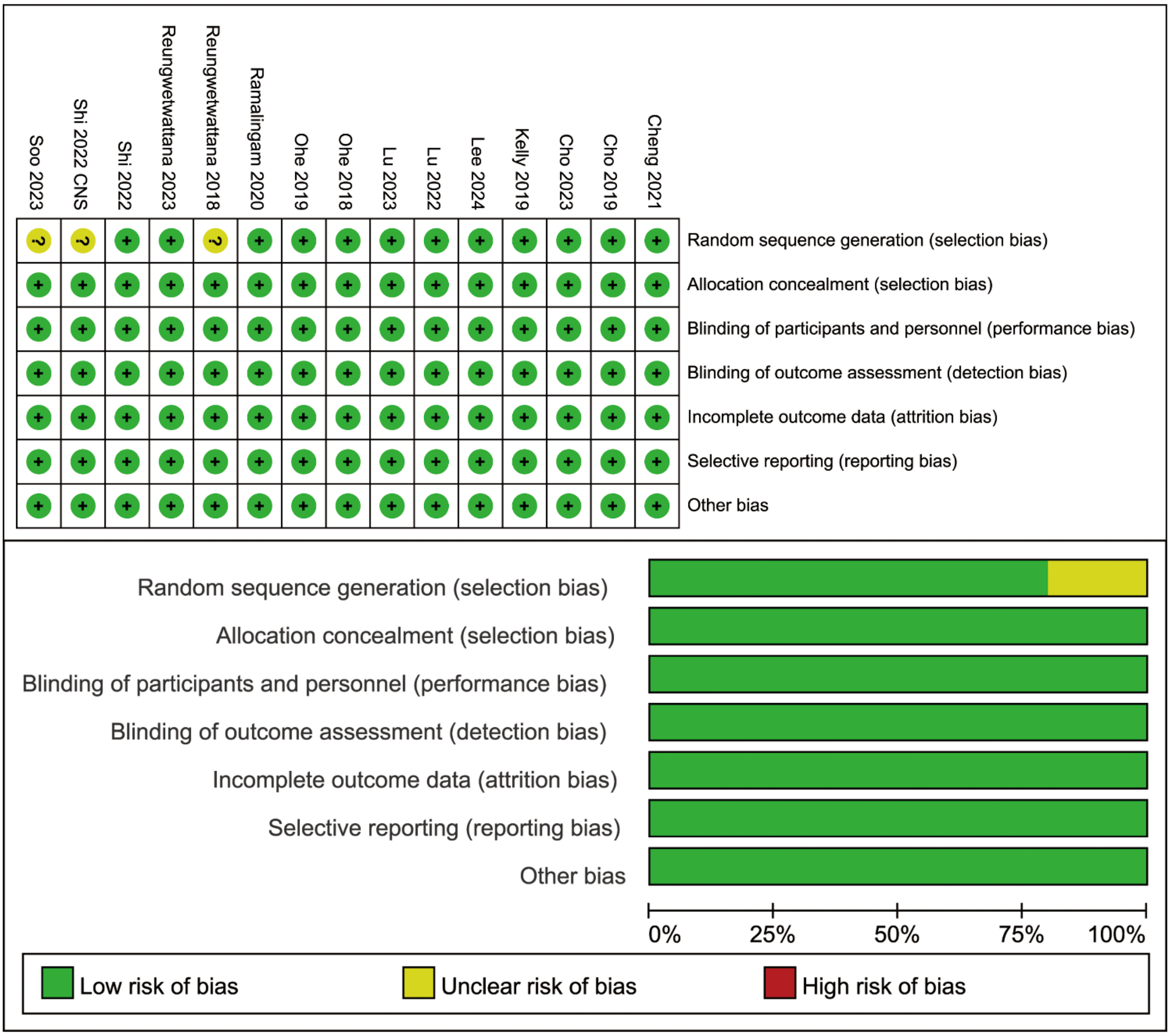

Figure S2 Forest plots of patients' condition at the time of data cutoff associated with TGET versus FGET.

Study or Subgroup	Third generation EGFR TKI Events	Third generation EGFR TKI Total	First generation EGFR TKI Events	First generation EGFR TKI Total	Weight	Risk Ratio M-H, Fixed, 95% CI	Year
**1.1.1 Ongoing**							
Kelly 2019	0	267	0	263		Not estimable	2019
Ramalingam 2020	61	279	13	277	1.2%	4.66 [2.62, 8.28]	2020
Shi 2022	69	178	22	179	2.1%	3.15 [2.05, 4.86]	2022
Lu 2022	93	214	34	215	3.2%	2.75 [1.95, 3.88]	2022
Lu 2023	66	182	29	180	2.7%	2.25 [1.53, 3.31]	2023
Cho 2023	108	196	47	197	4.4%	2.31 [1.75, 3.05]	2023
**Subtotal (95% CI)**		**1316**		**1311**	**13.6%**	**2.74 [2.32, 3.24]**	
Total events	397		145				
Heterogeneity: Chi² = 6.11, df = 4 (P = 0.19); I² = 35%							
Test for overall effect: Z = 11.84 (P < 0.00001)							
**1.1.2 Discontinuation due to progressive disease**						**Modified by random-effects model**	
Kelly 2019	61	267	82	263	6.6%	0.73 [0.55, 0.97]	2019
Ramalingam 2020	153	279	199	277	6.9%	0.76 [0.67, 0.87]	2020
Lu 2022	82	178	129	179	6.8%	0.64 [0.53, 0.77]	2022
Shi 2022	105	214	158	215	6.9%	0.67 [0.57, 0.78]	2022
Lu 2023	81	182	138	180	6.8%	0.58 [0.48, 0.70]	2023
Cho 2023	44	196	109	197	6.6%	0.41 [0.30, 0.54]	2023
**Subtotal (95% CI)**		**1316**		**1311**	**40.7%**	**0.63 [0.54, 0.73]**	
Total events	526		815				
Heterogeneity: Tau² = 0.02; Chi² = 18.73, df = 5 (P = 0.002); I² = 73%							
Test for overall effect: Z = 6.00 (P < 0.00001)							
**1.1.3 Discontinuation due to adverse events**						**Modified by random-effects model**	
Kelly 2019	29	267	24	263	5.9%	1.19 [0.71, 1.99]	2019
Ramalingam 2020	41	279	52	277	6.3%	0.78 [0.54, 1.14]	2020
Shi 2022	11	178	3	179	3.2%	3.69 [1.05, 12.99]	2022
Lu 2022	11	214	9	215	4.5%	1.23 [0.52, 2.90]	2022
Cho 2023	19	196	18	197	5.5%	1.06 [0.57, 1.96]	2023
Lu 2023	20	182	2	180	2.8%	9.89 [2.35, 41.70]	2023
**Subtotal (95% CI)**		**1316**		**1311**	**28.2%**	**1.44 [0.85, 2.43]**	
Total events	131		108				
Heterogeneity: Tau² = 0.26; Chi² = 16.54, df = 5 (P = 0.005); I² = 70%							
Test for overall effect: Z = 1.36 (P = 0.17)							

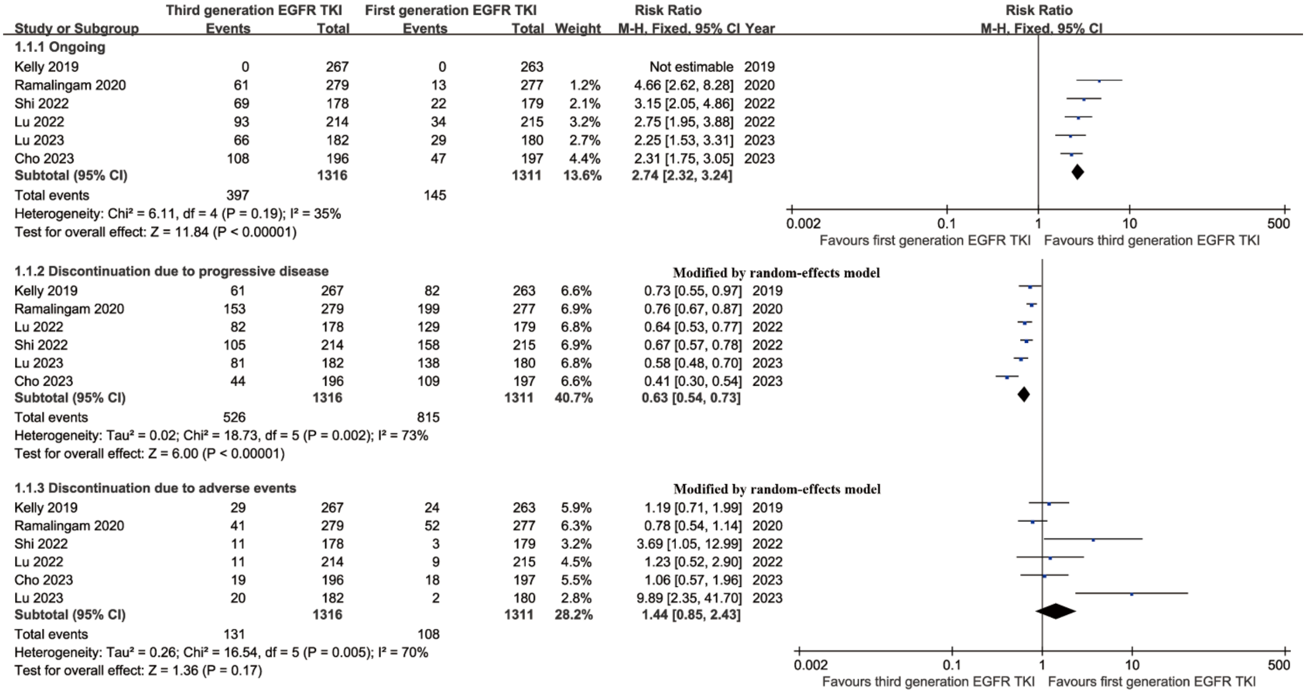

Figure S3 Comparisons of PFSR (6-24 months) associated with TGET versus FGET according to survival time.

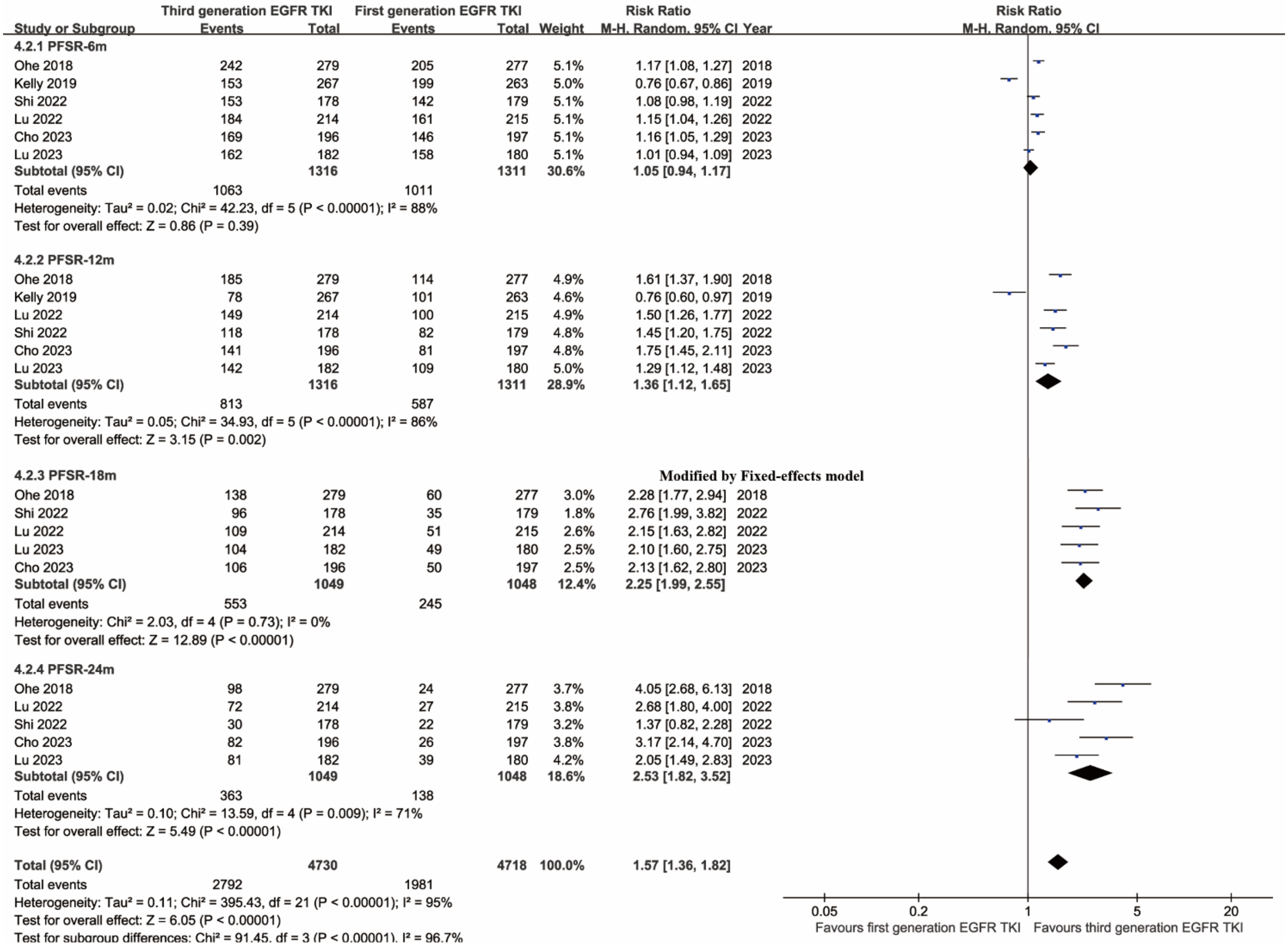

Figure S4 Comparisons of OSR (6-36 months) associated with TGET versus FGET according to survival time.

Study or Subgroup	Third generation EGFR TKI Events	Third generation EGFR TKI Total	First generation EGFR TKI Events	First generation EGFR TKI Total	Weight	Risk Ratio M-H, Fixed, 95% CI	Year
**4.1.1 OSR-6m**							
Ramalingam 2020	272	279	257	277	8.1%	1.05 [1.01, 1.09]	2020
Shi 2022	164	178	164	179	5.2%	1.01 [0.95, 1.07]	2022
Cho 2023	187	196	184	197	5.8%	1.02 [0.97, 1.07]	2023
Lu 2023	170	182	172	180	5.5%	0.98 [0.93, 1.03]	2023
**Subtotal (95% CI)**		**835**		**833**	**24.5%**	**1.02 [0.99, 1.04]**	
Total events	793		777				
Heterogeneity: Chi^2 = 5.41, df = 3 (P = 0.14); I^2 = 45%							
Test for overall effect: Z = 1.46 (P = 0.14)							
**4.1.2 OSR-12m**							
Ramalingam 2020	248	279	227	277	7.2%	1.08 [1.01, 1.16]	2020
Lu 2022	184	214	183	215	5.8%	1.01 [0.93, 1.09]	2022
Shi 2022	149	178	154	179	4.8%	0.97 [0.89, 1.06]	2022
Cho 2023	175	196	165	197	5.2%	1.07 [0.99, 1.15]	2023
Lu 2023	155	182	159	180	5.0%	0.96 [0.89, 1.05]	2023
**Subtotal (95% CI)**		**1049**		**1048**	**28.0%**	**1.02 [0.99, 1.06]**	
Total events	911		888				
Heterogeneity: Chi^2 = 7.25, df = 4 (P = 0.12); I^2 = 45%							
Test for overall effect: Z = 1.38 (P = 0.17)							
**4.1.3 OSR-18m**						**Modified by random-effects model**	
Ramalingam 2020	228	279	198	277	5.3%	1.14 [1.04, 1.25]	2020
Shi 2022	134	178	134	179	4.2%	1.01 [0.89, 1.13]	2022
Lu 2023	137	182	145	180	4.5%	0.93 [0.84, 1.04]	2023
Cho 2023	157	196	141	197	4.4%	1.12 [1.00, 1.25]	2023
**Subtotal (95% CI)**		**835**		**833**	**18.4%**	**1.05 [0.95, 1.15]**	
Total events	656		618				
Heterogeneity: Tau^2 = 0.01; Chi^2 = 9.24, df = 3 (P = 0.03); I^2 = 68%							
Test for overall effect: Z = 1.01 (P = 0.31)							
**4.1.4 OSR-24m**						**Modified by random-effects model**	
Ramalingam 2020	207	279	163	277	4.1%	1.26 [1.12, 1.42]	2020
Shi 2022	122	178	109	179	3.1%	1.13 [0.96, 1.31]	2022
Lu 2023	121	182	126	180	3.5%	0.95 [0.83, 1.09]	2023
Cho 2023	129	196	114	197	3.1%	1.14 [0.97, 1.33]	2023
**Subtotal (95% CI)**		**835**		**833**	**13.8%**	**1.12 [0.99, 1.26]**	
Total events	579		512				
Heterogeneity: Tau^2 = 0.01; Chi^2 = 9.04, df = 3 (P = 0.03); I^2 = 67%							
Test for overall effect: Z = 1.74 (P = 0.08)							
**4.1.5 OSR-30m**							
Ramalingam 2020	178	279	144	277	4.6%	1.23 [1.06, 1.42]	2020
Lu 2023	121	182	108	180	3.4%	1.11 [0.95, 1.30]	2023
**Subtotal (95% CI)**		**461**		**457**	**8.0%**	**1.18 [1.06, 1.31]**	
Total events	299		252				
Heterogeneity: Chi^2 = 0.89, df = 1 (P = 0.35); I^2 = 0%							
Test for overall effect: Z = 2.99 (P = 0.003)							
**4.1.6 OSR-36m**							
Ramalingam 2020	152	279	122	277	3.9%	1.24 [1.04, 1.47]	2020
**Subtotal (95% CI)**		**279**		**277**	**3.9%**	**1.24 [1.04, 1.47]**	
Total events	152		122				
Heterogeneity: Not applicable							
Test for overall effect: Z = 2.44 (P = 0.01)							
**Total (95% CI)**		**4294**		**4281**	**100.0%**	**1.07 [1.04, 1.09]**	
Total events	3390		3169				
Heterogeneity: Chi^2 = 58.70, df = 19 (P < 0.00001); I^2 = 68%							
Test for overall effect: Z = 5.67 (P < 0.00001)							
Test for subgroup differences: Chi^2 = 18.24, df = 5 (P = 0.003), I^2 = 72.6%							

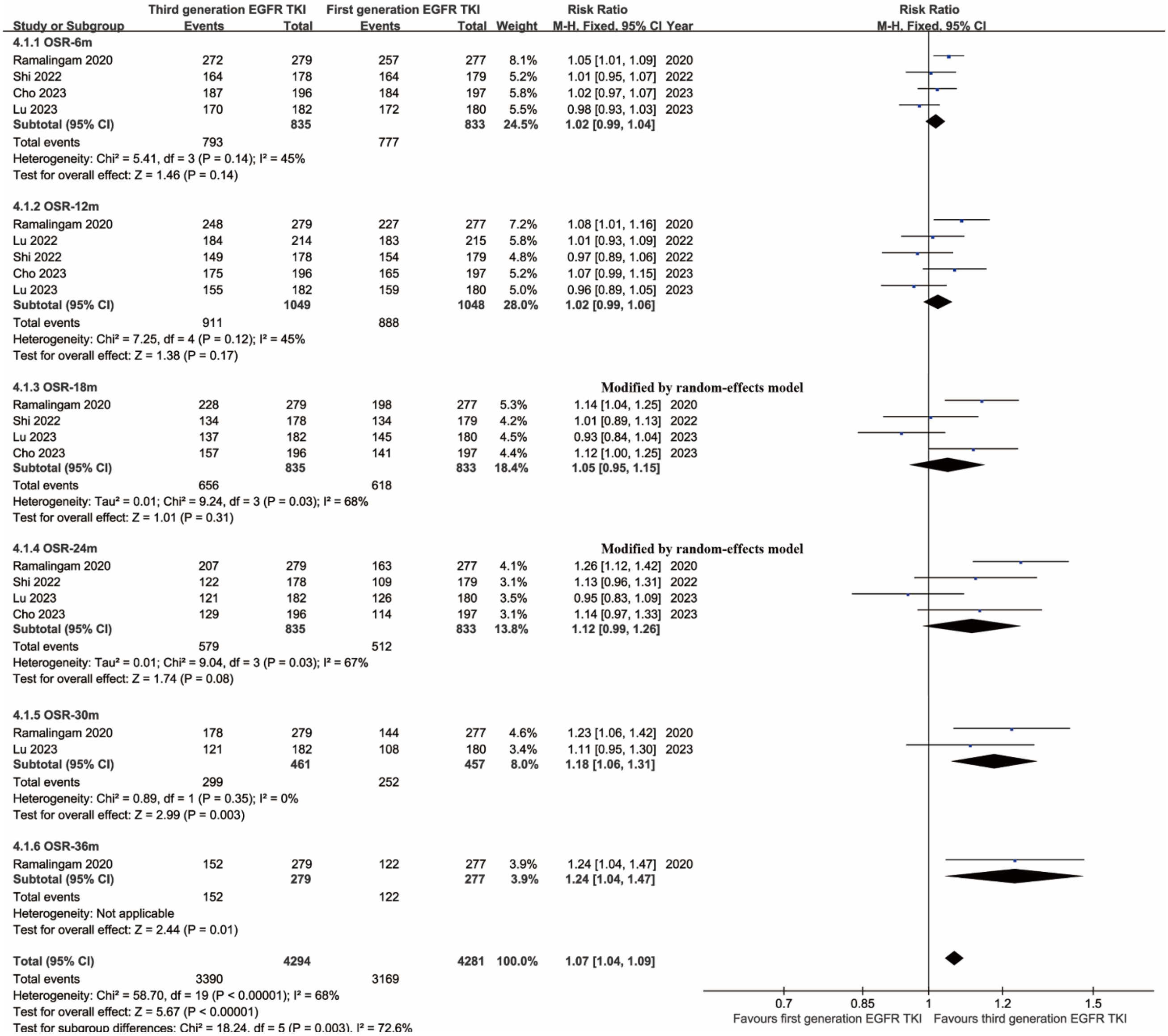

Figure S5 Comparisons of CNS-PFSR (6-24 months) associated with TGET versus FGET according to survival time.

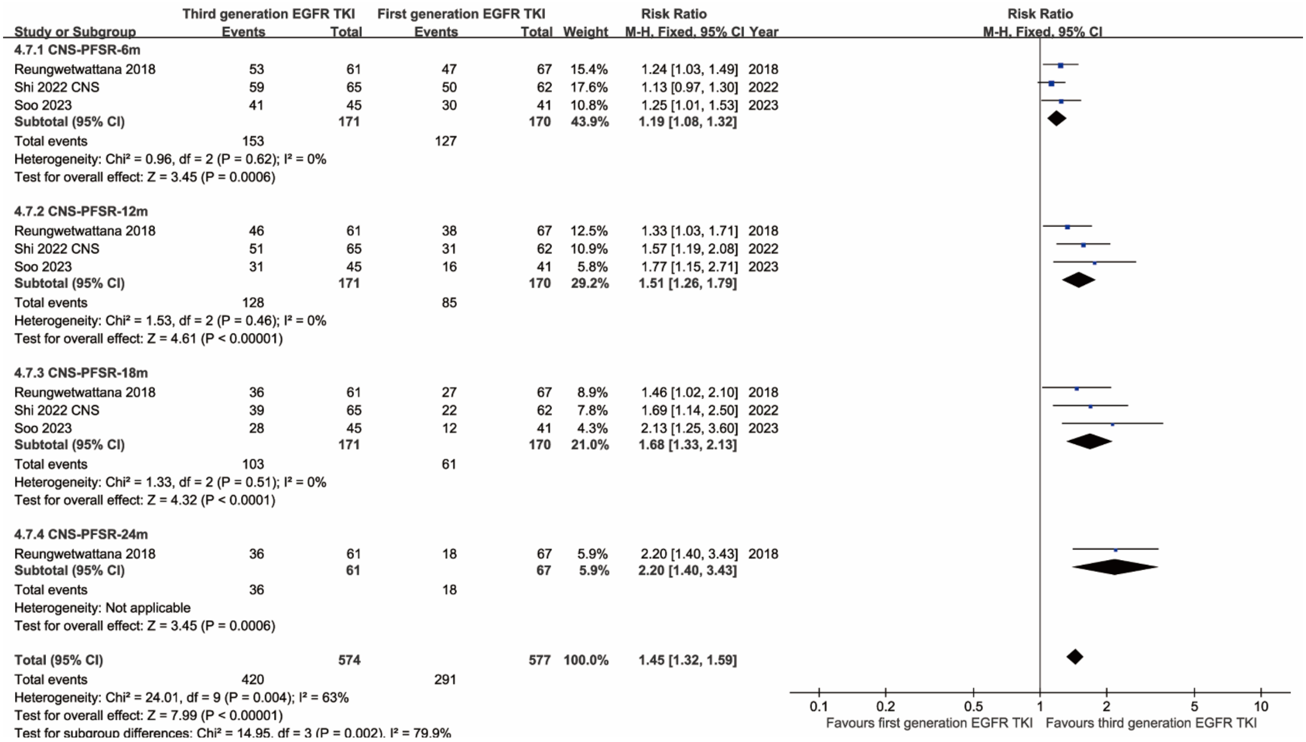

Figure S6 Subgroup analysis of PFSR (6-24 months) in patients with CNS metastases (A), without CNS metastases (B), with Ex19del mutations (C), and with L858R mutations (D).

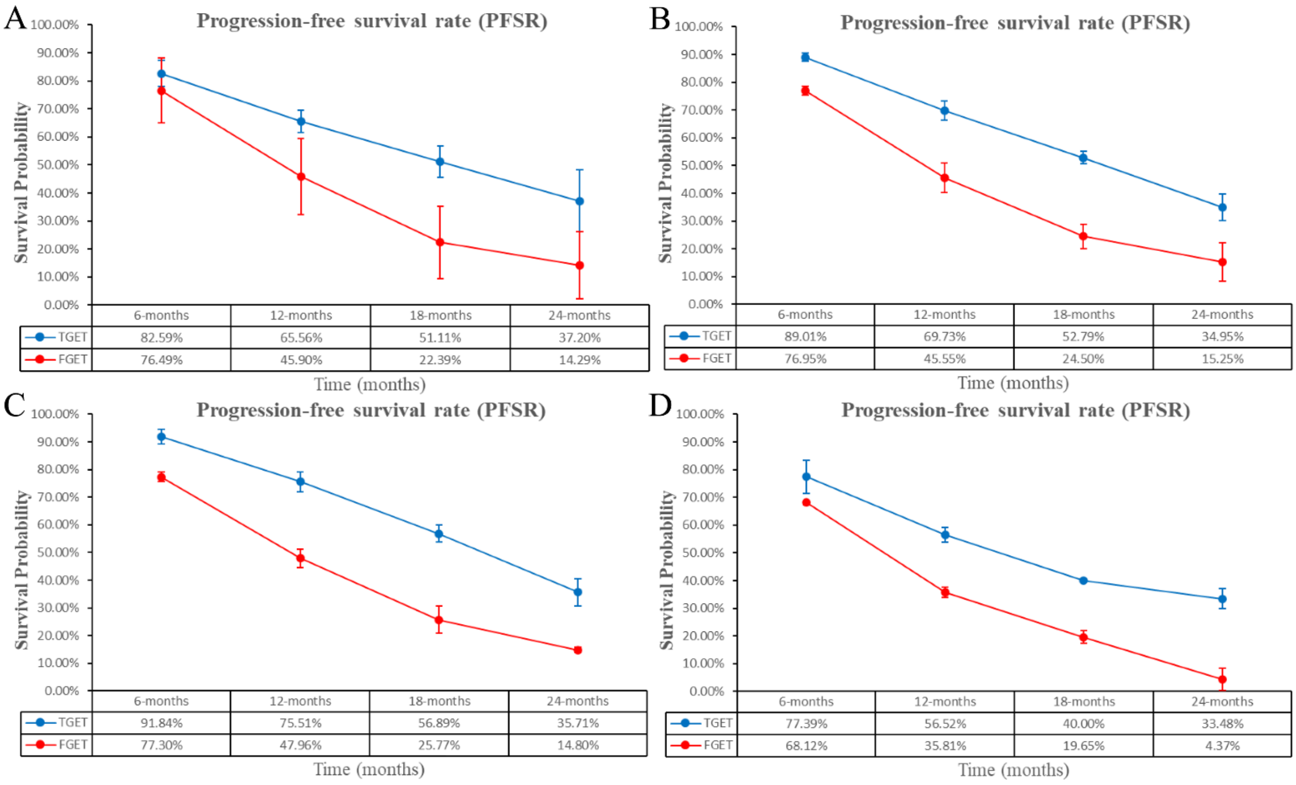

Figure S7 Comparisons of PFSR (6-24 months) associated with TGET versus FGET in patients with CNS metastases according to survival time.

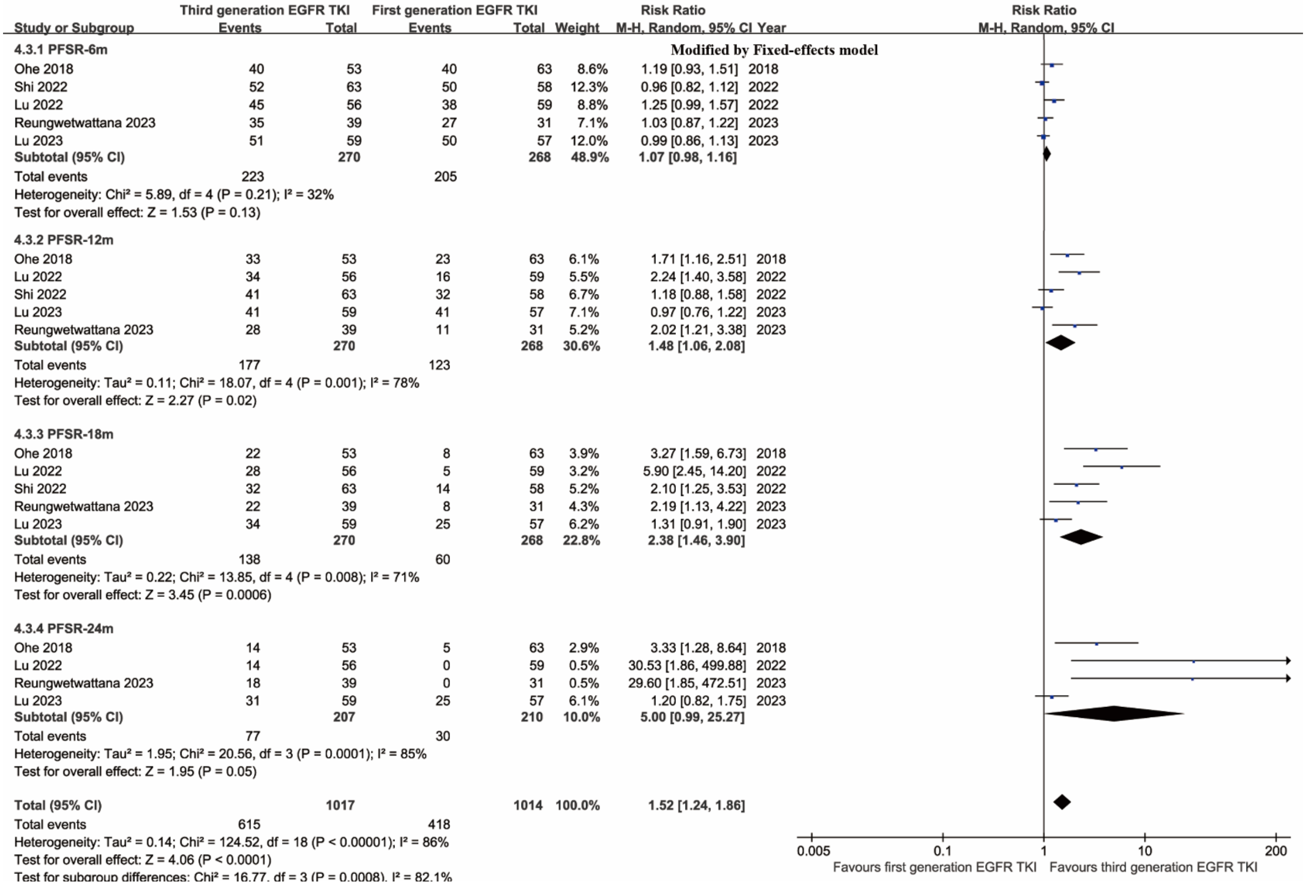

Figure S8 Comparisons of PFSR (6-24 months) associated with TGET versus FGET in patients without CNS metastases according to survival time.

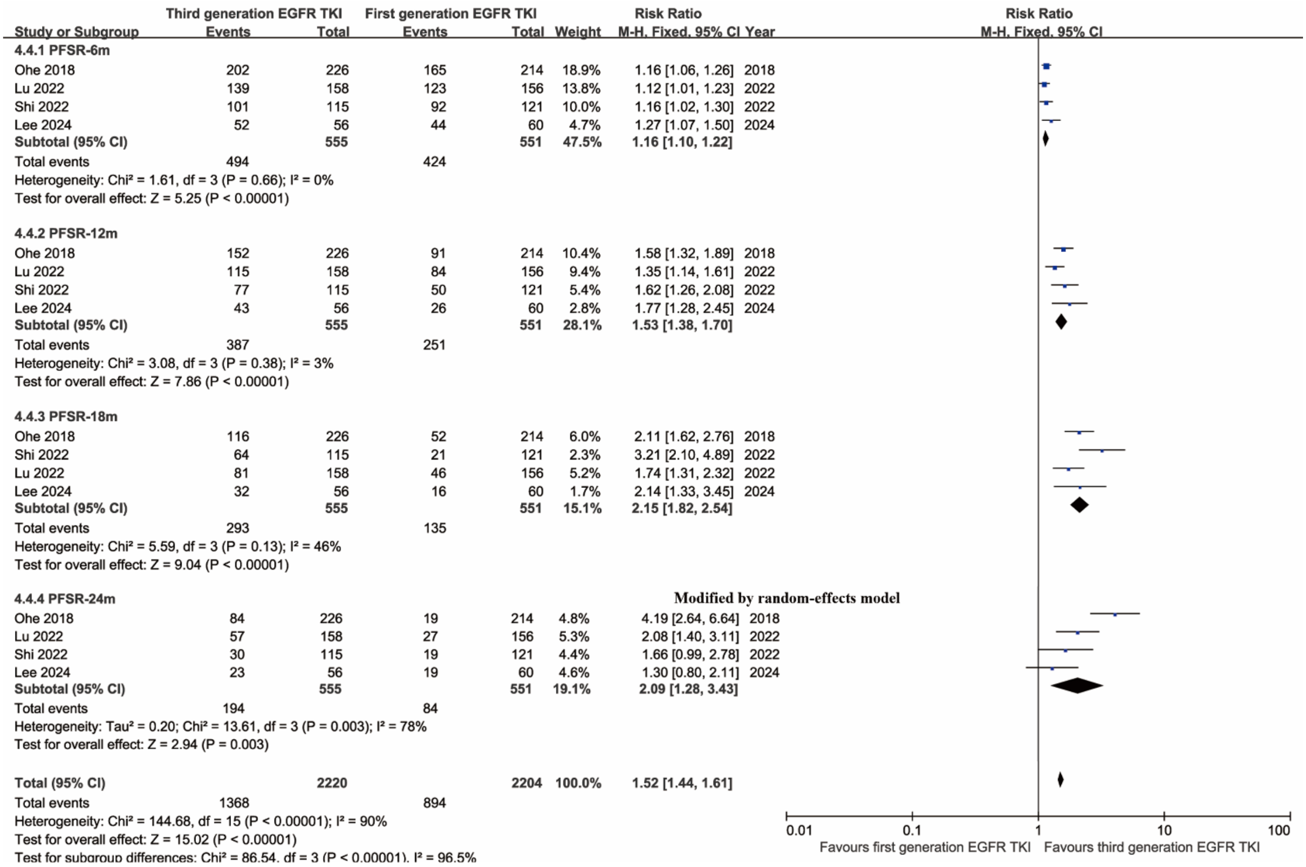

Figure S9 Comparisons of PFSR (6-24 months) associated with TGET versus FGET in patients with Ex19del mutations according to survival time.

Study or Subgroup	Third generation EGFR TKI Events	Third generation EGFR TKI Total	First generation EGFR TKI Events	First generation EGFR TKI Total	Weight	Risk Ratio M-H, Fixed, 95% CI	Year
**4.5.1 PFSR-6m**							
Ohe 2018	156	175	135	174	20.8%	1.15 [1.04, 1.26]	2018
Lu 2022	133	140	111	141	17.0%	1.21 [1.10, 1.33]	2022
Reungwetwattana 2023	71	77	57	77	8.8%	1.25 [1.07, 1.44]	2023
**Subtotal (95% CI)**		**392**		**392**	**46.6%**	**1.19 [1.12, 1.26]**	
Total events	360		303				
Heterogeneity: Chi² = 0.98, df = 2 (P = 0.61); I² = 0%							
Test for overall effect: Z = 5.52 (P < 0.00001)							
**4.5.2 PFSR-12m**							
Ohe 2018	125	175	77	174	11.9%	1.61 [1.33, 1.95]	2018
Lu 2022	110	140	72	141	11.0%	1.54 [1.28, 1.85]	2022
Reungwetwattana 2023	61	77	39	77	6.0%	1.56 [1.22, 2.01]	2023
**Subtotal (95% CI)**		**392**		**392**	**28.9%**	**1.57 [1.40, 1.77]**	
Total events	296		188				
Heterogeneity: Chi² = 0.13, df = 2 (P = 0.94); I² = 0%							
Test for overall effect: Z = 7.59 (P < 0.00001)							
**4.5.3 PFSR-18m**							
Ohe 2018	95	175	38	174	5.9%	2.49 [1.82, 3.40]	2018
Lu 2022	80	140	36	141	5.5%	2.24 [1.63, 3.07]	2022
Reungwetwattana 2023	48	77	27	77	4.2%	1.78 [1.25, 2.52]	2023
**Subtotal (95% CI)**		**392**		**392**	**15.5%**	**2.21 [1.83, 2.67]**	
Total events	223		101				
Heterogeneity: Chi² = 2.03, df = 2 (P = 0.36); I² = 2%							
Test for overall effect: Z = 8.24 (P < 0.00001)							
**4.5.4 PFSR-24m**							
Ohe 2018	67	175	24	174	3.7%	2.78 [1.83, 4.21]	2018
Lu 2022	41	140	22	141	3.4%	1.88 [1.18, 2.98]	2022
Reungwetwattana 2023	32	77	12	77	1.8%	2.67 [1.49, 4.78]	2023
**Subtotal (95% CI)**		**392**		**392**	**8.9%**	**2.41 [1.84, 3.17]**	
Total events	140		58				
Heterogeneity: Chi² = 1.68, df = 2 (P = 0.43); I² = 0%							
Test for overall effect: Z = 6.34 (P < 0.00001)							
**Total (95% CI)**		**1568**		**1568**	**100.0%**	**1.57 [1.47, 1.67]**	
Total events	1019		650				
Heterogeneity: Chi² = 105.13, df = 11 (P < 0.00001); I² = 90%							
Test for overall effect: Z = 14.14 (P < 0.00001)							
Test for subgroup differences: Chi² = 67.04, df = 3 (P < 0.00001), I² = 95.5%							

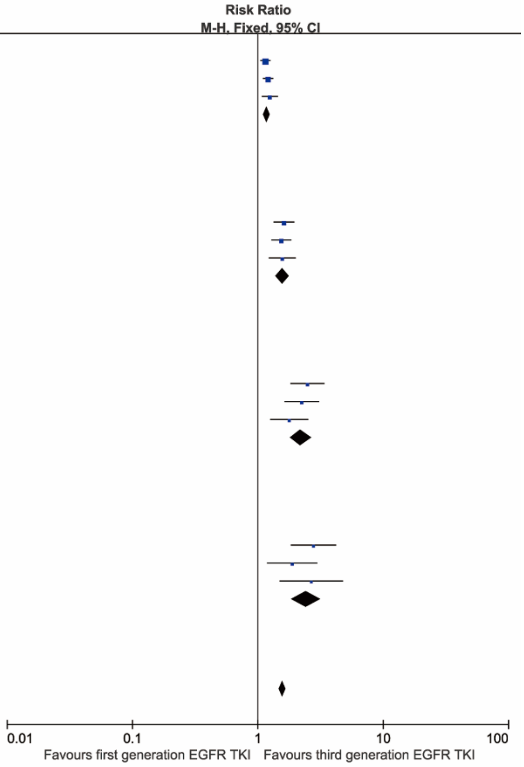

Figure S10 Comparisons of PFSR (6-24 months) associated with TGET versus FGET in patients with L858R mutations according to survival time.

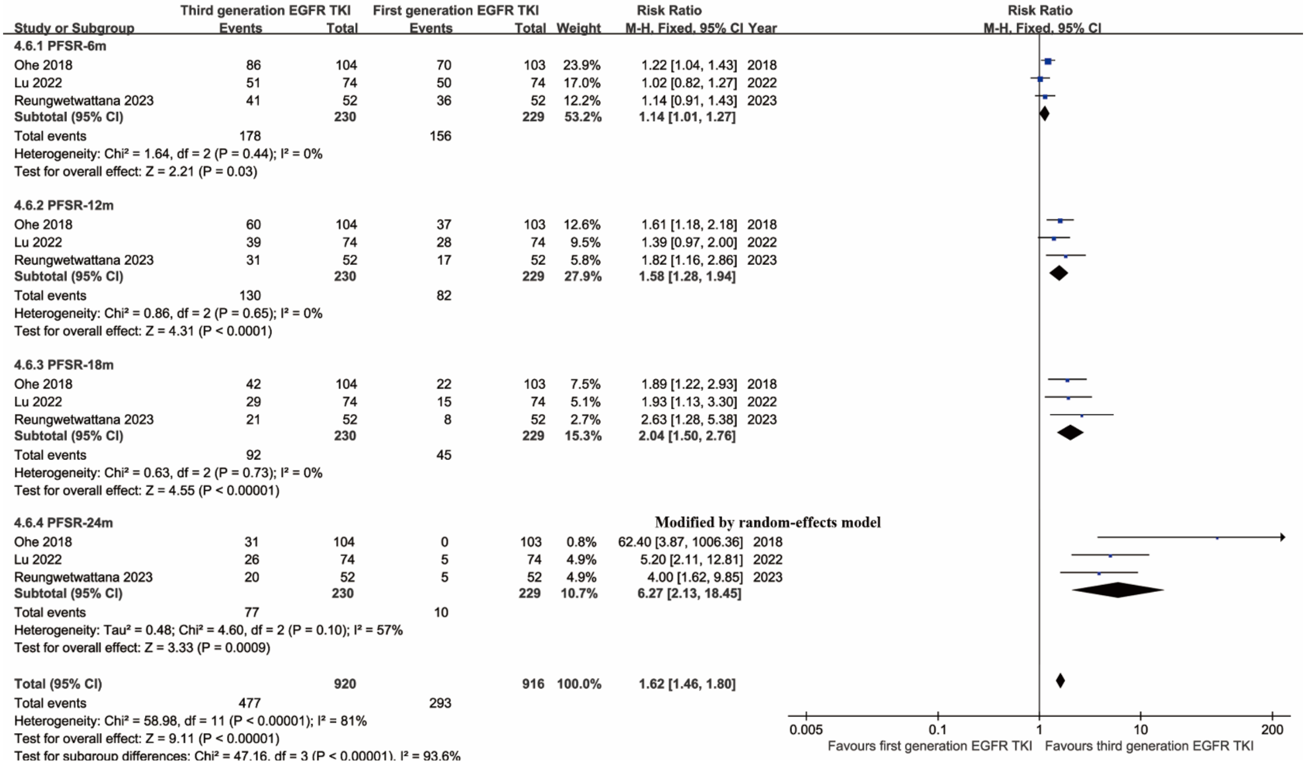

Figure S11 Trend of risk ratios in the subgroup analysis of PFSR (6-24 months) in patients with CNS metastases (A), without CNS metastases (B), with Ex19del mutations (C), and with L858R mutations (D).

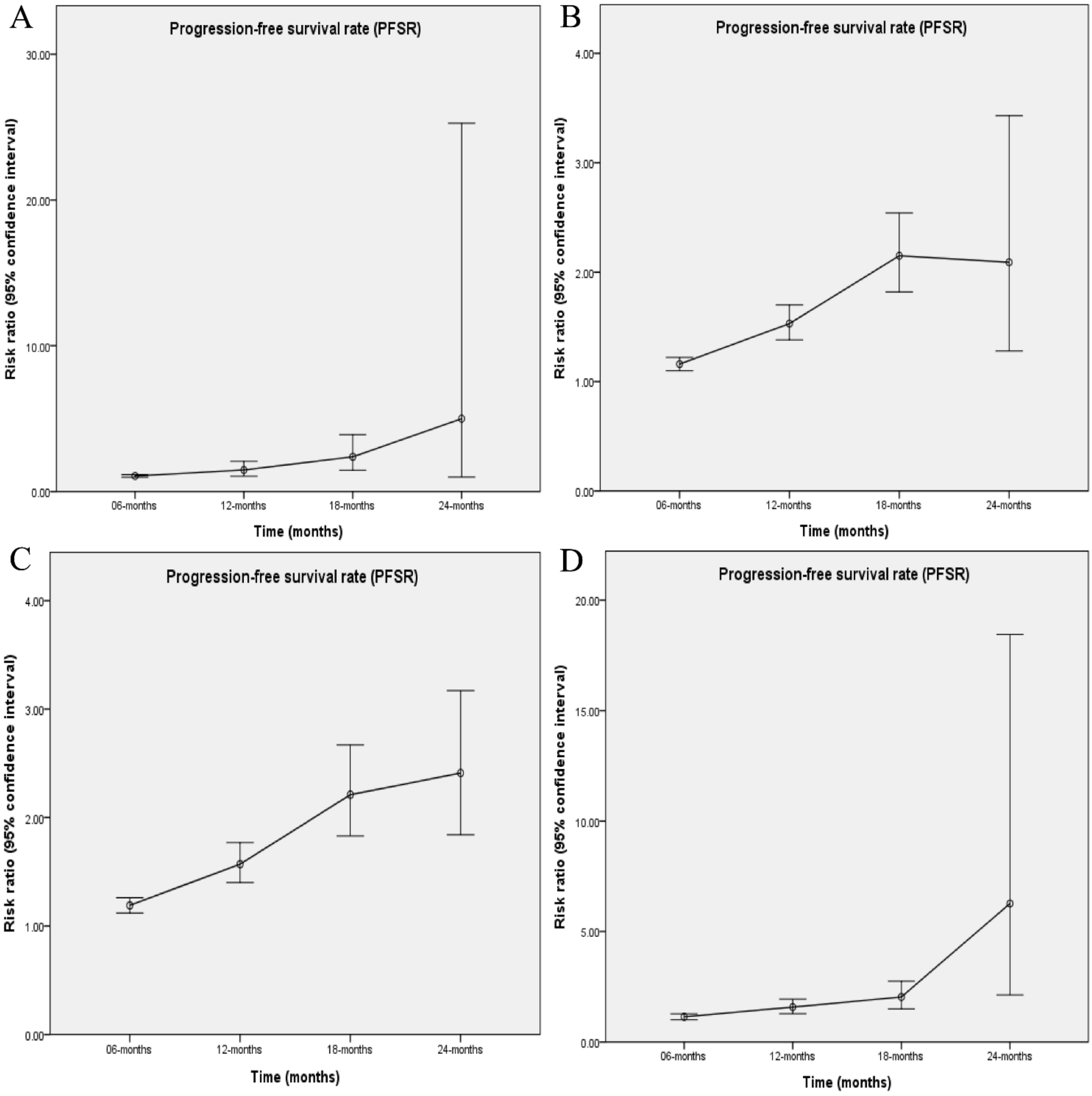

Figure S12 Forest plots of responses (ORR, DCR, CR, PR, SD, and PD) associated with TGET versus FGET.

Study or Subgroup	Third generation EGFR TKI Events	Third generation EGFR TKI Total	First generation EGFR TKI Events	First generation EGFR TKI Total	Weight	Risk Ratio M-H, Random, 95% CI	Year
**5.5.1 ORR**							
Cho 2023	149	196	150	197	4.5%	1.00 [0.89, 1.12]	2023
Lu 2023	138	182	141	180	4.5%	0.97 [0.87, 1.08]	2023
Lu 2022	158	214	155	215	4.4%	1.02 [0.91, 1.15]	2022
Shi 2022	158	178	151	179	5.9%	1.05 [0.97, 1.14]	2022
Kelly 2019	88	267	126	263	1.9%	0.69 [0.56, 0.85]	2019
Ohe 2018	223	279	210	277	5.5%	1.05 [0.96, 1.15]	2018
**Subtotal (95% CI)**		**1316**		**1311**	**26.7%**	**0.98 [0.90, 1.07]**	
Total events	914		933				
Heterogeneity: Tau² = 0.01; Chi² = 17.24, df = 5 (P = 0.004); I² = 71%							
Test for overall effect: Z = 0.40 (P = 0.69)							
**5.5.2 DCR**							
Cho 2023	184	196	185	197	7.5%	1.00 [0.95, 1.05]	2023
Lu 2023	170	182	177	180	7.8%	0.95 [0.91, 0.99]	2023
Shi 2022	170	178	167	179	7.5%	1.02 [0.97, 1.08]	2022
Lu 2022	199	214	208	215	7.8%	0.96 [0.92, 1.00]	2022
Kelly 2019	166	267	174	263	3.9%	0.94 [0.83, 1.07]	2019
Ohe 2018	270	279	256	277	8.0%	1.05 [1.01, 1.09]	2018
**Subtotal (95% CI)**		**1316**		**1311**	**42.4%**	**0.99 [0.96, 1.03]**	
Total events	1159		1167				
Heterogeneity: Tau² = 0.00; Chi² = 15.29, df = 5 (P = 0.009); I² = 67%							
Test for overall effect: Z = 0.43 (P = 0.66)							
**5.5.3 CR**						**Modified by Fixed-effects model**	
Cho 2023	2	196	1	197	0.0%	2.01 [0.18, 21.99]	2023
Lu 2023	0	182	0	180		Not estimable	2023
Shi 2022	0	178	0	179		Not estimable	2022
Lu 2022	1	214	1	215	0.0%	1.00 [0.06, 15.96]	2022
Kelly 2019	0	267	0	263		Not estimable	2019
Ohe 2018	7	279	4	277	0.1%	1.74 [0.51, 5.87]	2018
**Subtotal (95% CI)**		**1316**		**1311**	**0.2%**	**1.66 [0.61, 4.54]**	
Total events	10		6				
Heterogeneity: Chi² = 0.16, df = 2 (P = 0.92); I² = 0%							
Test for overall effect: Z = 0.99 (P = 0.32)							
**5.5.4 PR**							
Cho 2023	147	196	149	197	4.4%	0.99 [0.89, 1.11]	2023
Lu 2023	138	182	141	180	4.5%	0.97 [0.87, 1.08]	2023
Lu 2022	157	214	154	215	4.3%	1.02 [0.91, 1.15]	2022
Shi 2022	158	178	151	179	5.9%	1.05 [0.97, 1.14]	2022
Kelly 2019	88	267	126	263	1.9%	0.69 [0.56, 0.85]	2019
Ohe 2018	216	279	206	277	5.3%	1.04 [0.95, 1.14]	2018
**Subtotal (95% CI)**		**1316**		**1311**	**26.3%**	**0.98 [0.90, 1.06]**	
Total events	904		927				
Heterogeneity: Tau² = 0.01; Chi² = 16.63, df = 5 (P = 0.005); I² = 70%							
Test for overall effect: Z = 0.49 (P = 0.62)							
**5.5.5 SD**							
Lu 2023	32	182	36	180	0.6%	0.88 [0.57, 1.35]	2023
Cho 2023	35	196	35	197	0.6%	1.01 [0.66, 1.54]	2023
Shi 2022	12	178	16	179	0.2%	0.75 [0.37, 1.55]	2022
Lu 2022	41	214	53	215	0.8%	0.78 [0.54, 1.12]	2022
Kelly 2019	78	267	48	263	1.0%	1.60 [1.17, 2.20]	2019
Ohe 2018	47	279	46	277	0.7%	1.01 [0.70, 1.47]	2018
**Subtotal (95% CI)**		**1316**		**1311**	**3.9%**	**1.01 [0.79, 1.29]**	
Total events	245		234				
Heterogeneity: Tau² = 0.05; Chi² = 11.02, df = 5 (P = 0.05); I² = 55%							
Test for overall effect: Z = 0.07 (P = 0.95)							
**5.5.6 PD**							
Cho 2023	6	196	8	197	0.1%	0.75 [0.27, 2.13]	2023
Lu 2023	1	182	1	180	0.0%	0.99 [0.06, 15.69]	2023
Shi 2022	5	178	10	179	0.1%	0.50 [0.18, 1.44]	2022
Lu 2022	12	214	6	215	0.1%	2.01 [0.77, 5.26]	2022
Kelly 2019	17	267	9	263	0.2%	1.86 [0.84, 4.10]	2019
Ohe 2018	3	279	14	277	0.1%	0.21 [0.06, 0.73]	2018
**Subtotal (95% CI)**		**1316**		**1311**	**0.6%**	**0.86 [0.42, 1.77]**	
Total events	44		48				
Heterogeneity: Tau² = 0.46; Chi² = 12.41, df = 5 (P = 0.03); I² = 60%							
Test for overall effect: Z = 0.41 (P = 0.68)							

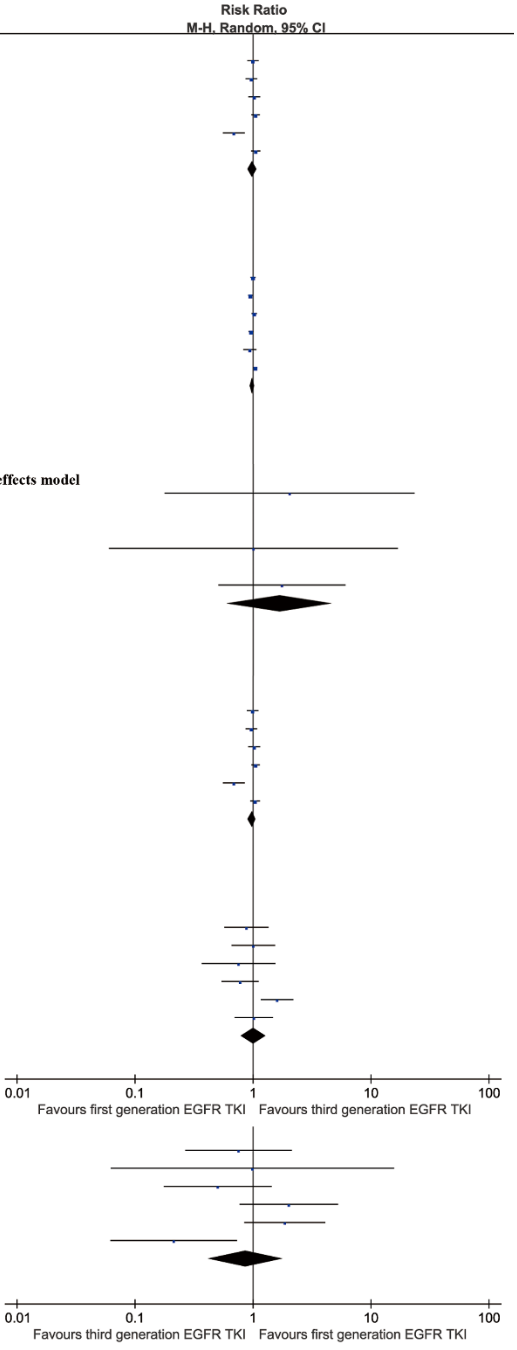

Figure S13 Forest plots of DOR and CNS-DOR associated with TGET versus FGET.

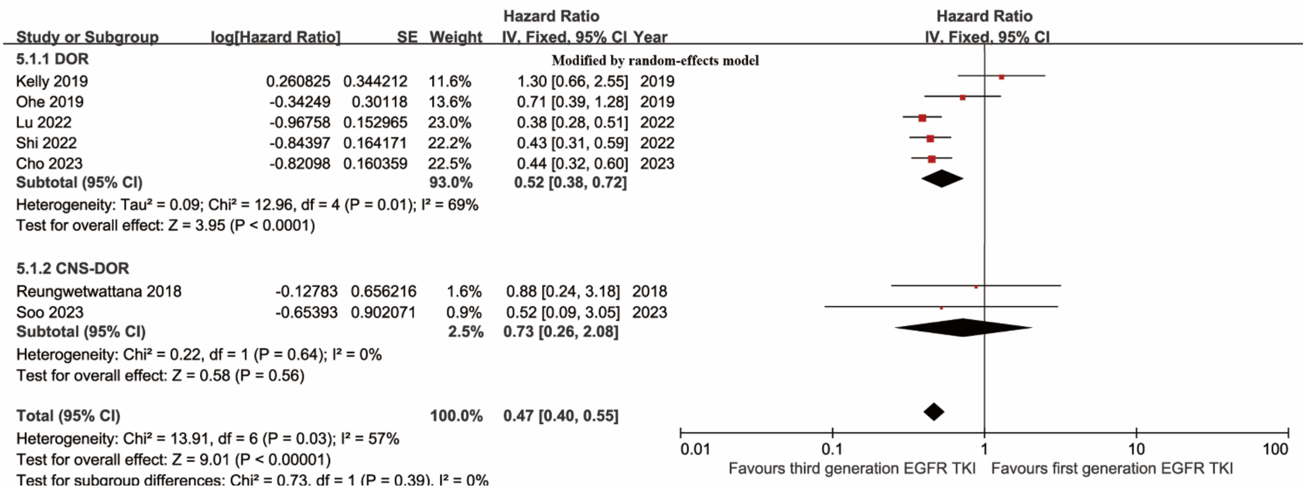

Figure S14 Forest plots of depth of response and depth of CNS response associated with TGET versus FGET.

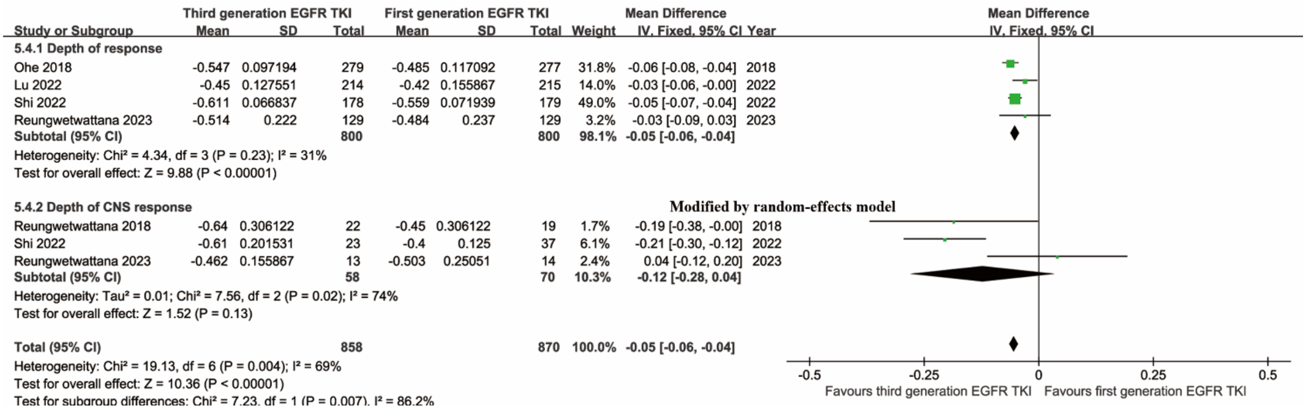

Figure S15 Comparisons of EPR (6-24 months) associated with TGET versus FGET.

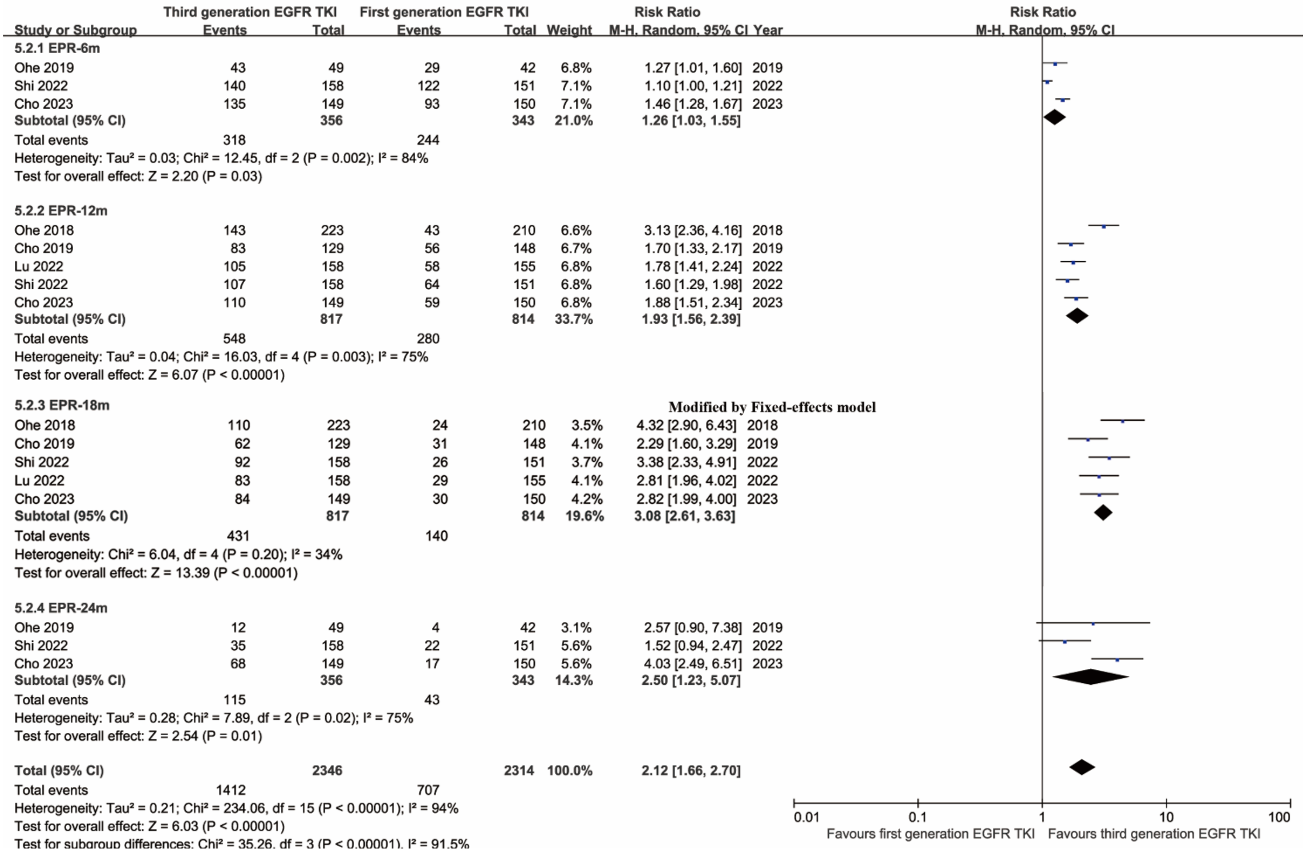

Figure S16 Comparisons of CNS-EPR (6-24 months) associated with TGET versus FGET.

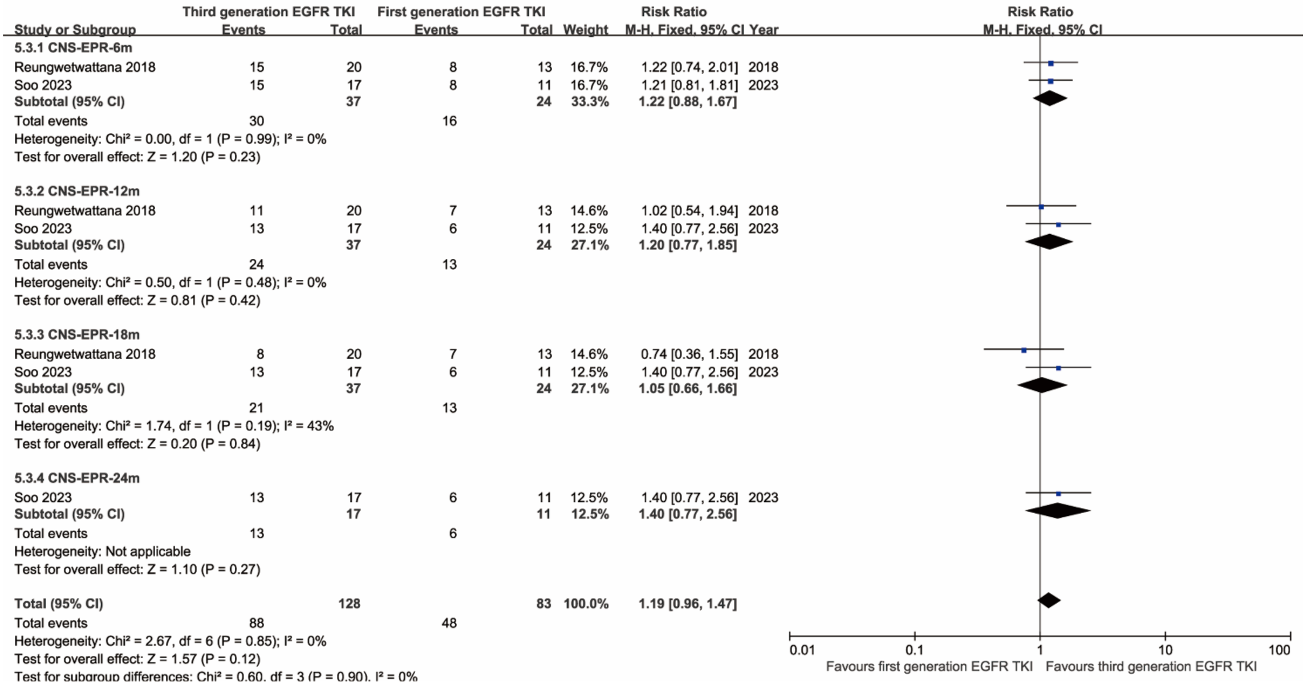

Figure S17 Sensitivity analysis of PFS (A), ORR (B), DCR (C), DOR (D), total AEs (E), and grade 3-5 AEs (F).

A

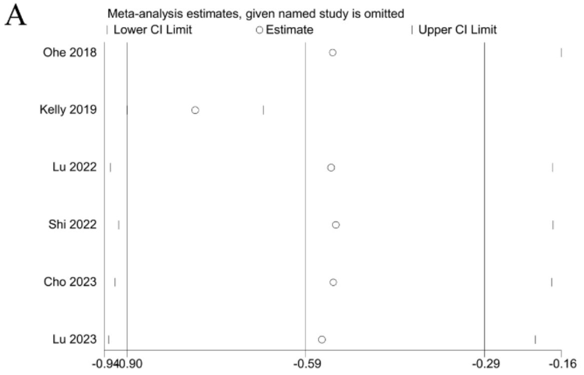

Study omitted	Estimate	[95% Conf.	Interval]
Ohe 2018	-.54661739	-.93580806	-.15742676
Kelly 2019	-.78063643	-.89634919	-.66492361
Lu 2022	-.54909199	-.92573643	-.17244759
Shi 2022	-.54118156	-.91097438	-.17138875
Cho 2023	-.54554033	-.91730195	-.17377874
Lu 2023	-.56507885	-.92860073	-.20155703
Combined	-.59286803	-.89726661	-.28846945

B

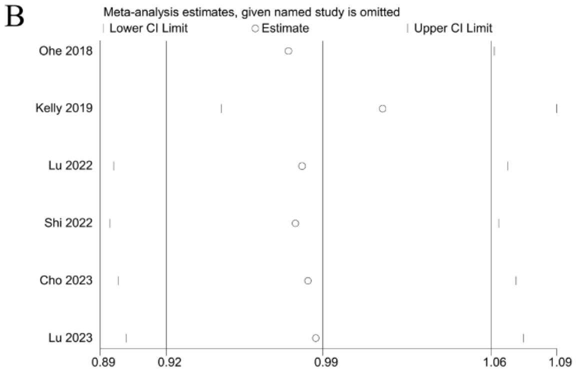

Study omitted	Estimate	[95% Conf.	Interval]
Ohe 2018	.97112149	.88845021	1.0614854
Kelly 2019	1.0126307	.94161844	1.0889983
Lu 2022	.97707921	.89433408	1.06748
Shi 2022	.97435039	.89271259	1.0634539
Cho 2023	.97986829	.89640361	1.0711045
Lu 2023	.98331112	.8999331	1.074414
Combined	.98623754	.91746507	1.0601651

C

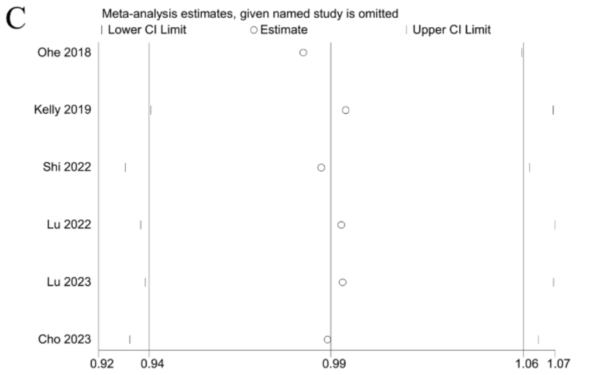

Study omitted	Estimate	[95% Conf.	Interval]
Ohe 2018	.98619151	.92207015	1.0547719
Kelly 2019	.99949408	.93841517	1.0645485
Shi 2022	.99181497	.93045127	1.0572256
Lu 2022	.99807894	.93520659	1.0651782
Lu 2023	.99859858	.93662304	1.064675
Cho 2023	.99382716	.93185568	1.05992
Combined	.99479582	.93785026	1.0551991

D

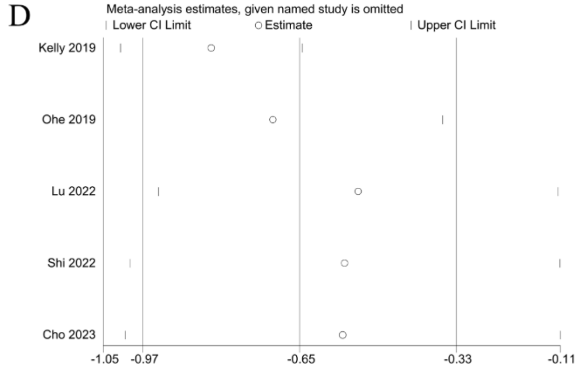

Study omitted	Estimate	[95% Conf.	Interval]
Kelly 2019	-.82936406	-1.0158218	-.64290625
Ohe 2019	-.70271379	-1.0514443	-.35398319
Lu 2022	-.52741355	-.93801945	-.1168076
Shi 2022	-.5550729	-.99639559	-.11375023
Cho 2023	-.55948067	-1.0063097	-.11265167
Combined	-.64805311	-.96968205	-.32642418

E

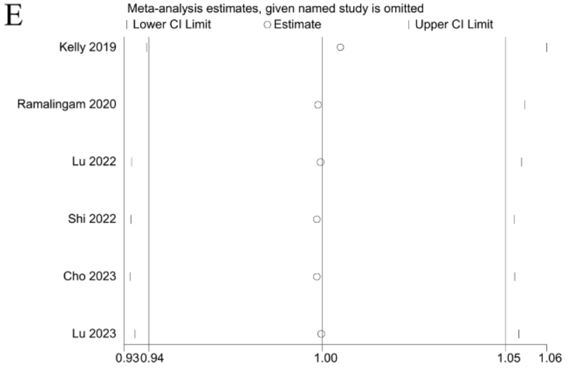

Study omitted	Estimate	[95% Conf.	Interval]
Kelly 2019	1.0009815	.94140601	1.0643271
Ramalingam 2020	.99408424	.93441838	1.05756
Lu 2022	.99490285	.93680227	1.0566069
Shi 2022	.99369442	.93653971	1.0543371
Cho 2023	.99366617	.93631798	1.0545268
Lu 2023	.99503893	.93776226	1.0558139
Combined	.99534983	.9421206	1.0515865

F

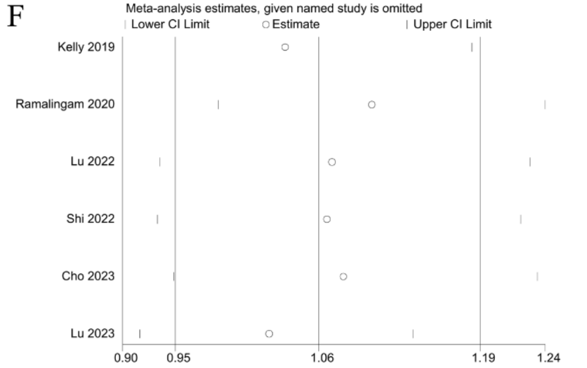

Study omitted	Estimate	[95% Conf.	Interval]
Kelly 2019	1.0355388	.90457529	1.1854631
Ramalingam 2020	1.1053249	.98167622	1.244548
Lu 2022	1.0731663	.93457866	1.2323048
Shi 2022	1.0689543	.93288434	1.2248714
Cho 2023	1.0823623	.94605863	1.238304
Lu 2023	1.0225407	.91870028	1.138118
Combined	1.0624917	.94695705	1.1921223

Table S1 PRISMA 2009 checklist.

Section/topic	#	Checklist item	Reported on page #
**TITLE**			1
Title	1	Identify the report as a systematic review, meta-analysis, or both.	1
**ABSTRACT**			2-3
Structured summary	2	Provide a structured summary including, as applicable: background; objectives; data sources; study eligibility criteria, participants, and interventions; study appraisal and synthesis methods; results; limitations; conclusions and implications of key findings; systematic review registration number.	2-3
**INTRODUCTION**			4-5
Rationale	3	Describe the rationale for the review in the context of what is already known.	4
Objectives	4	Provide an explicit statement of questions being addressed with reference to participants, interventions, comparisons, outcomes, and study design (PICOS).	4-5
**METHODS**			5-8
Protocol and registration	5	Indicate if a review protocol exists, if and where it can be accessed (e.g., Web address), and, if available, provide registration information including registration number.	5
Eligibility criteria	6	Specify study characteristics (e.g., PICOS, length of follow-up) and report characteristics (e.g., years considered, language, publication status) used as criteria for eligibility, giving rationale.	5
Information sources	7	Describe all information sources (e.g., databases with dates of coverage, contact with study authors to identify additional studies) in the search and date last searched.	5
Search	8	Present full electronic search strategy for at least one database, including any limits used, such that it could be repeated.	5-6
Study selection	9	State the process for selecting studies (i.e., screening, eligibility, included in systematic review, and, if applicable, included in the meta-analysis).	5-6
Data collection process	10	Describe method of data extraction from reports (e.g., piloted forms, independently, in duplicate) and any processes for obtaining and confirming data from investigators.	6
Data items	11	List and define all variables for which data were sought (e.g., PICOS, funding sources) and any assumptions and simplifications made.	6
Risk of bias in individual studies	12	Describe methods used for assessing risk of bias of individual studies (including specification of whether this was done at the study or outcome level), and how this information is to be used in any data synthesis.	6-7

Summary measures	13	State the principal summary measures (e.g., risk ratio, difference in means).	6-7
Synthesis of results	14	Describe the methods of handling data and combining results of studies, if done, including measures of consistency (e.g., I^2) for each meta-analysis.	7-8
**Section/topic**	**#**	**Checklist item**	**Reported on page #**
Risk of bias across studies	15	Specify any assessment of risk of bias that may affect the cumulative evidence (e.g., publication bias, selective reporting within studies).	7-8
Additional analyses	16	Describe methods of additional analyses (e.g., sensitivity or subgroup analyses, meta-regression), if done, indicating which were pre-specified.	7-8
**RESULTS**			8-12
Study selection	17	Give numbers of studies screened, assessed for eligibility, and included in the review, with reasons for exclusions at each stage, ideally with a flow diagram.	8
Study characteristics	18	For each study, present characteristics for which data were extracted (e.g., study size, PICOS, follow-up period) and provide the citations.	8
Risk of bias within studies	19	Present data on risk of bias of each study and, if available, any outcome level assessment (see item 12).	8-12
Results of individual studies	20	For all outcomes considered (benefits or harms), present, for each study: (a) simple summary data for each intervention group (b) effect estimates and confidence intervals, ideally with a forest plot.	8-12
Synthesis of results	21	Present results of each meta-analysis done, including confidence intervals and measures of consistency.	8-12
Risk of bias across studies	22	Present results of any assessment of risk of bias across studies (see Item 15).	12
Additional analysis	23	Give results of additional analyses, if done (e.g., sensitivity or subgroup analyses, meta-regression [see Item 16]).	12
**DISCUSSION**			12-16
Summary of evidence	24	Summarize the main findings including the strength of evidence for each main outcome; consider their relevance to key groups (e.g., healthcare providers, users, and policy makers).	12-15
Limitations	25	Discuss limitations at study and outcome level (e.g., risk of bias), and at review-level (e.g., incomplete retrieval of identified research, reporting bias).	15
Conclusions	26	Provide a general interpretation of the results in the context of other evidence, and implications for future research.	16
**FUNDING**			17-18
Funding	27	Describe sources of funding for the systematic review and other support (e.g., supply of data); role of funders for the systematic review.	17-18

Table S2 Search strategies.

**PubMed**<br>The database was searched on January 20, 2024, n=55.<br>Search Strategy:<br>**(((Osimertinib[Title/Abstract] OR Nazartinib[Title/Abstract] OR Rociletinib[Title/Abstract] OR Mavelertinib[Title/Abstract] OR Lazertinib[Title/Abstract] OR Olmutinib[Title/Abstract] OR Naquotinib[Title/Abstract] OR Almonertinib[Title/Abstract] OR Furmonertinib[Title/Abstract] OR Abivertinib[Title/Abstract] OR Rezivertinib[Title/Abstract] OR Limertinib[Title/Abstract] OR Befotertinib[Title/Abstract] OR Olafertinib[Title/Abstract] OR Keynatinib[Title/Abstract] OR Oritinib[Title/Abstract] OR TAS-121[Title/Abstract]) AND (Lung cancer[Title/Abstract] OR NSCLC[Title/Abstract] OR Lung adenocarcinoma [Title/Abstract] OR Lung squamous cell carcinoma [Title/Abstract])) AND (Randomized[Title/Abstract] OR Randomly[Title/Abstract] OR Randomised[Title/Abstract])**
**Web of Science**<br>The database was searched on January 20, 2024, n=54.<br>Search Strategy:<br>(Osimertinib or Nazartinib or Rociletinib or Mavelertinib or Lazertinib or Olmutinib or Naquotinib or Almonertinib or Furmonertinib or Abivertinib or Rezivertinib or Limertinib or Befotertinib or Olafertinib or Keynatinib or Oritinib or TAS-121 (Abstract)) ANS (Lung cancer or NSCLC or Lung adenocarcinoma or Lung squamous cell carcinoma (Abstract) AND (Randomized or Randomly or Randomised (Abstract))
**EMBASE**<br>The database was searched on January 20, 2024, n=168.<br>Search Strategy:<br>(osimertinib:ti,ab,kw OR nazartinib:ti,ab,kw OR rociletinib:ti,ab,kw OR mavelertinib:ti,ab,kw OR lazertinib:ti,ab,kw OR olmutinib:ti,ab,kw OR naquotinib:ti,ab,kw OR almonertinib:ti,ab,kw OR furmonertinib:ti,ab,kw OR abivertinib:ti,ab,kw OR rezivertinib:ti,ab,kw OR limertinib:ti,ab,kw OR befotertinib:ti,ab,kw OR olafertinib:ti,ab,kw OR keynatinib:ti,ab,kw OR oritinib:ti,ab,kw OR 'tas 121':ti,ab,kw) AND ('lung cancer':ti,ab,kw OR nsclc:ti,ab,kw OR 'lung adenocarcinoma':ti,ab,kw OR 'lung squamous cell carcinoma':ti,ab,kw) AND (randomized:ti,ab,kw OR randomly:ti,ab,kw OR randomised:ti,ab,kw)
**Cochrane Library**<br>The database was searched on January 20, 2024, n=129.<br>Search Strategy:<br>Osimertinib or Nazartinib or Rociletinib or Mavelertinib or Lazertinib or Olmutinib or Naquotinib or Almonertinib or Furmonertinib or Abivertinib or Rezivertinib or Limertinib or Befotertinib or Olafertinib or Keynatinib or Oritinib or TAS-121 in Title Abstract Keyword AND Randomized or Randomly or Randomised in Title Abstract Keyword AND Lung cancer or NSCLC or Lung adenocarcinoma or Lung squamous cell carcinoma in Title Abstract Keyword - (Word variations have been searched)
**ScienceDirect**<br>The database was searched on January 20, 2024, n=126.<br>Search Strategy:

Title, abstract, keywords: ((“Osimertinib” or “Nazartinib” or “Rociletinib” or “Mavelertinib” or “Lazertinib” or “Olmutinib” or “Naquotinib” or “Almonertinib” or “Furmonertinib” or “Abivertinib” or “Rezivertinib” or “Limertinib” or “Befotertinib” or “Olafertinib” or “Keynatinib” or “Oritinib” or “TAS-121”) and (“Lung cancer” or “NSCLC” or “Lung adenocarcinoma” or “Lung squamous cell carcinoma”) and (“randomized” OR “randomly” OR “randomised”))
**Scopus**<br>The database was searched on January 20, 2024, n=24.<br>Search Strategy:<br>(TITLE-ABS-KEY (osimertinib OR nazartinib OR rociletinib OR mavelertinib OR lazertinib OR olmutinib OR naquotinib OR almonertinib OR furmonertinib OR abivertinib OR rezivertinib OR limertinib OR befotertinib OR olafertinib OR keynatinib OR oritinib OR tas-121) AND TITLE-ABS-KEY (lung AND cancer OR nsclc OR lung AND adenocarcinoma OR lung AND squamous AND cell AND carcinoma) AND TITLE-ABS-KEY (randomized OR randomly OR randomised))

Note: The combined text and medical subject heading (MeSH) terms used were: “lung cancer”, “randomized” and Third generation EGFR TKIs (including Osimertinib, Nazartinib, Rociletinib, Mavelertinib, Lazertinib, Olmutinib, Naquotinib, Almonertinib, Furmonertinib, Abivertinib, Rezivertinib, Limertinib, Befotertinib, Olafertinib, Keynatinib, Oritinib, and TAS-121).

Table S3 Quality assessment of the included studies according to the Jadad scale.

	Study	Randomization	Masking	Accountability of all patients	Quality (score)
NCT02296125(FLAURA)	Cheng 2021 [17],Ramalingam 2020 [18], Ohe 2019 [19], Cho 2019 [20], Ohe 2018 [7], Reungwetwattana 2018 [21]	**	**	*	5
NCT04248829(LASER301)	Lee 2024 [22], Soo 2023 [23], Reungwetwattana 2023 [24], Cho 2023 [8]	**	**	*	5
NCT03787992(FURLONG)	Shi 2022 [9], Shi 2022 CNS [25]	**	**	*	5
NCT03849768(AENEAS)	Lu 2022 [10]	**	**	*	5
NCT02588261(SOLAR)	Kelly 2019 [11]	**	**	*	5
NCT04206072	Lu 2023 [12]	**	**	*	5

Table S4 GRADE quality assessment for the outcomes of survival, responses, and adverse events.

Primary outcomes	No. of Studies	No. of Participants		Differences (95%CI) [a]	Quality Assessment					Quality
		TGET	FGET		Risk of Bias[b]	Inconsistency	Indirectness	Imprecision	Publication Bias[c]	
**Patients' condition at data cutoff**										
Ongoing	6	397/1316	145/1311	2.74 [2.32, 3.24]	Low	No inconsistency	No indirectness	No imprecision	Unlikely	High
Discontinuation due to progressive disease	6	526/1316	815/1311	0.63 [0.54, 0.73]	Low	Serious (-1)	No indirectness	No imprecision	Unlikely	Medium
Discontinuation due to adverse events	6	131/1316	108/1311	1.44 [0.85, 2.43]	Low	Serious (-1)	No indirectness	No imprecision	Unlikely	Medium
**Survival**										
**PFS**	6	1316	1311	0.55 [0.41, 0.75]	Low	Serious (-1)	No indirectness	No imprecision	Unlikely	Medium
**Subgroup analysis of PFS**										
Age < 65 years	5	1263		0.41 [0.36, 0.48]	Low	No inconsistency	No indirectness	No imprecision	Unlikely	High
Age > 65 years	5	834		0.55 [0.46, 0.67]	Low	No inconsistency	No indirectness	No imprecision	Unlikely	High
Sex - Female	5	1315		0.43 [0.37, 0.50]	Low	No inconsistency	No indirectness	No imprecision	Unlikely	High
Sex - Male	5	782		0.50 [0.42, 0.60]	Low	No inconsistency	No indirectness	No imprecision	Unlikely	High
Smoking status - Active/Former smoker	5	638		0.51 [0.42, 0.62]	Low	No inconsistency	No indirectness	No imprecision	Unlikely	High
Smoking status - Non-smoker	5	1459		0.43 [0.37, 0.49]	Low	No inconsistency	No indirectness	No imprecision	Unlikely	High
ECOG PS - 0	5	576		0.37 [0.29, 0.46]	Low	No inconsistency	No indirectness	No imprecision	Unlikely	High
ECOG PS - 1	5	1513		0.50 [0.44, 0.57]	Low	No inconsistency	No indirectness	No imprecision	Unlikely	High
CNS metastases at baseline - Yes	5	567		0.45 [0.36, 0.55]	Low	No inconsistency	No indirectness	No imprecision	Unlikely	High
CNS metastases at baseline - No	5	1530		0.46 [0.40, 0.53]	Low	No inconsistency	No indirectness	No imprecision	Unlikely	High
EGFR mutation - Ex19del	5	1291		0.42 [0.36, 0.49]	Low	No inconsistency	No indirectness	No imprecision	Unlikely	High
EGFR mutation - L858R	5	806		0.52 [0.44, 0.61]	Low	No inconsistency	No indirectness	No imprecision	Unlikely	High
Race category - Asian	5	1755		0.48 [0.42, 0.54]	Low	No inconsistency	No indirectness	No imprecision	Unlikely	High
Race category - Non-asian	2	342		0.35 [0.26, 0.48]	Low	No inconsistency	No indirectness	No imprecision	Unlikely	High
TGET - Befotertinib	1	362		0.49 [0.36, 0.67]	Low	No inconsistency	No indirectness	No imprecision	Unlikely	High
TGET - Lazertinib	1	393		0.45 [0.34, 0.59]	Low	No inconsistency	No indirectness	No imprecision	Unlikely	High
TGET - Furmonertinib	1	357		0.44 [0.34, 0.57]	Low	No inconsistency	No indirectness	No imprecision	Unlikely	High
TGET - Aumolertinib	1	429		0.46 [0.36, 0.59]	Low	No inconsistency	No indirectness	No imprecision	Unlikely	High
TGET - Naquotinib	1	530		1.61 [1.09, 2.39]	Low	No inconsistency	No indirectness	No imprecision	Unlikely	High
TGET - Osimertinib	1	556		0.46 [0.37, 0.57]	Low	No inconsistency	No indirectness	No imprecision	Unlikely	High
**PFSR**										
6-month	6	1063/1316	1011/1311	1.05 [0.94, 1.17]	Low	Serious (-1)	No indirectness	No imprecision	Unlikely	Medium

12-month	6	813/1316	587/1311	1.36 [1.12, 1.65]	Low	Serious (-1)	No indirectness	No imprecision	Unlikely	Medium
18-month	5	553/1049	245/1048	2.25 [1.99, 2.55]	Low	No inconsistency	No indirectness	No imprecision	Unlikely	High
24-month	5	363/1049	138/1048	2.53 [1.82, 3.52]	Low	Serious (-1)	No indirectness	No imprecision	Unlikely	Medium
**PFSR for patients with CNS metastases**										
6-month	5	223/270	205/268	1.07 [0.98, 1.16]	Low	No inconsistency	No indirectness	No imprecision	Unlikely	High
12-month	5	177/270	123/268	1.48 [1.06, 2.08]	Low	Serious (-1)	No indirectness	No imprecision	Unlikely	Medium
18-month	5	138/270	60/268	2.38 [1.46, 3.90]	Low	Serious (-1)	No indirectness	No imprecision	Unlikely	Medium
24-month	4	77/207	30/210	5.00 [0.99, 25.27]	Low	Serious (-1)	No indirectness	No imprecision	Unlikely	Medium
**PFSR for patients without CNS metastases**										
6-month	4	494/555	424/551	1.16 [1.10, 1.22]	Low	No inconsistency	No indirectness	No imprecision	Unlikely	High
12-month	4	387/555	251/551	1.53 [1.38, 1.70]	Low	No inconsistency	No indirectness	No imprecision	Unlikely	High
18-month	4	293/555	135/551	2.15 [1.82, 2.54]	Low	No inconsistency	No indirectness	No imprecision	Unlikely	High
24-month	4	194/555	84/551	2.09 [1.28, 3.43]	Low	Serious (-1)	No indirectness	No imprecision	Unlikely	Medium
**PFSR for patients with Ex19del mutations**										
6-month	3	360/392	303/392	1.19 [1.12, 1.26]	Low	No inconsistency	No indirectness	No imprecision	Unlikely	High
12-month	3	296/392	188/392	1.57 [1.40, 1.77]	Low	No inconsistency	No indirectness	No imprecision	Unlikely	High
18-month	3	223/392	101/392	2.21 [1.83, 2.67]	Low	No inconsistency	No indirectness	No imprecision	Unlikely	High
24-month	3	140/392	58/392	2.41 [1.84, 3.17]	Low	No inconsistency	No indirectness	No imprecision	Unlikely	High
**PFSR for patients with L858R mutations**										
6-month	3	178/230	156/229	1.14 [1.01, 1.27]	Low	No inconsistency	No indirectness	No imprecision	Unlikely	High
12-month	3	130/230	82/229	1.58 [1.28, 1.94]	Low	No inconsistency	No indirectness	No imprecision	Unlikely	High
18-month	3	92/230	45/229	2.04 [1.50, 2.76]	Low	No inconsistency	No indirectness	No imprecision	Unlikely	High
24-month	3	77/230	10/229	6.27 [2.13, 18.45]	Low	Serious (-1)	No indirectness	No imprecision	Unlikely	Medium
**OS**	4	835	833	0.86 [0.74, 1.01]						
**OSR**										
6-month	4	793/835	777/833	1.02 [0.99, 1.04]	Low	No inconsistency	No indirectness	No imprecision	Unlikely	High
12-month	5	911/1049	888/1048	1.02 [0.99, 1.06]	Low	No inconsistency	No indirectness	No imprecision	Unlikely	High
18-month	4	656/835	618/833	1.05 [0.95, 1.15]	Low	Serious (-1)	No indirectness	No imprecision	Unlikely	Medium
24-month	4	579/835	512/833	1.12 [0.99, 1.26]	Low	Serious (-1)	No indirectness	No imprecision	Unlikely	Medium
30-month	2	299/461	252/457	1.18 [1.06, 1.31]	Low	No inconsistency	No indirectness	No imprecision	Unlikely	High
36-month	1	152/279	122/277	1.24 [1.04, 1.47]	Low	No inconsistency	No indirectness	No imprecision	Unlikely	High
**CNS-PFS**	4	353	350	0.48 [0.35, 0.66]	Low	No inconsistency	No indirectness	No imprecision	Unlikely	High
**CNS-PFSR**										
6-month	3	153/171	127/170	1.19 [1.08, 1.32]	Low	No inconsistency	No indirectness	No imprecision	Unlikely	High

12-month	3	128/171	85/170	1.51 [1.26, 1.79]	Low	No inconsistency	No indirectness	No imprecision	Unlikely	High
18-month	3	103/171	61/170	1.68 [1.33, 2.13]	Low	No inconsistency	No indirectness	No imprecision	Unlikely	High
24-month	1	36/61	18/67	2.20 [1.40, 3.43]	Low	No inconsistency	No indirectness	No imprecision	Unlikely	High
**Pathological responses**										
**Responses**										
ORR	6	914/1316	933/1311	0.98 [0.90, 1.07]	Low	Serious (-1)	No indirectness	No imprecision	Unlikely	Medium
DCR	6	1159/1316	1167/1311	0.99 [0.96, 1.03]	Low	Serious (-1)	No indirectness	No imprecision	Unlikely	Medium
CR	6	10/1316	6/1311	1.66 [0.61, 4.54]	Low	No inconsistency	No indirectness	No imprecision	Unlikely	High
PR	6	904/1316	927/1311	0.98 [0.90, 1.06]	Low	Serious (-1)	No indirectness	No imprecision	Unlikely	Medium
SD	6	245/1316	234/1311	1.01 [0.79, 1.29]	Low	Serious (-1)	No indirectness	No imprecision	Unlikely	Medium
PD	6	44/1316	48/1311	0.86 [0.42, 1.77]	Low	Serious (-1)	No indirectness	No imprecision	Unlikely	Medium
**CNS responses**										
CNS-ORR	4	70/76	58/89	1.40 [1.19, 1.65]	Low	No inconsistency	No indirectness	No imprecision	Unlikely	High
CNS-DCR	4	73/76	79/89	1.08 [0.99, 1.17]	Low	No inconsistency	No indirectness	No imprecision	Unlikely	High
CNS-CR	4	13/76	4/89	2.76 [1.07, 7.13]	Low	No inconsistency	No indirectness	No imprecision	Unlikely	High
CNS-PR	4	57/76	54/89	1.25 [1.02, 1.55]	Low	No inconsistency	No indirectness	No imprecision	Unlikely	High
CNS-SD	4	3/76	21/89	0.22 [0.08, 0.61]	Low	No inconsistency	No indirectness	No imprecision	Unlikely	High
CNS-PD	4	1/76	7/89	0.27 [0.06, 1.33]	Low	No inconsistency	No indirectness	No imprecision	Unlikely	High
**Duration of response**										
DOR	5	920	909	0.52 [0.38, 0.72]	Low	Serious (-1)	No indirectness	No imprecision	Unlikely	Medium
CNS-DOR	2	106	108	0.73 [0.26, 2.08]	Low	No inconsistency	No indirectness	No imprecision	Unlikely	High
**Estimated percentage remaining in response**										
EPR-6m	3	318/356	244/343	1.26 [1.03, 1.55]	Low	Serious (-1)	No indirectness	No imprecision	Unlikely	Medium
EPR-12m	5	548/817	280/814	1.93 [1.56, 2.39]	Low	Serious (-1)	No indirectness	No imprecision	Unlikely	Medium
EPR-18m	5	431/817	140/814	3.08 [2.61, 3.63]	Low	No inconsistency	No indirectness	No imprecision	Unlikely	High
EPR-24m	3	115/356	43/343	2.50 [1.23, 5.07]	Low	Serious (-1)	No indirectness	No imprecision	Unlikely	Medium
**Estimated percentage remaining in CNS response**										
CNS-EPR-6m	2	30/37	16/24	1.22 [0.88, 1.67]	Low	No inconsistency	No indirectness	No imprecision	Unlikely	High
CNS-EPR-12m	2	24/37	13/24	1.20 [0.77, 1.85]	Low	No inconsistency	No indirectness	No imprecision	Unlikely	High
CNS-EPR-18m	2	21/37	13/24	1.05 [0.66, 1.66]	Low	No inconsistency	No indirectness	No imprecision	Unlikely	High
CNS-EPR-24m	1	13/17	6/11	1.40 [0.77, 2.56]	Low	No inconsistency	No indirectness	No imprecision	Unlikely	High
**Depth of response**										
Depth of response	4	800	800	-0.05 [-0.06, -0.04]	Low	No inconsistency	No indirectness	No imprecision	Unlikely	High
Depth of CNS response	3	58	70	-0.12 [-0.28, 0.04]	Low	Serious (-1)	No indirectness	No imprecision	Unlikely	Medium

**Safety**										
**Safety summary**										
Total adverse events	6	1282/1316	1289/1311	0.99 [0.98, 1.01]	Low	Serious (-1)	No indirectness	No imprecision	Unlikely	Medium
Grade 3-5 adverse events	6	568/1316	519/1311	1.10 [0.93, 1.29]	Low	Serious (-1)	No indirectness	No imprecision	Unlikely	Medium
Serious adverse events	5	310/1134	264/1131	1.17 [1.02, 1.35]	Low	No inconsistency	No indirectness	No imprecision	Unlikely	High
Fatal adverse events	5	49/1134	40/1131	1.22 [0.81, 1.83]	Low	No inconsistency	No indirectness	No imprecision	Unlikely	High
Treatment-related adverse events	7	1073/1173	1057/1154	1.01 [0.96, 1.06]	Low	Serious (-1)	No indirectness	No imprecision	Unlikely	Medium
Grade 3-5 treatment-related adverse events	6	272/959	192/939	1.24 [0.73, 2.12]	Low	Serious (-1)	No indirectness	No imprecision	Unlikely	Medium
Serious treatment-related adverse events	6	96/991	75/974	1.15 [0.62, 2.12]	Low	Serious (-1)	No indirectness	No imprecision	Unlikely	Medium
Fatal treatment-related adverse events	5	5/777	2/759	2.10 [0.47, 9.32]	Low	No inconsistency	No indirectness	No imprecision	Unlikely	High
Discontinuation due to adverse events	6	134/1316	116/1311	1.20 [0.79, 1.84]	Low	Serious (-1)	No indirectness	No imprecision	Unlikely	Medium
Dose reduction due to adverse events	6	174/1316	100/1311	1.58 [0.81, 3.09]	Low	Serious (-1)	No indirectness	No imprecision	Unlikely	Medium
Dose interruption due to adverse events	6	368/1316	348/1311	1.04 [0.84, 1.29]	Low	Serious (-1)	No indirectness	No imprecision	Unlikely	Medium
**Any grade adverse Events**										
Diarrhea	6	478/1316	537/1311	0.79 [0.60, 1.04]	Low	Serious (-1)	No indirectness	No imprecision	Unlikely	Medium
Rash	6	398/1316	699/1311	0.54 [0.34, 0.86]	Low	Serious (-1)	No indirectness	No imprecision	Unlikely	Medium
Platelet count decreased	4	188/645	35/639	5.08 [1.72, 14.96]	Low	Serious (-1)	No indirectness	No imprecision	Unlikely	Medium
Elevated serum creatinine	1	43/182	8/180	5.32 [2.57, 10.99]	Low	No inconsistency	No indirectness	No imprecision	Unlikely	High
White blood cell count decreased	4	142/645	81/639	1.77 [1.19, 2.64]	Low	Serious (-1)	No indirectness	No imprecision	Unlikely	Medium
Peripheral sensory neuropathy	3	141/645	22/640	5.61 [1.38, 22.81]	Low	Serious (-1)	No indirectness	No imprecision	Unlikely	Medium
Dry skin	3	161/742	190/737	0.80 [0.42, 1.54]	Low	Serious (-1)	No indirectness	No imprecision	Unlikely	Medium
Deep vein thrombosis	1	39/182	1/180	38.57 [5.36, 277.75]	Low	No inconsistency	No indirectness	No imprecision	Unlikely	High
ALT increased	6	272/1316	479/1311	0.56 [0.41, 0.78]	Low	Serious (-1)	No indirectness	No imprecision	Unlikely	Medium
Hyponatremia	2	69/338	4/328	15.08 [5.88, 38.64]	Low	No inconsistency	No indirectness	No imprecision	Unlikely	High
Lipid metabolism diseases	1	36/182	17/180	2.09 [1.22, 3.59]	Low	No inconsistency	No indirectness	No imprecision	Unlikely	High
Paronychia	3	146/742	195/737	0.45 [0.14, 1.47]	Low	Serious (-1)	No indirectness	No imprecision	Unlikely	Medium
Urinary tract infection	4	124/645	106/639	1.16 [0.92, 1.47]	Low	No inconsistency	No indirectness	No imprecision	Unlikely	High
Cough	3	119/639	100/636	1.18 [0.93, 1.51]	Low	No inconsistency	No indirectness	No imprecision	Unlikely	High
AST increased	6	244/1316	437/1311	0.56 [0.45, 0.70]	Low	Serious (-1)	No indirectness	No imprecision	Unlikely	Medium
Renal symptoms	1	50/279	32/277	1.55 [1.03, 2.34]	Low	No inconsistency	No indirectness	No imprecision	Unlikely	High
Hyperuricaemia	1	32/182	14/180	2.26 [1.25, 4.09]	Low	No inconsistency	No indirectness	No imprecision	Unlikely	High
Anemia	5	184/1049	95/1048	1.90 [1.26, 2.87]	Low	Serious (-1)	No indirectness	No imprecision	Unlikely	Medium
Upper respiratory tract infection	4	149/853	106/851	1.40 [1.12, 1.77]	Low	No inconsistency	No indirectness	No imprecision	Unlikely	High
Weight increased	2	62/360	66/359	0.94 [0.68, 1.28]	Low	No inconsistency	No indirectness	No imprecision	Unlikely	High
Increased blood creatine phosphokinase	3	94/574	33/574	2.12 [0.79, 5.69]	Low	Serious (-1)	No indirectness	No imprecision	Unlikely	Medium

Headache	4	137/853	51/851	2.58 [1.14, 5.80]	Low	Serious (-1)	No indirectness	No imprecision	Unlikely	Medium
Fatigue	3	113/728	65/720	1.72 [1.29, 2.29]	Low	No inconsistency	No indirectness	No imprecision	Unlikely	High
Stomatitis	4	139/920	143/916	0.85 [0.39, 1.88]	Low	Serious (-1)	No indirectness	No imprecision	Unlikely	Medium
Decreased appetite	5	170/1138	147/1132	1.16 [0.80, 1.68]	Low	Serious (-1)	No indirectness	No imprecision	Unlikely	Medium
Proteinuria	2	37/253	22/245	1.63 [0.99, 2.68]	Low	No inconsistency	No indirectness	No imprecision	Unlikely	High
Constipation	4	120/835	84/833	1.42 [1.10, 1.85]	Low	No inconsistency	No indirectness	No imprecision	Unlikely	High
Elevated fibrin D-dimer	1	26/182	8/180	3.21 [1.50, 6.91]	Low	No inconsistency	No indirectness	No imprecision	Unlikely	High
Neutrophil count decreased	3	65/463	35/459	1.84 [1.24, 2.72]	Low	No inconsistency	No indirectness	No imprecision	Unlikely	High
Nausea	6	183/1316	134/1311	1.41 [0.91, 2.18]	Low	Serious (-1)	No indirectness	No imprecision	Unlikely	Medium
Pruritus	5	140/1049	125/1048	0.98 [0.62, 1.55]	Low	Serious (-1)	No indirectness	No imprecision	Unlikely	Medium
Musculoskeletal pain	3	85/639	22/636	2.62 [0.49, 14.06]	Low	Serious (-1)	No indirectness	No imprecision	Unlikely	Medium
Muscle spasms	1	26/196	7/197	3.73 [1.66, 8.40]	Low	No inconsistency	No indirectness	No imprecision	Unlikely	High
Blood lactate dehydrogenase increase	1	26/214	14/215	1.87 [1.00, 3.47]	Low	No inconsistency	No indirectness	No imprecision	Unlikely	High
Vomiting	4	97/853	62/851	1.56 [1.15, 2.11]	Low	No inconsistency	No indirectness	No imprecision	Unlikely	High
Insomnia	1	31/279	21/277	1.47 [0.86, 2.49]	Low	No inconsistency	No indirectness	No imprecision	Unlikely	High
Nasopharyngitis	1	31/279	16/277	1.92 [1.08, 3.44]	Low	No inconsistency	No indirectness	No imprecision	Unlikely	High
Dermatitis acneiform	1	21/196	27/197	0.78 [0.46, 1.33]	Low	No inconsistency	No indirectness	No imprecision	Unlikely	High
Back pain	2	48/457	48/456	1.00 [0.68, 1.46]	Low	No inconsistency	No indirectness	No imprecision	Unlikely	High
Pyrexia	2	48/461	31/457	1.48 [0.48, 4.62]	Low	Serious (-1)	No indirectness	No imprecision	Unlikely	Medium
Hematuria	2	26/253	37/245	0.81 [0.32, 2.03]	Low	No inconsistency	No indirectness	No imprecision	Unlikely	High
Dyspnea	3	65/639	41/636	1.58 [1.08, 2.29]	Low	No inconsistency	No indirectness	No imprecision	Unlikely	High
Oral ulcer	4	63/645	61/639	1.02 [0.73, 1.43]	Low	No inconsistency	No indirectness	No imprecision	Unlikely	High
ECG QTc prolongation	4	83/853	50/851	1.66 [1.18, 2.32]	Low	No inconsistency	No indirectness	No imprecision	Unlikely	High
Arthralgia	1	17/178	21/179	0.81 [0.44, 1.49]	Low	No inconsistency	No indirectness	No imprecision	Unlikely	High
Hypoalbuminemia	2	27/285	29/280	1.03 [0.38, 2.81]	Low	Serious (-1)	No indirectness	No imprecision	Unlikely	Medium
Dizziness	1	17/182	8/180	2.10 [0.93, 4.75]	Low	No inconsistency	No indirectness	No imprecision	Unlikely	High
Arrhythmia	2	32/360	35/359	0.91 [0.59, 1.40]	Low	No inconsistency	No indirectness	No imprecision	Unlikely	High
Weight decreased	4	57/645	61/639	1.07 [0.47, 2.44]	Low	Serious (-1)	No indirectness	No imprecision	Unlikely	Medium
Lymphocyte count decreased	2	22/249	8/244	2.65 [1.20, 5.85]	Low	No inconsistency	No indirectness	No imprecision	Unlikely	High
Hypokalemia	3	40/463	62/459	0.64 [0.44, 0.93]	Low	No inconsistency	No indirectness	No imprecision	Unlikely	High
Abdominal pain	2	31/360	17/359	1.81 [1.03, 3.18]	Low	No inconsistency	No indirectness	No imprecision	Unlikely	High
Chest pain	3	36/431	22/424	1.61 [0.96, 2.67]	Low	No inconsistency	No indirectness	No imprecision	Unlikely	High
Gamma-glutamyl transferase increase	3	38/463	59/459	0.64 [0.43, 0.94]	Low	No inconsistency	No indirectness	No imprecision	Unlikely	High
Alopecia	1	22/279	35/277	0.62 [0.38, 1.04]	Low	No inconsistency	No indirectness	No imprecision	Unlikely	High
Edema	1	12/182	1/180	11.87 [1.56, 90.33]	Low	No inconsistency	No indirectness	No imprecision	Unlikely	High
Pneumonia	3	37/574	29/574	1.27 [0.80, 2.04]	Low	No inconsistency	No indirectness	No imprecision	Unlikely	High
Hypertension	3	37/574	39/574	0.95 [0.61, 1.46]	Low	No inconsistency	No indirectness	No imprecision	Unlikely	High

Abnormal liver function	3	34/574	51/574	0.56 [0.20, 1.54]	Low	Serious (-1)	No indirectness	No imprecision	Unlikely	Medium
Chest discomfort	3	25/431	23/424	1.05 [0.61, 1.81]	Low	No inconsistency	No indirectness	No imprecision	Unlikely	High
Hypocalcemia	2	14/249	14/244	0.96 [0.47, 1.96]	Low	No inconsistency	No indirectness	No imprecision	Unlikely	High
Pleural effusion	2	20/360	18/359	1.11 [0.60, 2.05]	Low	No inconsistency	No indirectness	No imprecision	Unlikely	High
Blood bilirubin increased	3	30/574	79/574	0.37 [0.16, 0.88]	Low	Serious (-1)	No indirectness	No imprecision	Unlikely	Medium
Gastrointestinal diseases	2	18/360	7/359	2.44 [1.07, 5.60]	Low	No inconsistency	No indirectness	No imprecision	Unlikely	High
Hyperglycemia	1	8/182	13/180	0.61 [0.26, 1.43]	Low	No inconsistency	No indirectness	No imprecision	Unlikely	High
Pulmonary embolism	2	15/360	2/359	7.46 [1.72, 32.45]	Low	No inconsistency	No indirectness	No imprecision	Unlikely	High
Increased amylase	1	7/178	9/179	0.78 [0.30, 2.05]	Low	No inconsistency	No indirectness	No imprecision	Unlikely	High
Epistaxis	1	6/178	9/179	0.67 [0.24, 1.84]	Low	No inconsistency	No indirectness	No imprecision	Unlikely	High
Pericardial diseases	2	12/360	8/359	1.49 [0.62, 3.59]	Low	No inconsistency	No indirectness	No imprecision	Unlikely	High
Hemoptysis	1	5/182	10/180	0.49 [0.17, 1.42]	Low	No inconsistency	No indirectness	No imprecision	Unlikely	High
Contrast agent allergy	1	3/178	1/179	3.02 [0.32, 28.73]	Low	No inconsistency	No indirectness	No imprecision	Unlikely	High
Protrusion of intervertebral disc	1	3/178	1/179	3.02 [0.32, 28.73]	Low	No inconsistency	No indirectness	No imprecision	Unlikely	High
Hyperkalemia	1	3/178	3/179	1.01 [0.21, 4.92]	Low	No inconsistency	No indirectness	No imprecision	Unlikely	High
Cystitis	1	3/178	0/179	7.04 [0.37, 135.29]	Low	No inconsistency	No indirectness	No imprecision	Unlikely	High
Atrial fibrillation	2	6/360	9/359	0.68 [0.25, 1.81]	Low	No inconsistency	No indirectness	No imprecision	Unlikely	High
Gingivitis	1	2/178	1/179	2.01 [0.18, 21.98]	Low	No inconsistency	No indirectness	No imprecision	Unlikely	High
Increased bilirubin conjugated	1	2/178	7/179	0.29 [0.06, 1.36]	Low	No inconsistency	No indirectness	No imprecision	Unlikely	High
Cerebral infarction	1	2/178	0/179	5.03 [0.24, 103.99]	Low	No inconsistency	No indirectness	No imprecision	Unlikely	High
Asthma	1	1/178	3/179	0.34 [0.04, 3.19]	Low	No inconsistency	No indirectness	No imprecision	Unlikely	High
Cholecystitis	1	1/178	0/179	3.02 [0.12, 73.56]	Low	No inconsistency	No indirectness	No imprecision	Unlikely	High
Pancreatitis	1	1/178	0/179	3.02 [0.12, 73.56]	Low	No inconsistency	No indirectness	No imprecision	Unlikely	High
Decreased fibrinogen	1	1/178	0/179	3.02 [0.12, 73.56]	Low	No inconsistency	No indirectness	No imprecision	Unlikely	High
Furuncle	1	0/178	1/179	0.34 [0.01, 8.17]	Low	No inconsistency	No indirectness	No imprecision	Unlikely	High
**Grade 3-5 adverse Events**										
Hyponatremia	2	63/546	6/540	7.50 [0.54, 105.21]	Low	No inconsistency	No indirectness	No imprecision	Unlikely	High
Diarrhea	6	34/1249	17/1243	1.79 [0.89, 3.60]	Low	No inconsistency	No indirectness	No imprecision	Unlikely	High
Platelet count decreased	4	23/853	3/851	3.77 [0.85, 16.76]	Low	No inconsistency	No indirectness	No imprecision	Unlikely	High
Increased blood creatine phosphokinase	3	15/574	2/574	2.76 [0.07, 115.54]	Low	No inconsistency	No indirectness	No imprecision	Unlikely	High
Pulmonary embolism	3	16/639	2/636	5.89 [1.53, 22.71]	Low	No inconsistency	No indirectness	No imprecision	Unlikely	High
ALT increased	6	31/1249	99/1243	0.25 [0.09, 0.68]	Low	Serious (-1)	No indirectness	No imprecision	Unlikely	Medium
Lipid metabolism diseases	1	4/182	1/180	3.96 [0.45, 35.05]	Low	No inconsistency	No indirectness	No imprecision	Unlikely	High
Hypertension	4	18/853	12/851	1.59 [0.43, 5.87]	Low	Serious (-1)	No indirectness	No imprecision	Unlikely	Medium
Anemia	5	20/982	11/980	1.63 [0.72, 3.66]	Low	No inconsistency	No indirectness	No imprecision	Unlikely	High
Pneumonia	4	17/853	18/851	0.98 [0.45, 2.14]	Low	No inconsistency	No indirectness	No imprecision	Unlikely	High

Renal symptoms	1	5/279	1/277	4.96 [0.58, 42.22]	Low	No inconsistency	No indirectness	No imprecision	Unlikely	High
Ejection fraction decrease	1	5/279	1/277	4.96 [0.58, 42.22]	Low	No inconsistency	No indirectness	No imprecision	Unlikely	High
Fatigue	3	12/728	3/720	3.38 [0.56, 20.26]	Low	No inconsistency	No indirectness	No imprecision	Unlikely	High
Decreased appetite	5	16/1071	9/1064	1.66 [0.72, 3.83]	Low	No inconsistency	No indirectness	No imprecision	Unlikely	High
Neutrophil count decreased	3	10/671	1/671	5.22 [1.14, 23.82]	Low	No inconsistency	No indirectness	No imprecision	Unlikely	High
Hypokalemia	3	10/671	12/671	0.84 [0.36, 1.95]	Low	No inconsistency	No indirectness	No imprecision	Unlikely	High
Pathological fracture	1	4/279	2/277	0.20 [0.01, 4.12]	Low	No inconsistency	No indirectness	No imprecision	Unlikely	High
White blood cell count decreased	4	12/853	2/851	3.77 [1.04, 13.60]	Low	No inconsistency	No indirectness	No imprecision	Unlikely	High
ECG QTc prolongation	4	12/853	10/851	1.22 [0.51, 2.88]	Low	No inconsistency	No indirectness	No imprecision	Unlikely	High
Gamma-glutamyl transferase increase	3	8/671	6/671	1.42 [0.24, 8.56]	Low	No inconsistency	No indirectness	No imprecision	Unlikely	High
Lymphocyte count decreased	2	5/457	0/456	5.45 [0.63, 47.00]	Low	No inconsistency	No indirectness	No imprecision	Unlikely	High
Deep vein thrombosis	2	5/461	1/457	2.84 [0.31, 25.75]	Low	No inconsistency	No indirectness	No imprecision	Unlikely	High
Cataract disorder	1	3/279	1/277	2.98 [0.31, 28.46]	Low	No inconsistency	No indirectness	No imprecision	Unlikely	High
Sepsis	1	3/279	3/277	1.99 [0.37, 10.75]	Low	No inconsistency	No indirectness	No imprecision	Unlikely	High
Peripheral sensory neuropathy	3	6/578	1/572	2.86 [0.52, 15.60]	Low	No inconsistency	No indirectness	No imprecision	Unlikely	High
Dermatitis acneiform	2	4/408	1/406	2.83 [0.43, 18.49]	Low	No inconsistency	No indirectness	No imprecision	Unlikely	High
AST increased	6	12/1249	67/1243	0.20 [0.11, 0.36]	Low	No inconsistency	No indirectness	No imprecision	Unlikely	High
Dyspnea	3	6/639	4/636	1.32 [0.36, 4.81]	Low	No inconsistency	No indirectness	No imprecision	Unlikely	High
Pericardial diseases	2	3/360	3/359	0.94 [0.07, 12.95]	Low	No inconsistency	No indirectness	No imprecision	Unlikely	High
Pleural effusion	3	5/639	3/636	1.27 [0.27, 5.94]	Low	No inconsistency	No indirectness	No imprecision	Unlikely	High
Headache	4	6/853	0/851	3.92 [0.83, 18.54]	Low	No inconsistency	No indirectness	No imprecision	Unlikely	High
Urinary tract infection	4	5/853	2/851	1.75 [0.41, 7.43]	Low	No inconsistency	No indirectness	No imprecision	Unlikely	High
Abnormal liver function	4	5/853	16/851	0.32 [0.12, 0.88]	Low	No inconsistency	No indirectness	No imprecision	Unlikely	High
Gingivitis	1	1/178	0/179	3.02 [0.12, 73.56]	Low	No inconsistency	No indirectness	No imprecision	Unlikely	High
Contrast agent allergy	1	1/178	1/179	1.01 [0.06, 15.95]	Low	No inconsistency	No indirectness	No imprecision	Unlikely	High
Epistaxis	1	1/178	1/179	1.01 [0.06, 15.95]	Low	No inconsistency	No indirectness	No imprecision	Unlikely	High
Protrusion of intervertebral disc	1	1/178	0/179	3.02 [0.12, 73.56]	Low	No inconsistency	No indirectness	No imprecision	Unlikely	High
Hyperkalemia	1	1/178	2/179	0.50 [0.05, 5.50]	Low	No inconsistency	No indirectness	No imprecision	Unlikely	High
Cholecystitis	1	1/178	0/179	3.02 [0.12, 73.56]	Low	No inconsistency	No indirectness	No imprecision	Unlikely	High
Decreased fibrinogen	1	1/178	0/179	3.02 [0.12, 73.56]	Low	No inconsistency	No indirectness	No imprecision	Unlikely	High
Increased amylase	1	1/178	1/179	1.01 [0.06, 15.95]	Low	No inconsistency	No indirectness	No imprecision	Unlikely	High
Nausea	6	7/1249	2/1243	2.43 [0.60, 9.84]	Low	No inconsistency	No indirectness	No imprecision	Unlikely	High
Rash	6	7/1249	46/1243	0.25 [0.07, 0.90]	Low	No inconsistency	No indirectness	No imprecision	Unlikely	High
Abdominal pain	3	3/639	7/636	0.54 [0.07, 4.11]	Low	No inconsistency	No indirectness	No imprecision	Unlikely	High

Weight decreased	4	4/853	4/851	0.95 [0.15, 5.85]	Low	No inconsistency	No indirectness	No imprecision	Unlikely	High
Upper respiratory tract infection	4	4/853	4/851	0.99 [0.24, 4.10]	Low	No inconsistency	No indirectness	No imprecision	Unlikely	High
Pancreatitis	2	2/457	0/456	3.00 [0.31, 28.71]	Low	No inconsistency	No indirectness	No imprecision	Unlikely	High
Cerebral infarction	2	2/457	1/456	1.60 [0.20, 12.94]	Low	No inconsistency	No indirectness	No imprecision	Unlikely	High
Pneumothorax	1	1/279	2/277	0.50 [0.05, 5.44]	Low	No inconsistency	No indirectness	No imprecision	Unlikely	High
Acute psychosis	1	1/279	0/277	2.98 [0.12, 72.80]	Low	No inconsistency	No indirectness	No imprecision	Unlikely	High
Acute myocardial infarction	1	1/279	0/277	2.98 [0.12, 72.80]	Low	No inconsistency	No indirectness	No imprecision	Unlikely	High
Anxiety	1	1/279	0/277	2.98 [0.12, 72.80]	Low	No inconsistency	No indirectness	No imprecision	Unlikely	High
Cognitive disorder	1	1/279	0/277	0.99 [0.06, 15.79]	Low	No inconsistency	No indirectness	No imprecision	Unlikely	High
Interstitial lung disease	1	1/279	1/277	0.99 [0.20, 4.88]	Low	No inconsistency	No indirectness	No imprecision	Unlikely	High
Paronychia	4	3/853	7/848	0.64 [0.17, 2.37]	Low	No inconsistency	No indirectness	No imprecision	Unlikely	High
Atrial fibrillation	3	2/639	0/636	3.00 [0.31, 28.71]	Low	No inconsistency	No indirectness	No imprecision	Unlikely	High
Chest discomfort	2	1/360	2/359	0.62 [0.08, 5.03]	Low	No inconsistency	No indirectness	No imprecision	Unlikely	High
Stomatitis	4	2/853	5/848	0.57 [0.13, 2.40]	Low	No inconsistency	No indirectness	No imprecision	Unlikely	High
Cystitis	2	1/457	2/456	0.74 [0.05, 10.64]	Low	No inconsistency	No indirectness	No imprecision	Unlikely	High
Hemoptysis	2	1/461	4/457	0.25 [0.03, 2.21]	Low	No inconsistency	No indirectness	No imprecision	Unlikely	High
Edema	2	1/461	1/457	0.99 [0.06, 15.79]	Low	No inconsistency	No indirectness	No imprecision	Unlikely	High
Pruritus	5	2/982	0/980	2.99 [0.31, 28.60]	Low	No inconsistency	No indirectness	No imprecision	Unlikely	High
Blood bilirubin increased	3	1/574	1/574	1.01 [0.06, 15.95]	Low	No inconsistency	No indirectness	No imprecision	Unlikely	High
Musculoskeletal pain	3	1/639	2/636	0.74 [0.05, 10.31]	Low	No inconsistency	No indirectness	No imprecision	Unlikely	High
Gastrointestinal diseases	3	1/639	3/636	0.51 [0.09, 2.96]	Low	No inconsistency	No indirectness	No imprecision	Unlikely	High
Dry skin	3	1/675	4/669	0.33 [0.05, 2.09]	Low	No inconsistency	No indirectness	No imprecision	Unlikely	High
Vomiting	4	1/853	4/851	0.54 [0.02, 14.19]	Low	No inconsistency	No indirectness	No imprecision	Unlikely	High
Oral ulcer	4	1/853	2/851	0.73 [0.05, 10.38]	Low	No inconsistency	No indirectness	No imprecision	Unlikely	High
Pyrexia	2	0/461	1/457	0.33 [0.01, 8.09]	Low	No inconsistency	No indirectness	No imprecision	Unlikely	High
Hyperuricaemia	1	0/182	1/180	0.33 [0.01, 8.04]	Low	Serious (-1)	No indirectness	No imprecision	Unlikely	Medium
Constipation	4	0/768	9/765	0.05 [0.00, 0.89]	Low	No inconsistency	No indirectness	No imprecision	Unlikely	High
Chest pain	2	0/360	2/359	0.20 [0.01, 4.09]	Low	No inconsistency	No indirectness	No imprecision	Unlikely	High
Muscle spasms	1	0/129	0/129	-	Low	No inconsistency	No indirectness	No imprecision	Unlikely	High
Hematuria	1	0/182	0/180	-	Low	No inconsistency	No indirectness	No imprecision	Unlikely	High
Increased bilirubin conjugated	1	0/178	1/179	0.34 [0.01, 8.17]	Low	Serious (-1)	No indirectness	No imprecision	Unlikely	Medium
Cough	3	0/639	1/636	0.33 [0.01, 8.09]	Low	No inconsistency	No indirectness	No imprecision	Unlikely	High
Weight increased	2	0/360	1/359	0.33 [0.01, 8.04]	Low	No inconsistency	No indirectness	No imprecision	Unlikely	High
Dizziness	1	0/182	0/180	-	Low	No inconsistency	No indirectness	No imprecision	Unlikely	High

Insomnia	1	0/279	0/277	-	Low	No inconsistency	No indirectness	No imprecision	Unlikely	High
Alopecia	1	0/279	0/277	-	Low	No inconsistency	No indirectness	No imprecision	Unlikely	High
Nasopharyngitis	1	0/279	0/277	-	Low	No inconsistency	No indirectness	No imprecision	Unlikely	High
Hyperglycemia	2	0/461	2/457	0.20 [0.01, 4.12]	Low	No inconsistency	No indirectness	No imprecision	Unlikely	High
Hypocalcemia	1	0/178	1/179	0.34 [0.01, 8.17]	Low	No inconsistency	No indirectness	No imprecision	Unlikely	High
Asthma	1	0/178	2/179	0.20 [0.01, 4.16]	Low	No inconsistency	No indirectness	No imprecision	Unlikely	High
Furuncle	1	0/178	1/179	0.34 [0.01, 8.17]	Low	No inconsistency	No indirectness	No imprecision	Unlikely	High
Arrhythmia	2	0/360	1/359	0.34 [0.01, 8.17]	Low	No inconsistency	No indirectness	No imprecision	Unlikely	High
Proteinuria	1	0/182	0/180	-	Low	No inconsistency	No indirectness	No imprecision	Unlikely	High
Hypoalbuminemia	2	0/493	2/492	0.20 [0.01, 4.12]	Low	No inconsistency	No indirectness	No imprecision	Unlikely	High
Blood lactate dehydrogenase increase	1	0/214	0/215	-	Low	No inconsistency	No indirectness	No imprecision	Unlikely	High
Elevated serum creatinine	1	0/214	0/215	-	Low	No inconsistency	No indirectness	No imprecision	Unlikely	High
Back pain	2	0/457	0/456	-	Low	No inconsistency	No indirectness	No imprecision	Unlikely	High
Elevated fibrin D-dimer	1	0/182	0/180	-	Low	No inconsistency	No indirectness	No imprecision	Unlikely	High

Abbreviations: ALT: Alanine aminotransferase; AST: Aspartate aminotransferase; CI: confidence interval; CNS: Central nervous system; CNS-DOR: Duration of central nervous system response; CNS-PFS: Central nervous system-Progression-free survival; CNS-PFSR: CNS-PFS: Central nervous system-Progression-free survival rate; CR: Complete response; DCR: Disease control rate; DOR: Duration of response; ECG: Electrocardiogram; EPR: Estimated percentage remaining in response; FGET: First generation EGFR tyrosine kinase inhibitor; GRADE: Grading of Recommendations, Assessment, Development, and Evaluation; HR: Hazard ratio; MD: Mean difference; ORR: Objective response rate; OS: Overall survival; OSR: Overall survival rate; P: Probability; PD: Progressive disease; PFS: Progression-free survival; PFSR: Progression-free survival rate; PR: Partial response; RR: Risk ratio; SD: Stable disease; TGET: Third generation EGFR tyrosine kinase inhibitor.

[a] Differences: hazard ratio (HR) for OS, PFS, CNS-PFS and duration of response; risk ratios (RR) for OSR, PFSR, CNS-PFSR, summary of adverse events, total adverse events and grade 3-5 adverse events.

[b] Risk of bias assessed using the Jadad scale for randomized controlled trials.

[c] Publication bias was explored through visual inspection of the funnel plots.

Table S5 Total adverse events.

Adverse events	Studies involved	TGET		FGET		Risk ratio [95% CI]	P
		Event/total	%	Event/total	%		
Diarrhea	6	478/1316	36.32%	537/1311	40.96%	0.79 [0.60, 1.04]	0.09
Rash	6	398/1316	30.24%	699/1311	53.32%	0.54 [0.34, 0.86]	0.01
Platelet count decreased	4	188/645	29.15%	35/639	5.48%	5.08 [1.72, 14.96]	0.003
Elevated serum creatinine	1	43/182	23.63%	8/180	4.44%	5.32 [2.57, 10.99]	<0.00001
White blood cell count decreased	4	142/645	22.02%	81/639	12.68%	1.77 [1.19, 2.64]	0.005
Peripheral sensory neuropathy	3	141/645	21.86%	22/640	3.44%	5.61 [1.38, 22.81]	0.02
Dry skin	3	161/742	21.70%	190/737	25.78%	0.80 [0.42, 1.54]	0.51
Deep vein thrombosis	1	39/182	21.43%	1/180	0.56%	38.57 [5.36, 277.75]	0.0003
ALT increased	6	272/1316	20.67%	479/1311	36.54%	0.56 [0.41, 0.78]	0.0005
Hyponatremia	2	69/338	20.41%	4/328	1.22%	15.08 [5.88, 38.64]	<0.00001
Lipid metabolism diseases	1	36/182	19.78%	17/180	9.44%	2.09 [1.22, 3.59]	0.007
Paronychia	3	146/742	19.68%	195/737	26.46%	0.45 [0.14, 1.47]	0.18
Urinary tract infection	4	124/645	19.22%	106/639	16.59%	1.16 [0.92, 1.47]	0.21
Cough	3	119/639	18.62%	100/636	15.72%	1.18 [0.93, 1.51]	0.17
AST increased	6	244/1316	18.54%	437/1311	33.33%	0.56 [0.45, 0.70]	<0.00001
Renal symptoms	1	50/279	17.92%	32/277	11.55%	1.55 [1.03, 2.34]	0.04
Hyperuricaemia	1	32/182	17.58%	14/180	7.78%	2.26 [1.25, 4.09]	0.007
Anemia	5	184/1049	17.54%	95/1048	9.06%	1.90 [1.26, 2.87]	0.002
Upper respiratory tract infection	4	149/853	17.47%	106/851	12.46%	1.40 [1.12, 1.77]	0.004
Weight increased	2	62/360	17.22%	66/359	18.38%	0.94 [0.68, 1.28]	0.68
Increased blood creatine phosphokinase	3	94/574	16.38%	33/574	5.75%	2.12 [0.79, 5.69]	0.14
Headache	4	137/853	16.06%	51/851	5.99%	2.58 [1.14, 5.80]	0.02
Fatigue	3	113/728	15.52%	65/720	9.03%	1.72 [1.29, 2.29]	0.0002
Stomatitis	4	139/920	15.11%	143/916	15.61%	0.85 [0.39, 1.88]	0.69
Decreased appetite	5	170/1138	14.94%	147/1132	12.99%	1.16 [0.80, 1.68]	0.45
Proteinuria	2	37/253	14.62%	22/245	8.98%	1.63 [0.99, 2.68]	0.05
Constipation	4	120/835	14.37%	84/833	10.08%	1.42 [1.10, 1.85]	0.008
Elevated fibrin D-dimer	1	26/182	14.29%	8/180	4.44%	3.21 [1.50, 6.91]	0.003
Neutrophil count decreased	3	65/463	14.04%	35/459	7.63%	1.84 [1.24, 2.72]	0.002
Nausea	6	183/1316	13.91%	134/1311	10.22%	1.41 [0.91, 2.18]	0.12

Pruritus	5	140/1049	13.35%	125/1048	11.93%	0.98 [0.62, 1.55]	0.94
Musculoskeletal pain	3	85/639	13.30%	22/636	3.46%	2.62 [0.49, 14.06]	0.26
Muscle spasms	1	26/196	13.27%	7/197	3.55%	3.73 [1.66, 8.40]	0.001
Blood lactate dehydrogenase increase	1	26/214	12.15%	14/215	6.51%	1.87 [1.00, 3.47]	0.05
Vomiting	4	97/853	11.37%	62/851	7.29%	1.56 [1.15, 2.11]	0.004
Insomnia	1	31/279	11.11%	21/277	7.58%	1.47 [0.86, 2.49]	0.16
Nasopharyngitis	1	31/279	11.11%	16/277	5.78%	1.92 [1.08, 3.44]	0.03
Dermatitis acneiform	1	21/196	10.71%	27/197	13.71%	0.78 [0.46, 1.33]	0.37
Back pain	2	48/457	10.50%	48/456	10.53%	1.00 [0.68, 1.46]	0.99
Pyrexia	2	48/461	10.41%	31/457	6.78%	1.48 [0.48, 4.62]	0.5
Hematuria	2	26/253	10.28%	37/245	15.10%	0.81 [0.32, 2.03]	0.65
Dyspnea	3	65/639	10.17%	41/636	6.45%	1.58 [1.08, 2.29]	0.02
Oral ulcer	4	63/645	9.77%	61/639	9.55%	1.02 [0.73, 1.43]	0.91
ECG QTc prolongation	4	83/853	9.73%	50/851	5.88%	1.66 [1.18, 2.32]	0.003
Arthralgia	1	17/178	9.55%	21/179	11.73%	0.81 [0.44, 1.49]	0.51
Hypoalbuminemia	2	27/285	9.47%	29/280	10.36%	1.03 [0.38, 2.81]	0.95
Dizziness	1	17/182	9.34%	8/180	4.44%	2.10 [0.93, 4.75]	0.07
Arrhythmia	2	32/360	8.89%	35/359	9.75%	0.91 [0.59, 1.40]	0.66
Weight decreased	4	57/645	8.84%	61/639	9.55%	1.07 [0.47, 2.44]	0.88
Lymphocyte count decreased	2	22/249	8.84%	8/244	3.28%	2.65 [1.20, 5.85]	0.02
Hypokalemia	3	40/463	8.64%	62/459	13.51%	0.64 [0.44, 0.93]	0.02
Abdominal pain	2	31/360	8.61%	17/359	4.74%	1.81 [1.03, 3.18]	0.04
Chest pain	3	36/431	8.35%	22/424	5.19%	1.61 [0.96, 2.67]	0.07
Gamma-glutamyl transferase increase	3	38/463	8.21%	59/459	12.85%	0.64 [0.43, 0.94]	0.02
Alopecia	1	22/279	7.89%	35/277	12.64%	0.62 [0.38, 1.04]	0.07
Edema	1	12/182	6.59%	1/180	0.56%	11.87 [1.56, 90.33]	0.02
Pneumonia	3	37/574	6.45%	29/574	5.05%	1.27 [0.80, 2.04]	0.31
Hypertension	3	37/574	6.45%	39/574	6.79%	0.95 [0.61, 1.46]	0.81
Abnormal liver function	3	34/574	5.92%	51/574	8.89%	0.56 [0.20, 1.54]	0.26
Chest discomfort	3	25/431	5.80%	23/424	5.42%	1.05 [0.61, 1.81]	0.85
Hypocalcemia	2	14/249	5.62%	14/244	5.74%	0.96 [0.47, 1.96]	0.91
Pleural effusion	2	20/360	5.56%	18/359	5.01%	1.11 [0.60, 2.05]	0.75
Blood bilirubin increased	3	30/574	5.23%	79/574	13.76%	0.37 [0.16, 0.88]	0.02
Gastrointestinal diseases	2	18/360	5.00%	7/359	1.95%	2.44 [1.07, 5.60]	0.03

Hyperglycemia	1	8/182	4.40%	13/180	7.22%	0.61 [0.26, 1.43]	0.26
Pulmonary embolism	2	15/360	4.17%	2/359	0.56%	7.46 [1.72, 32.45]	0.007
Increased amylase	1	7/178	3.93%	9/179	5.03%	0.78 [0.30, 2.05]	0.62
Epistaxis	1	6/178	3.37%	9/179	5.03%	0.67 [0.24, 1.84]	0.44
Pericardial diseases	2	12/360	3.33%	8/359	2.23%	1.49 [0.62, 3.59]	0.38
Hemoptysis	1	5/182	2.75%	10/180	5.56%	0.49 [0.17, 1.42]	0.19
Contrast agent allergy	1	3/178	1.69%	1/179	0.56%	3.02 [0.32, 28.73]	0.34
Protrusion of intervertebral disc	1	3/178	1.69%	1/179	0.56%	3.02 [0.32, 28.73]	0.34
Hyperkalemia	1	3/178	1.69%	3/179	1.68%	1.01 [0.21, 4.92]	0.99
Cystitis	1	3/178	1.69%	0/179	0.00%	7.04 [0.37, 135.29]	0.2
Atrial fibrillation	2	6/360	1.67%	9/359	2.51%	0.68 [0.25, 1.81]	0.44
Gingivitis	1	2/178	1.12%	1/179	0.56%	2.01 [0.18, 21.98]	0.57
Increased bilirubin conjugated	1	2/178	1.12%	7/179	3.91%	0.29 [0.06, 1.36]	0.12
Cerebral infarction	1	2/178	1.12%	0/179	0.00%	5.03 [0.24, 103.99]	0.3
Asthma	1	1/178	0.56%	3/179	1.68%	0.34 [0.04, 3.19]	0.34
Cholecystitis	1	1/178	0.56%	0/179	0.00%	3.02 [0.12, 73.56]	0.5
Pancreatitis	1	1/178	0.56%	0/179	0.00%	3.02 [0.12, 73.56]	0.5
Decreased fibrinogen	1	1/178	0.56%	0/179	0.00%	3.02 [0.12, 73.56]	0.5
Furuncle	1	0/178	0.00%	1/179	0.56%	0.34 [0.01, 8.17]	0.5

Abbreviations: ALT: Alanine aminotransferase; AST: Aspartate aminotransferase; CI: confidence interval; ECG: Electrocardiogram; FGET: First generation EGFR tyrosine kinase inhibitor; P: Probability; TGET: Third generation EGFR tyrosine kinase inhibitor.

Table S6 Grade 3-5 adverse events.

Adverse events	Studies involved	TGET		FGET		Risk ratio [95% CI]	P
		Event/total	%	Event/total	%		
Hyponatremia	2	63/546	11.54%	6/540	1.11%	7.50 [0.54, 105.21]	0.13
Diarrhea	6	34/1249	2.72%	17/1243	1.37%	1.79 [0.89, 3.60]	0.02
Platelet count decreased	4	23/853	2.70%	3/851	0.35%	3.77 [0.85, 16.76]	0.0009
Increased blood creatine phosphokinase	3	15/574	2.61%	2/574	0.35%	2.76 [0.07, 115.54]	0.59
Pulmonary embolism	3	16/639	2.50%	2/636	0.31%	5.89 [1.53, 22.71]	0.005
ALT increased	6	31/1249	2.48%	99/1243	7.96%	0.25 [0.09, 0.68]	0.007
Lipid metabolism diseases	1	4/182	2.20%	1/180	0.56%	3.96 [0.45, 35.05]	0.22
Hypertension	4	18/853	2.11%	12/851	1.41%	1.59 [0.43, 5.87]	0.49
Anemia	5	20/982	2.04%	11/980	1.12%	1.63 [0.72, 3.66]	0.12
Pneumonia	4	17/853	1.99%	18/851	2.12%	0.98 [0.45, 2.14]	0.86
Renal symptoms	1	5/279	1.79%	1/277	0.36%	4.96 [0.58, 42.22]	0.14
Ejection fraction decrease	1	5/279	1.79%	1/277	0.36%	4.96 [0.58, 42.22]	0.14
Fatigue	3	12/728	1.65%	3/720	0.42%	3.38 [0.56, 20.26]	0.03
Decreased appetite	5	16/1071	1.49%	9/1064	0.85%	1.66 [0.72, 3.83]	0.17
Neutrophil count decreased	3	10/671	1.49%	1/671	0.15%	5.22 [1.14, 23.82]	0.03
Hypokalemia	3	10/671	1.49%	12/671	1.79%	0.84 [0.36, 1.95]	0.67
Pathological fracture	1	4/279	1.43%	2/277	0.72%	0.20 [0.01, 4.12]	0.3
White blood cell count decreased	4	12/853	1.41%	2/851	0.24%	3.77 [1.04, 13.60]	0.02
ECG QTc prolongation	4	12/853	1.41%	10/851	1.18%	1.22 [0.51, 2.88]	0.66
Gamma-glutamyl transferase increase	3	8/671	1.19%	6/671	0.89%	1.42 [0.24, 8.56]	0.6
Lymphocyte count decreased	2	5/457	1.09%	0/456	0.00%	5.45 [0.63, 47.00]	0.1
Deep vein thrombosis	2	5/461	1.08%	1/457	0.22%	2.84 [0.31, 25.75]	0.16
Cataract disorder	1	3/279	1.08%	1/277	0.36%	2.98 [0.31, 28.46]	0.34
Sepsis	1	3/279	1.08%	3/277	1.08%	1.99 [0.37, 10.75]	0.43
Peripheral sensory neuropathy	3	6/578	1.04%	1/572	0.17%	2.86 [0.52, 15.60]	0.12
Dermatitis acneiform	2	4/408	0.98%	1/406	0.25%	2.83 [0.43, 18.49]	0.24
AST increased	6	12/1249	0.96%	67/1243	5.39%	0.20 [0.11, 0.36]	<0.00001
Dyspnea	3	6/639	0.94%	4/636	0.63%	1.32 [0.36, 4.81]	0.55
Pericardial diseases	2	3/360	0.83%	3/359	0.84%	0.94 [0.07, 12.95]	1
Pleural effusion	3	5/639	0.78%	3/636	0.47%	1.27 [0.27, 5.94]	0.51
Headache	4	6/853	0.70%	0/851	0.00%	3.92 [0.83, 18.54]	0.08
Urinary tract infection	4	5/853	0.59%	2/851	0.24%	1.75 [0.41, 7.43]	0.35

Abnormal liver function	4	5/853	0.59%	16/851	1.88%	0.32 [0.12, 0.88]	0.2
Gingivitis	1	1/178	0.56%	0/179	0.00%	3.02 [0.12, 73.56]	0.5
Contrast agent allergy	1	1/178	0.56%	1/179	0.56%	1.01 [0.06, 15.95]	1
Epistaxis	1	1/178	0.56%	1/179	0.56%	1.01 [0.06, 15.95]	1
Protrusion of intervertebral disc	1	1/178	0.56%	0/179	0.00%	3.02 [0.12, 73.56]	0.5
Hyperkalemia	1	1/178	0.56%	2/179	1.12%	0.50 [0.05, 5.50]	0.57
Cholecystitis	1	1/178	0.56%	0/179	0.00%	3.02 [0.12, 73.56]	0.5
Decreased fibrinogen	1	1/178	0.56%	0/179	0.00%	3.02 [0.12, 73.56]	0.5
Increased amylase	1	1/178	0.56%	1/179	0.56%	1.01 [0.06, 15.95]	1
Nausea	6	7/1249	0.56%	2/1243	0.16%	2.43 [0.60, 9.84]	0.17
Rash	6	7/1249	0.56%	46/1243	3.70%	0.25 [0.07, 0.90]	<0.00001
Abdominal pain	3	3/639	0.47%	7/636	1.10%	0.54 [0.07, 4.11]	0.25
Weight decreased	4	4/853	0.47%	4/851	0.47%	0.95 [0.15, 5.85]	1
Upper respiratory tract infection	4	4/853	0.47%	4/851	0.47%	0.99 [0.24, 4.10]	1
Pancreatitis	2	2/457	0.44%	0/456	0.00%	3.00 [0.31, 28.71]	0.34
Cerebral infarction	2	2/457	0.44%	1/456	0.22%	1.60 [0.20, 12.94]	0.62
Pneumothorax	1	1/279	0.36%	2/277	0.72%	0.50 [0.05, 5.44]	0.57
Acute psychosis	1	1/279	0.36%	0/277	0.00%	2.98 [0.12, 72.80]	0.5
Acute myocardial infarction	1	1/279	0.36%	0/277	0.00%	2.98 [0.12, 72.80]	0.5
Anxiety	1	1/279	0.36%	0/277	0.00%	2.98 [0.12, 72.80]	0.5
Cognitive disorder	1	1/279	0.36%	0/277	0.00%	0.99 [0.06, 15.79]	1
Interstitial lung disease	1	1/279	0.36%	1/277	0.36%	0.99 [0.20, 4.88]	0.99
Paronychia	4	3/853	0.35%	7/848	0.83%	0.64 [0.17, 2.37]	0.27
Atrial fibrillation	3	2/639	0.31%	0/636	0.00%	3.00 [0.31, 28.71]	0.34
Chest discomfort	2	1/360	0.28%	2/359	0.56%	0.62 [0.08, 5.03]	0.62
Stomatitis	4	2/853	0.23%	5/848	0.59%	0.57 [0.13, 2.40]	0.35
Cystitis	2	1/457	0.22%	2/456	0.44%	0.74 [0.05, 10.64]	0.65
Hemoptysis	2	1/461	0.22%	4/457	0.88%	0.25 [0.03, 2.21]	0.21
Edema	2	1/461	0.22%	1/457	0.22%	0.99 [0.06, 15.79]	1
Pruritus	5	2/982	0.20%	0/980	0.00%	2.99 [0.31, 28.60]	0.34
Blood bilirubin increased	3	1/574	0.17%	1/574	0.17%	1.01 [0.06, 15.95]	1
Musculoskeletal pain	3	1/639	0.16%	2/636	0.31%	0.74 [0.05, 10.31]	0.66
Gastrointestinal diseases	3	1/639	0.16%	3/636	0.47%	0.51 [0.09, 2.96]	0.42
Dry skin	3	1/675	0.15%	4/669	0.60%	0.33 [0.05, 2.09]	0.24
Vomiting	4	1/853	0.12%	4/851	0.47%	0.54 [0.02, 14.19]	0.27
Oral ulcer	4	1/853	0.12%	2/851	0.24%	0.73 [0.05, 10.38]	0.65
Pyrexia	2	0/461	0.00%	1/457	0.22%	0.33 [0.01, 8.09]	0.5

Hyperuricaemia	1	0/182	0.00%	1/180	0.56%	0.33 [0.01, 8.04]	0.5
Constipation	4	0/768	0.00%	9/765	1.18%	0.05 [0.00, 0.89]	0.04
Chest pain	2	0/360	0.00%	2/359	0.56%	0.20 [0.01, 4.09]	0.29
Muscle spasms	1	0/129	0.00%	0/129	0.00%	-	-
Hematuria	1	0/182	0.00%	0/180	0.00%	-	-
Increased bilirubin conjugated	1	0/178	0.00%	1/179	0.56%	0.34 [0.01, 8.17]	0.5
Cough	3	0/639	0.00%	1/636	0.16%	0.33 [0.01, 8.09]	0.5
Weight increased	2	0/360	0.00%	1/359	0.28%	0.33 [0.01, 8.04]	0.5
Dizziness	1	0/182	0.00%	0/180	0.00%	-	-
Insomnia	1	0/279	0.00%	0/277	0.00%	-	-
Alopecia	1	0/279	0.00%	0/277	0.00%	-	-
Nasopharyngitis	1	0/279	0.00%	0/277	0.00%	-	-
Hyperglycemia	2	0/461	0.00%	2/457	0.44%	0.20 [0.01, 4.12]	0.3
Hypocalcemia	1	0/178	0.00%	1/179	0.56%	0.34 [0.01, 8.17]	0.5
Asthma	1	0/178	0.00%	2/179	1.12%	0.20 [0.01, 4.16]	0.3
Furuncle	1	0/178	0.00%	1/179	0.56%	0.34 [0.01, 8.17]	0.5
Arrhythmia	2	0/360	0.00%	1/359	0.28%	0.34 [0.01, 8.17]	0.5
Proteinuria	1	0/182	0.00%	0/180	0.00%	-	-
Hypoalbuminemia	2	0/493	0.00%	2/492	0.41%	0.20 [0.01, 4.12]	0.3
Blood lactate dehydrogenase increase	1	0/214	0.00%	0/215	0.00%	-	-
Elevated serum creatinine	1	0/214	0.00%	0/215	0.00%	-	-
Back pain	2	0/457	0.00%	0/456	0.00%	-	-
Elevated fibrin D-dimer	1	0/182	0.00%	0/180	0.00%	-	-

Abbreviations: ALT: Alanine aminotransferase; AST: Aspartate aminotransferase; CI: confidence interval; ECG: Electrocardiogram; FGET: First generation EGFR tyrosine kinase inhibitor; P: Probability; TGET: Third generation EGFR tyrosine kinase inhibitor.
